# Birds living near airports do not show consistently higher levels of feather corticosterone

**DOI:** 10.1093/conphys/coad079

**Published:** 2023-10-18

**Authors:** Renata D Alquezar, Lucía Arregui, Regina H Macedo, Diego Gil

**Affiliations:** PG em Ecologia, Instituto de Ciências Biológicas, Universidade de Brasília, 70919-970, Brasília, DF, Brasil; Departamento de Ecología Evolutiva, Museo Nacional de Ciencias Naturales (CSIC), José Gutiérrez Abascal, 2, 28006 Madrid, Spain; Departamento de Zoologia, Instituto de Ciências Biológicas, Universidade de Brasília, 70910-900, Brasília, DF, Brasil; Departamento de Ecología Evolutiva, Museo Nacional de Ciencias Naturales (CSIC), José Gutiérrez Abascal, 2, 28006 Madrid, Spain

**Keywords:** Bioacoustics, body condition, Brazil, neotropics, song frequency, stress

## Abstract

Noise represents a threat to human and wildlife health, triggering physiological and behavioral challenges to individuals living close to sources of extreme noise. Here, we considered airport environments as sources of potentially stressful stimuli for birds and tested if those living near airports are under higher physiological stress than birds living in quiet sites. We used measurements of CORT in feathers (CORT_f_) as a proxy of chronic stress. We evaluated 14 passerine and 1 non-passerine species, living near three Brazilian airports. We found that, across species, individuals with a better body condition had lower CORT_f_ concentration. At the species level, we found that CORT_f_ concentration was not consistently affected by airport noise. Comparing individuals living in quiet sites with those living near airports, we found that 2 species had higher and 2 had lower CORT_f_ concentrations near airports, while 11 species presented no significant differences between sites. At the population level, model selection indicated that the direction and strength of these differences are weakly related to species’ song frequency (peak frequency), as lower-frequency singers tended to present higher CORT_f_ levels at airport-affected sites. In summary, we were unable to find a consistent response among species, probably due to species-specific differences in their response to anthropogenic disturbances. Instead, we found that species might be affected differently according to their singing spectral frequency and that individuals in good body condition show lower CORT_f_, suggesting that this measure is consistent with lower physiological stress.

## Teaser

We found that individuals with better body condition had lower corticosterone levels in their feathers. Birds with lower frequency songs were more prone to be physiologically stressed in airport-affected sites. Our results suggest that bird species differ in the way they cope with living in a stressful environment.

## Introduction

Humans have not only dramatically modified the Earth’s landscapes in the past centuries but also significantly increased the levels of pollutants in the environment, endangering the welfare of both wildlife and people (Jarrige and Le Roux, 2020). In addition to chemical pollution, noise pollution is now recognized as a problematic aspect of anthropogenic development, attracting increased attention in the past decades (Shannon *et al.,* 2016). Noise pollution is known to influence aspects of wildlife behavior (Klett-Mingo *et al.,* 2016), disrupt reproduction (Halfwerk *et al.,* 2011) and change the dynamics of animal communities (Francis *et al.,* 2009). These impacts demand further inquiry concerning which aspects of noise pollution affect wildlife, providing the knowledge for effective mitigation and management actions (Barber *et al.,* 2009; Francis and Barber, 2013; Alquezar and Macedo, 2019).

Airport environments are affected by several disturbances, like extreme noise, air pollution, habitat degradation and habitat fragmentation, among others. Airports are a noticeable source of great amounts of noise, comprised by a wide spectrum of frequencies within the acoustic space at very high amplitude levels, jeopardizing the health and also communication among animals of different taxa (Gil *et al.,* 2015; Alquezar and Macedo, 2019), including humans. Previous studies have shown that humans exposed to airport noise pollution face increased risk of hypertension, psychological and sleep disturbances, increased catecholamine secretion and impaired cognitive performance (Stansfeld and Matheson, 2003; Stansfeld *et al.,* 2005; Jarup *et al.,* 2008; Finegold, 2010; Floud *et al.,* 2013).

Birds rely on vocal communication for the maintenance of social interactions (Hultsch and Todt, 1982; Catchpole and Slater, 2008) and noise can mask their most used song frequency bands (Wiley, 2013). Such masking can disrupt communication and reduce birds’ ability to perceive predators and to assess other risky situations (Quinn *et al.,* 2006; Klett-Mingo *et al.,* 2016), potentially inducing chronic stress (Kleist *et al.,* 2018). In birds, airport noise has been associated to community homogenization (Alquezar *et al.,* 2020b) and behavioral changes, such as vocal modifications (Gil *et al.,* 2015; Dominoni *et al.,* 2016; Sierro *et al.,* 2017) and foraging disruption (Klett-Mingo *et al.,* 2016).

Studies have suggested that, in urban environments, bird species are filtered based on their vocal characteristics (Francis *et al.,* 2011), as lower-frequency singers avoid noisy environments (Gomes *et al.,* 2021). Still, those species that remain in noisy areas might spend a greater amount of energy to maintain their vocal communication, either by increasing their song frequency and amplitude (Nemeth and Brumm, 2010) or by repeating their song more often in time (Sierro *et al.,* 2017), creating a stressful condition. In this sense, it is expected that birds singing at lower frequencies would be more prone to stress in noisy environments. As well, it is expected that birds adapted to urban conditions (more common and abundant in cities) would be less prone to be stressed by noisy environments.

One way to evaluate how wildlife is responding to noisy environments created by urbanization is by measuring the levels of glucocorticoid hormones (Sapolsky *et al.,* 2000; Romero, 2004) linked to the stress response. Long-term changes in these hormones may indicate whether a given population or an individual is chronically stressed (Dickens and Romero, 2013). The stress response can be defined as a set of behavioral and physiological changes that help an individual restore systemic homeostasis when exposed to noxious stimuli, such as predator exposure, shortage of food resources, competition and unfavorable climatic conditions, among other possibilities (Buchanan, 2000; Romero, 2004; Costantini, 2008; Dantzer *et al.,* 2014). In birds, corticosterone (CORT) is the glucocorticoid hormone analogous to cortisol in mammals. When exposure to threats is occasional, CORT release induced by the hypothalamic–pituitary–adrenal (HPA) axis helps individuals to deal with risky situations (Wingfield and Kitaysky, 2002). However, when exposure to noxious stimuli is continuous, the stress response becomes chronic and can lead to detrimental consequences to the individual’s breeding activities, survival, and cognitive ability (Sapolsky *et al.,* 2000; Kitaysky *et al.,* 2003).

Animal responses to stressful conditions have been studied and reviewed by many authors, but the field has yet to achieve a consensus with respect to the expected direction of the physiological responses (see reviews: Breuner *et al.,* 2008; Bonier *et al.,* 2009; Busch and Hayward, 2009; Bonier, 2012; Dickens and Romero, 2013). Meta-analytical approaches show that chronic stress cannot be assessed by a single measure of CORT level, although, integrative measures (e.g. feather or fecal CORT) show a clearer pattern than hormone instantaneous measures (e.g. blood), in particular as a response to anthropogenic stress (Dickens and Romero, 2013). Recent studies show varying results, where vertebrates exposed to urban environments either present higher levels of plasmatic and fecal CORT (baseline only, instantaneous and integrated measures) (Dantzer *et al.,* 2014) or no effects relative to baseline and stress-induced CORT levels (Iglesias-Carrasco *et al.,* 2020). The latter study argues that the lack of detectable responses to urban habitats reflects the fact that most research has focused on the urban habitat per se, but not on the specific anthropogenic pressures that may affect wildlife in urban habitats (e.g. noise, light, air pollution, habitat quality).

Considering studies that have focused on the specific impacts of noise pollution on birds, evidence suggests that individuals from species that are common in cities (i.e. urban adapters or exploiters) are more likely to present no differences in baseline CORT levels and/or in body condition when compared to individuals of the same species in quiet areas (e.g. song sparrow, *Melospiza melodia*: Grunst *et al.,* 2014; house sparrow, *Passer domesticus*: Angelier *et al.,* 2016; and house wren, *Troglodytes aedon*: Davies *et al.,* 2017). This lack of baseline CORT level differences can be either because the species is insensitive to these disturbances (Grunst *et al.,* 2014; Angelier *et al.,* 2016) or because the species have adapted to the stressful condition (Davies *et al.,* 2017).

In the past decade, several techniques have been developed to extract and measure corticosterone levels in feathers (CORT_f_) (Bortolotti *et al.,* 2008). CORT_f_ is an integrated measure of the hormone levels, representing a long-term hormonal response by the HPA axis. It is less invasive than blood measurements, not influenced by instantaneous researcher-bird manipulation and is considered a useful technique for studies in avian stress physiology and conservation (Bortolotti *et al.,* 2009b; Sheriff *et al.,* 2011; Fairhurst *et al.,* 2013; Dantzer *et al.,* 2014; Romero and Fairhurst, 2016). Research has shown that plasmatic CORT metabolites are deposited in feathers during their annual growth, when feather structures are being irrigated by blood (Jenni-Eiermann *et al.,* 2015). When feather growth ends, the keratinized tissue preserves the stored CORT (Bortolotti *et al.,* 2008). Once feathers are collected and stored in museums and collections, the stored CORT levels remain stable for several years, resistant even to heat and freezing (Bortolotti *et al.,* 2009a).

Using measurements of corticosterone in feathers (CORT_f_), in this study, we tested the main hypothesis that Neotropical birds living in the vicinity of airport environments are under higher physiological stress than those living in quiet environments. Due to a concern about whether CORT_f_ (or baseline CORT) is an appropriate proxy of stress levels (Dickens and Romero, 2013), we first investigated whether an individual’s CORT_f_ concentration is related to its Body Condition Index, given that body condition has been suggested as the best measure of chronic stress (Dickens and Romero, 2013).

At the species level, we tested whether differences in CORT_f_ concentrations between the two types of areas (airport-affected and quiet control sites) follow the same general trend, or alternatively, are species-specific. At the population level, we tested whether song frequency and preference for urban environments could explain the direction and strength of the response. In this context, we predict that species with higher song frequency and stronger preference for urban environments would show a weaker CORT_f_ response.

## Materials and Methods

### Study sites

**Figure 1:**
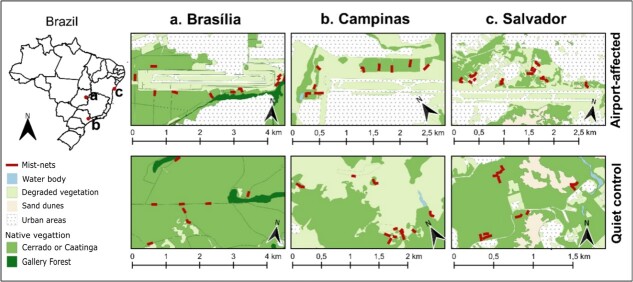
Location of study sites in Brazil, including airport-affected and quiet control sites for each studied region.

We collected data in patches of native vegetation in the immediate surroundings of three airports in Brazil (airport-affected sites). For each airport-affected site, we selected a nearby quiet control site (8–17 km distant from the airport), with a similar vegetation structure (Fig. 1). Airports were selected based on their high aircraft activity and the availability of native vegetation around the lanes. Airport-affected and quiet control sites are located in three different regions of Brazil (Brasília, Campinas and Salvador), 790–1450 km apart from each other.

**Table 1:** Characterization of study sites.

	BRASÍLIA		CAMPINAS		SALVADOR	
	Airport	Control	Airport	Control	Airport	Control
Landscape structure						
% Native	73.60	94.35	37.22	48.03	37.60	83.10
% Degraded and urbanized	26.40	5.65	62.78	51.97	62.40	16.90
Noise levels						
Minimum amplitude (dB)	38	35	40	36	37	36
Mean amplitude (dB) ± sd	52 ± 8	38 ± 2	54 ± 8	41 ± 3	51 ± 8	43 ± 3
Maximum amplitude (dB)	86	54	92	60	92	70

Landscape structure: percentage of landscape classes within each study site. Noise levels: values of minimum, mean and maximum amplitudes (dB = decibels) for each study site, during sunrise time, in frequency range of 1–2 kHz (technophony). Table information adapted from Alquezar *et al.* (2020b).

The six study sites are identified according to whether they are an Airport (AIR) or a quiet control (CONT) site, and by the region/city where they are located (Brasília, Campinas or Salvador). At the Brasilia region (Distrito Federal) we selected the Presidente Juscelino Kubitschek International Airport (AIR_Bras: −15.872°, −47.919°) paired with a quiet control site, the “Parque Nacional de Brasília” (CONT_Bras: −15.728°, −47.951°). At the Campinas region (São Paulo state) we selected the Viracopos International Airport (AIR_Camp: −23.006°, −47.141°) and a private farm named “Fazenda Santa Maria” (CONT_Camp: −23.098°, −47.130°). And at the Salvador region (Bahia state) we selected the Luís Eduardo Magalhães International Airport (AIR_Sal: −12.916°, −38.338°) and a residential area with large and protected areas named “Condomínio Buscavida” (CONT_Sal: −12.859°, −38.270°).

In Alquezar *et al.* (2020b) we provide a characterization of each study site regarding landscape structure and noise levels (Table 1). These characteristics will be summarized here for better understanding, but detailed methodology must be consulted in the original publication. Landscape structure: each airport-affected site and its correspondent quiet control site contain similar vegetation structure (biome-wise), but airport-affected sites present higher proportions of degraded and urbanized landscapes than quiet control sites, while the latter had higher proportions of native landscape. Noise levels: we evaluated mean noise amplitude within 1–2 kHz frequency band, where most technophony is concentrated (Towsey *et al.,* 2014). In all pairs of sites, noise levels are higher in airport-affected sites, the difference being higher in the Brasilia region and lower in the Salvador region.

### Data collection

We mist-netted wild birds at all sites and collected two to three feathers from each individual’s tail. Captured birds were banded with numbered metal bands (provided by the Brazilian Banding National System- CEMAVE-SNA/ICMBio) and measured for body weight and tarsus, wing and tail lengths. Collected feathers were stored in identified paper bags for later analysis. All procedures were submitted to and approved by the University of Brasilia Ethics Committee (129 022/2015 CEUA UnB), by the Brazilian System of Authorization and Biodiversity Information (SISBIO 42578), by the Brazilian Banding National System (CEMAVE-SNA 3856) and by the Brazilian National System for Management of Genetic Patrimony and Associated Traditional Knowledge (SISGEN AAA265A).

Captures were conducted using 10 mist-nets, during 10 mornings at each site, opened for five hours during the morning. Additionally, we conducted directional captures using isolated nets and playback stimuli to attract birds (Fokidis *et al.,* 2009) of specific species, to increase sample sizes, balancing the number of individuals captured in airport-affected and quiet control sites. In airports-affected sites, mist-nets were set at a maximum distance of 250 m from flight lanes, and in quiet control sites, they were set without restrictions. Both sites in each region were sampled in alternate weeks within each region’s avian breeding season. Brasilia’s region was sampled during October 2014 and November 2015; Campinas’s region, November–December 2014; and Salvador’s region, December 2015 to January 2016.

Most birds in Brazil molt their remiges and rectrices following their breeding period (Marini and Durães, 2001; Silveira and Marini, 2012). The breeding period is concentrated at the rainy season (around September to January) while molt is expected to occur in the end of the rainy season (after January). Considering our fieldwork period (breeding period), we expect that captured birds had molted their feathers in the previous year. Among the species included in the analysis, three are migrants (*Elaenia chiriquensis*, *Myiarchus swainsoni* and *Volatinia jacarina*). All these three migrant species are known to breed (Marini *et al.,* 2012) and molt in central Brazil (Queiroz, 2008; Silveira and Marini, 2012), where they were sampled. Awareness of these molting patterns provided us with the certainty that collected feathers were molted at the study sites where these migratory birds were sampled (Brasília and Campinas).

Feathers were collected exclusively from adult birds, thus we presume they grew within the same annual time interval for each species. We did not consider possible differences related to sex, because the majority of analyzed species have no sexual dimorphism (exceptions are *V. jacarina* and *Coryphospingus cucullatus*).

### Feather processing and CORT_**f**_ analyses

Each feather was individually weighed and measured for length (excluding calamus), since both methods are used in the literature to calculate concentration in steroid immunoassays (see below). Afterward, feathers were cut into very small pieces (< 2 mm). For each sample, we used 1–3 feathers from the same individual, and this material was weighed to the nearest 0.001 g with a high precision balance. In cases where feathers from a single individual did not provide enough material to produce a reliable assay (*V. jacarina*, *Coereba flaveola* and some *Troglodytes musculus*), we pooled feathers from two to three individuals to compose a single sample (up to six feathers). Pooled samples were restricted to individuals from the same species from the same site.

Methanol-based hormone extraction (corticosterone metabolites) was conducted following Bortolotti’s protocol (Bortolotti *et al.,* 2008, 2009a), including adjustments suggested by Lattin *et al.* (2011). The minced feather samples were placed in silanized glass tubes, to which we added 6 ml of methanol (HPLC gradient grade, Prolabo (VWR), PA, USA). The samples were then placed in a sonicating water bath at room temperature for 30 min, followed by incubation at 50°C overnight (18 hours) in a shaking water bath. We added an additional volume of 2 ml of methanol to the samples, which were vigorously vortexed for 10 min, and then separated the liquid from feathers using a disposable syringe and a plug of synthetic polyester fiber (0.45 μm) for filtration. The methanol extract was placed in a new silanized glass tube and evaporated in a fume hood using a stream of nitrogen. After complete evaporation, we added 300 μl of steroid free serum (DRG Instruments GmbH, Marburg, Germany), quickly centrifuged the samples, stored the new extracts in plastic tubes and froze them at −20°C for subsequent corticosterone metabolites analysis. Hereafter, corticosterone metabolites are referred as corticosterone (CORT).

We ran a corticosterone enzyme immunoassay (EIA) using a CORT specific kit (DRG Instruments GmbH, Marburg, Germany), and read the plates in a Biotek spectrophotometer (Biotek Instruments, Inc, Winooski, VT, USA). Absorbance was read at a wavelength of 450 nm. Samples were assayed in duplicates, and CORT concentration was averaged from the two samples after regression of the standard curve of each plate. Intra-assay variation was calculated as the mean variation between duplicates (8.7%), and inter-assay variation was calculated as the mean of mean variation between lower and higher control concentrations of each plate (12.1%). Linearity of serial dilutions of sample pools was adequate. Laboratory analyses were conducted in the Ecophysiology Lab of the Museo Nacional de Ciencias Naturales (Madrid, Spain), from September to November of 2016.

Most studies indicate that expressing hormone levels in terms of feather length is a more appropriate approach (Bortolotti *et al.,* 2008, 2009a; Jenni-Eiermann *et al.,* 2015), however, some studies still express hormone values in terms of feather mass (Koren *et al.,* 2012; Lendvai *et al.,* 2013). Based on our own tests, the CORT concentrations based on feather mass and length were highly and significantly correlated (see Supplementary Material A for results; linear regression: F_2,593_ = 1561, *P* < 0.001, r^2^ = 0.72, intercept = 4.58^−16^, slope = 0.851). Given the high correlation between the two measurements, we chose to present feather hormone values as a function of each sample’s added lengths of all feathers (pg mm^−1^), following previous literature (Bortolotti *et al.,* 2009a; Lattin *et al.,* 2011).

### Analysis

All analyses were performed in R (R Core Development Team, 2022) and statistical significance was considered at *P* < 0.05. Variables were tested for normality, box-cox transformed and scaled. All model significance was calculated with a post-hoc analysis of deviance (type III). To run these analyses we used packages “*lme4*” (Bates *et al.,* 2014), “*car*” (Fox and Weisberg, 2011), “*MuMIn*” (Barton, 2016) and “*AID*” (Asar *et al.,* 2017), as well as RStudio interface (RStudio Team, 2020). For figures we used “*ggplot2*” (Wickham, 2016) and “*sjPlot*” (Lüdecke, 2020), as well as Inkscape (Inkscape Project, 2020) for graphic editing.

#### Body condition

A Body Condition Index was calculated for individuals for which we had CORT_f_ and body measurements (body weight, tarsus, wing and tail length), comprising a dataset of 10 species and 308 samples. First, we summarized body size of each species using a principal component analysis (PCA) based on tarsus, wing and tail lengths. Then we ran a regression between body weight and body size (PCA’s first axis) and used the regression’s residuals as our Body Condition Index (Schulte-Hostedde *et al.,* 2005). Using this dataset, we investigated whether an individual’s CORT_f_ concentration is related to its Body Condition Index (data available in Supplementary Material B). Here, higher index values represent individuals with better body condition. Finally, we ran a linear mixed model (LMM) using CORT_f_ concentration as response variable and Body Condition Index as predictor. As random effect we included species ID and plate ID.

#### Species’ responses

To understand species’ response to different site types (airport vs. control), we fitted an exploratory LMM (global model 1), using CORT_f_ as the response variable. The site type (airport or control) and species ID (*n* = 15 species) were included as predictors, as well as their interaction. The variable Region was included as a covariate and Plate id as random effect. For this analysis we used the full dataset (*n* = 595). The LMM was followed by a post-hoc analysis of deviance (ANOVA).

Subsequently, we fitted models for each species to assess species-specific responses. Similarly to the previous model, we used CORT_f_ as the response variable and Site type (airport or control) as the predictor. We fitted simple LMs when samples were in only one plate or LMMs when samples were in more than one plate. For species with data from more than one region, we added region as covariate when the inclusion of this variable reduced models’ Akaike’s Information Criteria (AIC). Analysis script and output are available as Supplementary Material C.

#### Population responses

To understand whether population trends in CORT_f_ concentration can be explained by species´ characteristics, we ran a model selection using the variable CORT_f_ effect-size as the response variable and region, song frequency and degree of urbanity as predictor variables (explained below). We used a dataset of 512 samples, comprising 14 species and 18 populations. Population was defined as a group of individuals of the same species, captured in the same region (BRAS, CAMP or SAL). The samples were chosen based on a minimum number of five samples per site. Given the exploratory character of this analysis, we used “dredge” function to summarize the best models, ranking them by increasing Akaike’s Information Criteria (AICc) and considering models within ΔAIC < 2. We also accessed variables’ significance. Analysis script and output are available as Supplementary Material D.

CORT_f_ effect-size: For each population, we calculated CORT_f_ effect-size as the standardized effect difference between CORT_f_ concentration found in airport-affected and quiet control sites (i.e. Hedge’s *d;*Nakagawa and Cuthill, 2007). Positive effect sizes indicate higher CORT_f_ concentration in the airport-affected site relative to quiet control sites and negative effect-sizes indicate lower CORT_f_ concentration in airports-affected relative to quiet control sites (raw means and effect-sizes available in Supplementary Material D).

Song frequency Index: This index represents each species’ peak frequency corrected by body weight. Peak frequency is the frequency in which species concentrate most of the song energy, and differently from minimum frequency, this measure is less affected by noise masking. To obtain these values, we selected a minimum of five recordings of each species (RDA unpublished data) and used Raven Pro 1.5 (Center for Conservation Bioacoustics, 2019) to measure the acoustic parameters of a total of 20 songs from each species, using a FFT of 1024. All selected recordings are from quiet control sites. As species’ song frequency has been shown to be affected by species’ body size (Ryan and Brenowitz, 1985; Demery *et al.,* 2021), we corrected our peak frequency values by mean species body weight measured in each studied region. To do so, we ran a regression between peak frequency and body weight and used the regression’s residuals as our Song Frequency Index (used values available in Supplementary Material D).

Degree of urbanity: The degree of association of each species to urban environments was represented by the degree of urbanity. This variable was calculated using eBird (eBird, 2017) citizen data and details are provided in Alquezar *et al.* (2020a). Here, positive values are associated with species that exhibit some degree of preference for urban habitats while negative values are associated with species that exhibit some degree of avoidance of urban environments (values available in Supplementary Material D).

## Results

A total of 1187 individuals were captured and had their feathers collected in the six sampled sites, totaling 124 species. Due to the reduced sample size for some species, after fieldwork completion we decided to keep only those species that had at least five samples for each site type (AIR or CONT). Doing so, our analysis used data for 15 species, totaling 595 samples (raw data available in Supplementary Material B). The feather CORT concentrations (CORTf) varied from 0.76 pg mm-1 to 182.86 pg mm-1 (mean = 7.36 pg mm-1; sd = 12.14). The 15 species analyzed belong to one non-passerine order and seven passerine families, including suboscines (five species) and oscines (nine species). All species are reasonably common in urban environments.

The non-passerine species is the Swallow-tailed hummingbird (*Eupetomena macroura*, *n* = 17). The Passerines include Suboscines (suborder Tyrannii): sooty-fronted spinetail (*Synallaxis frontalis*, *n* = 25), plain-crested elaenia (*E. cristata*, *n* = 33), lesser elaenia (*E. chiriquensis*, *n* = 59), swainson’s flycatcher (*M. swainsoni, n* = 32), great kiskadee (*Pitangus sulphuratus*, *n* = 64); and Oscines (suborder Passeri): rufous- browed peppershrike (*Cyclarhis gujanensis*, *n* = 20), southern house wren (*T. musculus*, *n* = 67), pale-breasted thrush (*Turdus leucomelas*, *n* = 72), rufous-bellied thrush (*Turdus rufiventris*, *n* = 13), rufous-collared sparrow (*Zonotrichia capensis*, *n* = 25), sayaca tanager (*Thraupis sayaca*, *n* = 53), blue-black grassquit (*V. jacarina*, *n* = 62), red-crested finch (*C. cucullatus, n* = 30) and bananaquit (*C. flaveola*, *n* = 23 samples).

### Body condition

**Figure 2:**
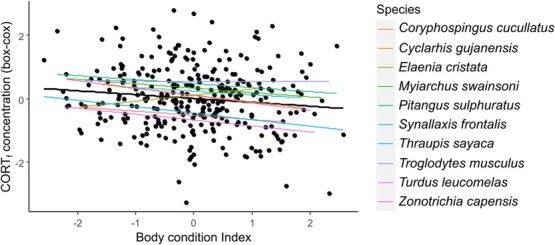
Relationship between CORT_f_ concentration and Body Condition Index. Colored lines indicate each species pattern and black line indicates model pattern.

Here we tested whether an individual’s CORT_f_ concentration is related to its Body Condition Index. If CORT_f_ reflects chronic stress levels suffered by birds, we could expect to find lower levels of CORT_f_ in individuals with higher body condition (i.e. healthier individuals). Using a dataset of 10 species comprising 308 samples, we ran a mixed model with Species id and Plate id as random factors and Body Condition Index as a predictor. As expected, we found a significant association between individual’s CORT_f_ concentration and Body Condition Index (LMM estimate: -0.155; X^2^ = 9.870; df = 1; *P* = 0.001): individuals with higher body condition showed decreased CORT_f_ concentrations. The pattern is consistent for most species (Fig. 2).

### Species’ responses

For the species response analysis, we used a dataset of 15 species comprising 595 samples. We addressed the question of whether there was a consistent difference in CORT_f_ concentration between birds in airport-affected vs. quiet control sites, and if this difference existed, whether it varied depending on the species. We found a significant interaction between site type (airport vs. control) and species ID (*post-hoc* Anova: *P* < 0.001), indicating that differences in CORT_f_ concentration depend on species-specific differences and site types. Analyses also indicated a significant difference between regions (*post-hoc* Anova: *P* < 0.001).

**Figure 3:**
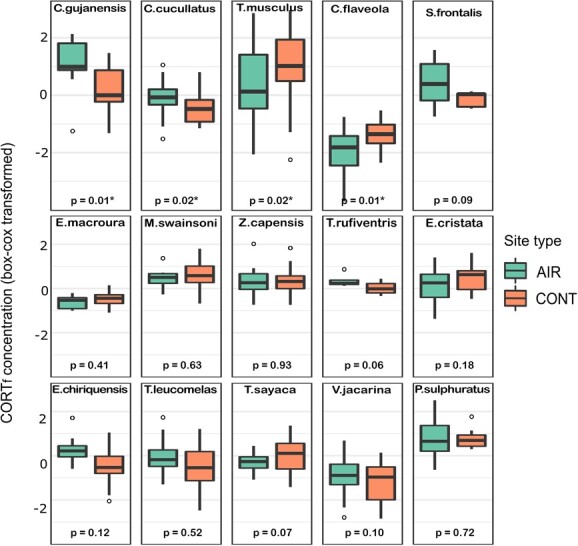
Species and site type interaction from species-specific analyses. Black circles represent outliers. The boxes show the median, interquartile range, and whiskers (indicating the 90th and 10th percentiles) for each species.

In the species-specific models, two species presented significant increased CORT_f_ concentration, and two species presented reduced CORT_f_ concentration in the airport-affected sites (Fig. 3), compared to the quiet control sites. The rufous-browed peppershrike (*C. gujanensis*; *P* = 0.01) and the red-crested finch (*C. cucullatus*; *P* = 0.02) presented CORT_f_ increases. The southern house wren (*T. musculus*; *P* = 0.02) and the bananaquit (*C. flaveola; P* = 0.01) presented decreases in CORT_f_ in the airport-affected sites. The remaining species presented no significant changes in CORT_f_ concentration between airport-affected and quiet control sites (Tables 2 and 3).

**Table 2:** Species-specific models (LMs), for five species that were analyzed in a single plate, thus have no random effect included.

CORT_f_ concentration (pg mm^−1^)								
	Estimate	SE	*t* value	*P* value	Airport	*N*	Control	*N*
*E. macroura* (SAL)								
Intercept	0.220	0.35	0.617	0.546	3.14 ± 0.5	9	3.47 ± 0.8	8
Site type-airport	−0.415	0.49	−0.848	0.410				
								
*S. frontalis* (BRAS and CAMP)								
Intercept	−0.539	0.36	−1.487	0.151	8.03 ± 5.3	18	4.18 ± 0.7	7
Site type-airport	0.749	0.42	1.753	0.093				
								
*M. swainsoni* (BRAS and CAMP)								
Intercept	0.0586	0.21	0.270	0.789	6.93 ± 2.7	10	8.39 ± 5.0	22
Site type-airport	−0.186	0.38	−0.483	0.633				
								
*Z. capensis* (BRAS and CAMP)								
Intercept	0.012	0.24	0.049	0.962	8.65 ± 9.1	8	7.16 ± 4.9	17
Site type-airport	−0.037	0.43	−0.086	0.932				
								
*T. rufiventris* (ALL)								
Intercept	−0.404	0.31	−1.296	0.221	5.99 ± 1.5	5	4.68 ± 0.9	8
Site type-airport	1.052	0.50	2.089	0.060				

Raw means of CORTf concentration and sample size (*N*) in airport-affected and quiet control sites included. Models include only site type as predictor. Model’s statistics = *t* student. Complete model’s output available as Supplementary Material D.

**Table 3:** Species-specific models (LMMs), for 10 species that were analyzed in multiple plates, thus have random effect included.

CORT_f_ concentration (pg mm-1)
	Estimate	SE	X^2^	df	*P* value	Airport	*N*	Control	*N*
**CORT**_**f**_ **~ site type + (1|plate id)**									
*E. cristata* (BRAS)									
Intercept	0.161	0.32	0.242	1	0.622	5.99 ± 3.3	16	7.67 ± 4.3	17
Site type-airport	−0.456	0.34	1.753	1	0.185				
									
*E. chiriquensis* (BRAS)									
Intercept	−0.375	0.39	0.914	1	0.339	5.90 ± 3.5	22	3.92 ± 2.1	37
Site type-airport	0.583	0.38	2.353	1	0.125				
									
*C. flaveola* (SAL)									
Intercept	0.616	0.66	0.863	1	0.352	1.66 ± 0.6	9	2.17 ± 0.5	14
Site type-airport	−0.944	0.39	5.811	1	0.015				
									
*T. sayaca* (CAMP and SAL)									
Intercept	0.280	0.20	1.793	1	0.180	3.92 ± 1.0	31	5.44 ± 2.9	22
Site type-airport	−0.478	0.27	3.065	1	0.079				
									
*V. jacarina* (BRAS and CAMP)									
Intercept	−0.345	0.29	1.345	1	0.246	3.97 ± 1.4	48	2.52 ± 1.1	14
Site type-airport	0.484	0.30	2.583	1	0.108				
									
*C. gujanensis* (ALL)									
Intercept	−0.249	0.49	-	-	-	15.55 ± 11.4	10	6.46 ± 4.3	10
Site type-airport	0.937	0.38	5.807	1	0.015				
									
*P. sulphuratus* (ALL)									
Intercept	−0.175	0.43	0.161	1	0.687	12.44 ± 12.7	54	9.11 ± 4.9	10
Site type-airport	0.121	0.35	0.121	1	0.727				
									
**CORT**_**f**_ **~ site type + region + (1|plate ID)**									
*T. musculus* (ALL)									
Intercept	1.105	0.19	-	-	-	13.64 ± 24.3	35	23.27 ± 34.7	32
Site type-airport	−0.420	0.18	4.937	1	0.026				
Region-CAMP	−1.047	0.23	41.648	2	<0.001				
Region-SAL	−1.508	0.23							
									
*T. leucomelas* (ALL)									
Intercept	0.701	0.43	-	-	-	4.983 ± 3.35	41	4.19 ± 2.6	31
Site type-airport	0.178	0.27	0.405	1	0.524				
Region-CAMP	−1.231	0.46	9.473	2	0.008				
Region-SAL	−1.133	0.37							
									
*C. cucullatus* (BRAS and CAMP)									
Intercept	1.135	1.22	-	-	-	4.77 ± 2.0	20	3.73 ± 1.7	10
Site type-airport	0.984	0.43	5.071	1	0.024				
Region-CAMP	−0.823	0.55	2.202	1	0.137				

Raw means of CORTf concentration and sample size (*N*) in airport-affected and quiet control sites included. Models include site type as predictor and region as covariate when the inclusion of this variable reduced models’ AIC. Model’s statistics = Chi-squared. Complete model’s output available as Supplementary Material D.

It is worth mentioning that the southern house wren (*T. musculus*), the pale-breasted thrush (*T. leucomelas*) and the red-crested finch (*C. cucullatus*), maintained region as an important variable after AIC comparison. However, region was considered significant for the southern house wren (*P* < 0.001) and the pale-breasted thrush (*P* = 0.008), but not for the red-crested finch (*P* = 013).

### Population’s responses

**Figure 4:**
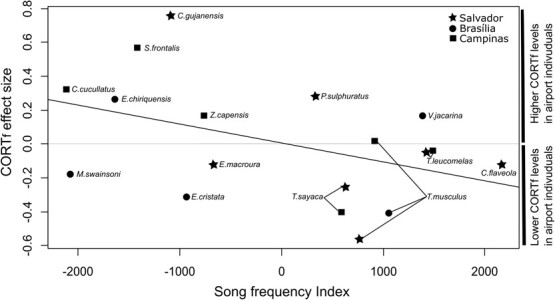
CORT_f_ effect-size and Song frequency Index relationship from population’s response model.

Given the disparity of responses to Site type in CORT_f_ concentration, we explored whether these differences between populations are affected by region, Song frequency Index and degree of urbanity. To this end, we applied a meta-analytical approach, using the effect-sizes of our dataset of 18 populations (14 species) comprising 512 samples. Model selection was run based on a full LM model resulting in the selection of two models (∆AIC < 2), the first including Song Frequency Index whereas the second option was the null model (Table 4). The general trend shows that species with a lower Song Frequency Index presented greater positive differences in CORT_f_ concentration, with a higher CORT_f_ concentration in airport-affected sites compared with quiet control sites (Fig. 4). Although Song Frequency Index was kept in the model, this variable was close to significance but not significant for the averaged model (*P* = 0.07). Degree of urbanity was not kept in the model as a useful predictor of CORT_f_ differences between sites.

**Table 4:** Model selection results for CORT_f_ effect-size (population’s response).

LM full model: CORT_f_ effect size~ Region + Song frequency + Degree of urbanity				
Selected models	df	AICc	∆AIC	Weight
Song frequency	3	16.3	0.00	0.457
Null	2	17.1	0.76	0.312
Song frequency + degree of urbanity	4	19.4	3.05	0.099

Only models with ∆AIC < 2 were considered suitable.

## Discussion

Stress induced by noise has been investigated in recent years for a few bird species, but results are not congruent, although Injaian *et al.* (2020) indicated that urban avoiders are potentially more sensitive to noise. Here, we evaluated corticosterone deposited in feathers as a long-term measure of the stress response in 15 bird species living in Brazilian airport-affected and quiet control sites. We considered that airport environments exhibit several disturbances, with noise as the most extreme and which is absent in other disturbed landscapes. First, we found that individuals with better Body Condition showed lower CORT_f_ concentration; we can thus consider body condition as a good predictor of CORT_f_. Second, we found that species’ responses were not consistently affected by airport noise, although four species showed either higher or lower CORT_f_ concentration in the airport-affected sites. At the population level, there was a weak indication that species with lower Song Frequency present higher CORT_f_ concentration in airport-affected sites.

Body condition has been used as an index of an individual’s quality and reproductive success (Milenkaya *et al.,* 2015; Montreuil-Spencer *et al.,* 2019). Here, we evaluated whether CORT_f_ concentration is related to Body Condition Index in our studied individuals, considering that feathers were grown in the previous 12 months, but the noxious impact of the airport environment is similar between years. The relationship found indicates that individuals with a better body condition (healthier) have lower CORT_f_ levels, that is, birds that experienced lower circulating CORT during feather growth were in better body condition at the time of capture. It is important to observe that feathers were molted in the year before and are expected to be molted again within 1–2 months after data collection. Thus, we are assuming that the stressful environment (chronic stress) is repeatable in time and affects birds throughout their lives, not leading to great changes in the bird’s season-specific body condition.

This finding agrees with a study that associates body condition with baseline and stress-induced levels of plasmatic CORT in kittiwakes *Rissa tridactyla* (Kitaysky *et al.,* 1999); as well as a study that associates body condition with feather CORT levels in tree swallows (*Tachyneta bicolor*) (Harms *et al.,* 2010). A meta-analysis about chronic stress found that the best physiological predictor of chronic stress of any kind, both in experimental and correlative studies, is body mass (Dickens and Romero, 2013), which typically decreases when animals are subjected to chronic stress. Given that CORT_f_ is an integrative measure that lasts long after the stress has been experienced, we can only rely on it to evaluate a stressful situation experienced during feather growth. It is necessary to be aware that the relationship between chronic stress *vs*. body condition and acute stress *vs*. body condition must be different because the information stored in feathers is relative to a restricted period of time. In this way, our results lend support to the use of CORT_f_ as a reliable index of chronic stress (when noxious stimuli are continuously present).

Species-specific responses were highly variable. We found 2 species with higher CORT_f_ concentration in the airport-affected sites, 2 species with lower CORT_f_ concentration in the airport-affected sites and 11 species presenting no differences between airport-affected and quiet control sites. Species-specific responses to noisy environments can vary greatly (Francis and Barber, 2013), presenting a difficult and additional obstacle to establishing an expected physiological response to acoustic disturbance. The observed increased CORT_f_ concentration in birds living near airports is easier to understand, since CORT_f_ levels represent the accumulation of stress responses during the timeframe of feather growth and increased levels of this hormone is a typical response to noise reported for birds, fishes, and humans (Evans *et al.,* 2001; Anderson *et al.,* 2011; Crino *et al.,* 2011; Strasser and Heath, 2013). This increased hormone level is classified as a stage of homeostatic overload by Romero *et al.* (2009), representing changes that exceed the normal reactive scope and cause physiological disruption (chronic stress). This overload can result in greater individual susceptibility to parasite infections (Bortolotti *et al.,* 2009b) and decreased survival in the wild (Koren *et al.,* 2012).

On the other hand, decreases in CORT concentration are less commonly reported in the literature, but according to Romero *et al.* (2009), this may be observed in individuals experiencing homeostatic failure. This is a more severe state of chronic stress, representing changes that cause physiological disruption and death, with hormone levels below the predictive homeostasis range, which has also been shown to be related to lower reproductive success (Kleist *et al.,* 2018). Reductions in basal and stress-induced plasmatic CORT levels have been reported for captive and free-living birds (Rich and Romero, 2005; Cyr *et al.,* 2007; Cyr and Romero, 2007), and these changes were associated with reduced body weight and lower fledging success. The authors discarded the possibility that these results could be due to habituation to the stressor and exhaustion, and claimed that the response must be a controlled systemic downregulation of HPA activity (Rich and Romero, 2005). Explanations relative to reduced baseline CORT levels observed for other taxa include (1) acclimation to a repeated stressor, (2) a real reduction of stress conditions due to higher resource availability or (3) a state of chronic stress (French *et al.,* 2008). Considering the biology of species that presented decreased CORT_f_ here, both are very small birds, highly abundant in urban environments in Brazil. Due to the small population size found for *T. musculus* in all three studied airports, we speculate that this species is not dealing well with the airport condition and might be in a chronic stress state.

For the remaining 11 species that presented no changes in CORT_f_ concentration, we speculate that they are habituated to noisy conditions. Based on the possible explanations for either increased or reduced levels of CORT_f_ concentration, we assume that CORT_f_ levels must be influenced by factors other than only extreme noise, and that species are not consistently affected by noise in the same way. We highlight the fact that the analyzed species are reasonably common in urban environments since they are able to live in airport environments and deal with the associated environmental degradation. We emphasize that these results should be taken cautiously when considering species that are more sensitive to urban environments. Species that are of conservation concern such as the northern spotted owl (*Strix occidentalis caurina*) (Hayward *et al.,* 2011) and the greater sage-grouse (*Centrocercus urophasianus*), can be severely affected by noise, as shown in noise playback experiments (Blickley *et al.,* 2012).

When evaluating population aspects that could explain the species-specific responses, we expected that species with lower song frequency would be more affected by noise due to frequency masking and jeopardized communication (Gil and Brumm, 2014). Our model selection showed that Song Frequency Index (peak frequency corrected by body weight) is a potential explanation for increased hormonal responses observed in airport-affected sites, denoting that species that vocalize in lower frequencies are more prone to being physiologically stressed in airport-affected sites. Although the relationship that we found is neither strong nor significant, it is in accordance with previous studies showing that anthropogenic noise filters out bird species that have low song frequencies (Francis *et al.,* 2011; Proppe *et al.,* 2013; Cardoso, 2014). Species that vocalize at lower frequencies may be burdened in noisy environments, having to shift their song frequency (Luther *et al.,* 2016), increase their vocal amplitude (Schuster *et al.,* 2012), and even avoid noisier areas (McClure *et al.,* 2013, 2017; Proppe *et al.,* 2013). We also expected that species that exhibit a preference for urban habitats would be less affected by noise (Injaian *et al.,* 2020). However, this variable was not included in the selected models, probably because the analyzed species do not show great differences related to urban sensitivity.

The four species that presented increased or decreased CORT_f_ levels in the species-specific analysis belong to the suborder Passeri (known as oscines or songbirds). The oscines produce more complex songs than suboscines. They have a more complex syringe, learn their songs, have a more diverse repertoire and higher ability to modify their songs (Catchpole and Slater, 2008). This phylogenetic similarity in the species that presented some degree of hormonal response to airport environments, allow us to speculate that oscine birds might be more affected by this environmental disturbance (noise). In the same way that population-specific analysis indicated a possible relationship between CORT_f_ levels and song frequency, it is possible that song complexity might be an important variable to define which birds have their CORT levels affected by noise (Ríos-Chelén *et al.,* 2012). This aspect should be tested by future studies.

In summary, our study shows that CORT_f_ concentration and Body Condition Index are related, providing support to the idea that this integrative measurement can be used as a proxy to assess chronic stress in birds. We also show that birds living in the proximity of airports do not exhibit consistent elevations of CORT_f_ in their feathers, but that species with low frequency songs seems be more affected than those with higher song frequency. Although extreme noise seems to be the factor driving such responses, it is important to notice other probable aspects of airport environments that could be affecting CORT_f_ concentration (e.g. air pollution, nearby urbanization, resource availability, etc.). We stress that these results should be taken with caution when considering species that are more sensitive to urban environments, since our analyses only comprise less sensitive species.

## Supplementary Material

Web_Material_coad079
