# Supplementary material for: Birds living near airports do not show consistently higher levels of feather corticosterone
Source: Conserv Physiol. 2023 Oct 18;11(1):coad079. doi: 10.1093/conphys/coad079 (PMC10588694; doi:10.1093/conphys/coad079)

Birds living near airports do not show consistently higher levels of feather corticosterone

Supplementary Data

A. Results from a linear regression between $CORT_f$ concentration based on feather mass and feather length.

B. Raw data. ID: Laboratory sample control number; Species: *Coereba flaveola*, *Coryphospingus cucullatus*, *Cyclarhis gujanensis*, *Elaenia chiriquensis*, *Elaenia cristata*, *Eupetomena macroura*, *Myiarchus swainsoni*, *Pitangus sulphuratus*, *Synallaxis frontalis*, *Thraupis/Tangara sayaca*, *Troglodytes musculus*, *Turdus leucomelas*, *Turdus rufiventris*, *Volatinia jacarina*, and *Zonotrichia capensis*; Body weight (g); Tarsus length (mm); Wing length (mm); Tail length (mm); Body size (PCA1 from tarsus, wing and length log transformed); Body condition index (residuals from linear regression of body weight ~ body size); D_1: dataset 1 for body condition model (N = 308); D_2: dataset 2 for species-specific models (N = 595); D_3: dataset 3 for population model (N = 512).

C. R scripts and outputs for species-specific analysis, including Global Model and each species-specific model.

D. Data used in Population-specific model. Including raw data, R scripts, and outputs.

A. Results from a linear regression between $CORT_f$ concentration based on feather mass and feather length.

Suppl. Table A. Results from a linear regression between $CORT_f$ concentration based on feather mass and feather length (N = 595). Variables were box-cox transformed.

**Model: $CORT_f$ mass ~ $CORT_f$ length**				
	Estimate	SE	t value	p value
(intercept)	4.58^{-16}	0.21	0.00	1.00
$CORT_f$ length	8.51^{-1}	0.21	39.51	<0.001***

Suppl. Figure A. Regression between CORT values based on feather mass and feather length.

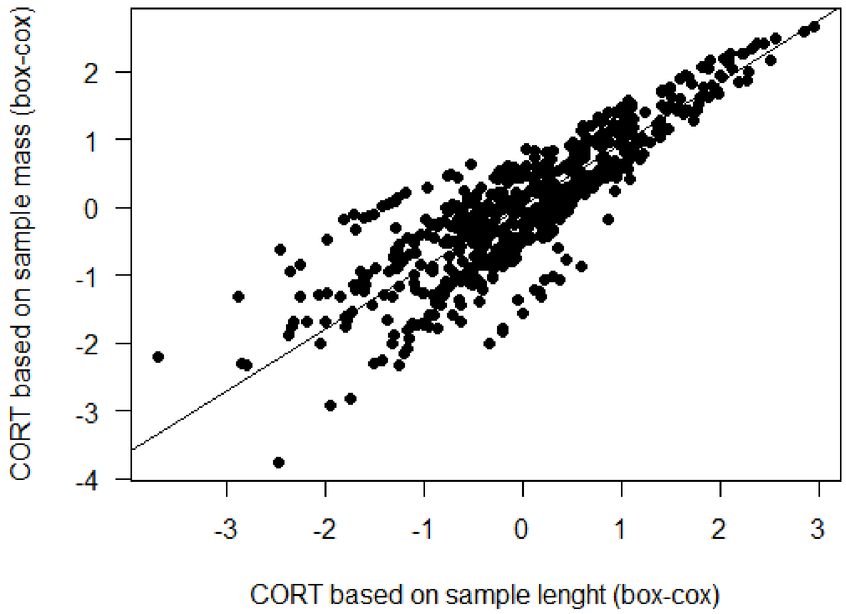

B. Raw data. ID: Laboratory sample control number; Species: *Coereba flaveola*, *Coryphospingus cucullatus*, *Cyclarhis gujanensis*, *Elaenia chiriquensis*, *Elaenia cristata*, *Eupetomena macroura*, *Myiarchus swainsoni*, *Pitangus sulphuratus*, *Synallaxis frontalis*, *Thraupis/Tangara sayaca*, *Troglodytes musculus*, *Turdus leucomelas*, *Turdus rufiventris*, *Volatinia jacarina*, and *Zonotrichia capensis*; Body weight (g); Tarsus length (mm); Wing length (mm); Tail length (mm); Body size (PCA1 from tarsus, wing and length log transformed); Body condition index (residuals from linear regression of body weight ~ body size); D_1: dataset 1 for body condition model (N = 308); D_2: dataset 2 for species-specific models (N = 595); D_3: dataset 3 for population model (N = 512).

ID	Species	Plate	$CORT_f$ (pg mg^{-1})	$CORT_f$ (pg mm^{-1})	Site type	Region	Body weight	Tarsus	Wing	Tail	Body size	Body condition	D_1	D_2	D_3
497	*C. flaveola*	15	28.333	1.635	AIR	SAL	-	-	-	-	-	-	No	Yes	Yes
498	*C. flaveola*	15	24.144	1.515	AIR	SAL	-	-	-	-	-	-	No	Yes	Yes
499	*C. flaveola*	15	11.264	0.765	AIR	SAL	-	-	-	-	-	-	No	Yes	Yes
500	*C. flaveola*	15	34.846	2.136	AIR	SAL	-	-	-	-	-	-	No	Yes	Yes
501	*C. flaveola*	15	32.178	1.978	AIR	SAL	-	-	-	-	-	-	No	Yes	Yes
502	*C. flaveola*	15	22.364	1.234	AIR	SAL	-	-	-	-	-	-	No	Yes	Yes
503	*C. flaveola*	15	16.023	1.035	AIR	SAL	-	-	-	-	-	-	No	Yes	Yes
504	*C. flaveola*	15	29.184	1.851	AIR	SAL	-	-	-	-	-	-	No	Yes	Yes
505	*C. flaveola*	16	43.306	2.822	AIR	SAL	-	-	-	-	-	-	No	Yes	Yes
77	*C. flaveola*	2	19.031	1.288	CONT	SAL	-	-	-	-	-	-	No	Yes	Yes
78	*C. flaveola*	2	36.333	2.225	CONT	SAL	-	-	-	-	-	-	No	Yes	Yes
485	*C. flaveola*	15	42.516	2.981	CONT	SAL	-	-	-	-	-	-	No	Yes	Yes
486	*C. flaveola*	15	38.425	2.507	CONT	SAL	-	-	-	-	-	-	No	Yes	Yes
487	*C. flaveola*	15	29.688	1.720	CONT	SAL	-	-	-	-	-	-	No	Yes	Yes
488	*C. flaveola*	15	26.116	1.729	CONT	SAL	-	-	-	-	-	-	No	Yes	Yes
489	*C. flaveola*	15	32.335	2.024	CONT	SAL	-	-	-	-	-	-	No	Yes	Yes
490	*C. flaveola*	15	29.522	1.896	CONT	SAL	-	-	-	-	-	-	No	Yes	Yes
491	*C. flaveola*	15	49.691	3.221	CONT	SAL	-	-	-	-	-	-	No	Yes	Yes
492	*C. flaveola*	15	32.933	2.065	CONT	SAL	-	-	-	-	-	-	No	Yes	Yes
493	*C. flaveola*	15	33.339	2.118	CONT	SAL	-	-	-	-	-	-	No	Yes	Yes
494	*C. flaveola*	15	44.251	2.868	CONT	SAL	-	-	-	-	-	-	No	Yes	Yes
495	*C. flaveola*	15	28.910	1.800	CONT	SAL	-	-	-	-	-	-	No	Yes	Yes
496	*C. flaveola*	15	19.978	1.338	CONT	SAL	-	-	-	-	-	-	No	Yes	Yes
418	*C. cucullatus*	11	27.678	3.523	CONT	BRAS	15.0	21.0	63.0	60.0	-0.004	0.036	Yes	Yes	No
419	*C. cucullatus*	11	48.528	4.613	CONT	BRAS	-	-	-	-	-	-	No	Yes	No
83	*C. cucullatus*	11	15.264	1.880	AIR	CAMP	-	-	-	-	-	-	No	Yes	Yes

ID	Species	Plate	CORT$_f$ (pg/mg)	CORT$_f$ (pg/mm)	Site type	Region	Body weight	Tarsus	Wing	Tail	Body size	Body condition	D$_1$	D$_2$	D$_3$
84	*C. cucullatus*	11	18.611	2.346	AIR	CAMP	-	-	-	-	-	-	No	Yes	Yes
428	*C. cucullatus*	14	55.374	6.625	AIR	CAMP	-	-	-	-	-	-	No	Yes	Yes
429	*C. cucullatus*	14	31.124	4.159	AIR	CAMP	14.0	23.0	64.0	56.0	-0.007	0.008	Yes	Yes	Yes
430	*C. cucullatus*	14	37.356	4.793	AIR	CAMP	13.0	21.0	58.8	54.8	-0.050	0.010	Yes	Yes	Yes
431	*C. cucullatus*	14	40.857	5.565	AIR	CAMP	16.0	23.0	63.0	60.0	0.009	0.053	Yes	Yes	Yes
432	*C. cucullatus*	14	56.749	7.195	AIR	CAMP	13.0	22.0	62.0	56.0	-0.022	-0.012	Yes	Yes	Yes
433	*C. cucullatus*	14	38.634	5.105	AIR	CAMP	13.5	20.9	58.7	56.2	-0.044	0.022	Yes	Yes	Yes
434	*C. cucullatus*	14	32.728	4.168	AIR	CAMP	15.0	23.0	67.0	61.0	0.031	0.008	Yes	Yes	Yes
435	*C. cucullatus*	14	26.666	2.876	AIR	CAMP	16.0	24.0	62.0	56.0	-0.010	0.068	Yes	Yes	Yes
436	*C. cucullatus*	14	31.723	3.874	AIR	CAMP	15.0	23.0	67.0	58.0	0.016	0.020	Yes	Yes	Yes
437	*C. cucullatus*	14	32.728	4.438	AIR	CAMP	16.0	23.0	70.0	65.0	0.063	0.011	Yes	Yes	Yes
438	*C. cucullatus*	14	37.163	4.878	AIR	CAMP	12.0	24.0	65.0	60.0	0.024	-0.083	Yes	Yes	Yes
439	*C. cucullatus*	14	37.687	4.981	AIR	CAMP	14.0	23.0	65.0	64.0	0.037	-0.027	Yes	Yes	Yes
440	*C. cucullatus*	14	29.598	3.786	AIR	CAMP	14.0	21.0	64.0	60.0	0.001	0.002	Yes	Yes	Yes
441	*C. cucullatus*	14	26.868	3.249	AIR	CAMP	16.0	23.0	63.0	60.0	0.009	0.053	Yes	Yes	Yes
442	*C. cucullatus*	14	69.724	8.343	AIR	CAMP	-	-	-	-	-	-	No	Yes	Yes
443	*C. cucullatus*	14	77.474	10.376	AIR	CAMP	12.0	21.5	57.0	57.1	-0.043	-0.030	Yes	Yes	Yes
444	*C. cucullatus*	14	30.908	3.946	AIR	CAMP	15.0	23.0	65.0	61.0	0.023	0.014	Yes	Yes	Yes
445	*C. cucullatus*	14	26.510	2.966	AIR	CAMP	12.0	24.0	65.0	60.0	0.024	-0.083	Yes	Yes	Yes
420	*C. cucullatus*	11	19.163	2.561	CONT	CAMP	17.0	23.0	65.0	64.5	0.040	0.056	Yes	Yes	Yes
421	*C. cucullatus*	11	28.230	3.137	CONT	CAMP	13.0	20.9	66.0	58.5	0.001	-0.030	Yes	Yes	Yes
422	*C. cucullatus*	11	32.773	3.587	CONT	CAMP	15.5	22.0	61.0	56.0	-0.027	0.068	Yes	Yes	Yes
423	*C. cucullatus*	11	24.938	2.522	CONT	CAMP	12.0	24.0	62.0	56.0	-0.010	-0.057	Yes	Yes	Yes
424	*C. cucullatus*	11	21.426	2.275	CONT	CAMP	13.0	22.0	61.0	55.0	-0.032	-0.004	Yes	Yes	Yes
425	*C. cucullatus*	11	18.945	2.595	CONT	CAMP	14.0	20.0	67.0	62.0	0.016	-0.010	Yes	Yes	Yes
426	*C. cucullatus*	11	32.520	4.230	CONT	CAMP	13.0	23.0	62.0	55.0	-0.021	-0.013	Yes	Yes	Yes
427	*C. cucullatus*	14	62.571	8.323	CONT	CAMP	11.0	22.1	60.8	56.3	-0.025	-0.082	Yes	Yes	Yes
89	*C. gujanensis*	2	171.115	26.700	AIR	BRAS	-	-	-	-	-	-	No	Yes	No
90	*C. gujanensis*	2	71.476	10.253	CONT	BRAS	29.0	26.0	75.0	64.0	-0.006	0.043	Yes	Yes	No
463	*C. gujanensis*	15	19.217	2.883	CONT	CAMP	28.0	30.0	78.0	70.0	0.066	0.002	Yes	Yes	No
464	*C. gujanensis*	15	27.995	3.733	CONT	CAMP	26.0	27.0	79.0	70.0	0.039	-0.021	Yes	Yes	No
465	*C. gujanensis*	15	106.599	15.748	CONT	CAMP	27.0	30.0	77.0	70.0	0.064	-0.013	Yes	Yes	No

ID	Species	Plate	CORTf (pg/mg)	CORTf (pg/mm)	Site type	Region	Body weight	Tarsus	Wing	Tail	Body size	Body condition	D_1	D_2	D_3
472	*C. gujanensis*	15	64.738	8.929	AIR	SAL	26.5	25.0	74.0	61.0	-0.033	0.013	Yes	Yes	Yes
473	*C. gujanensis*	15	77.619	9.969	AIR	SAL	-	-	-	-	-	-	No	Yes	Yes
474	*C. gujanensis*	15	50.506	6.795	AIR	SAL	-	-	-	-	-	-	No	Yes	Yes
475	*C. gujanensis*	15	264.694	36.053	AIR	SAL	-	-	-	-	-	-	No	Yes	Yes
476	*C. gujanensis*	15	114.803	16.072	AIR	SAL	24.0	25.0	71.0	60.0	-0.045	-0.026	Yes	Yes	Yes
477	*C. gujanensis*	15	220.205	30.373	AIR	SAL	23.0	25.0	68.0	70.0	-0.008	-0.057	Yes	Yes	Yes
478	*C. gujanensis*	15	17.123	2.160	AIR	SAL	25.0	26.0	68.0	58.0	-0.051	-0.006	Yes	Yes	Yes
479	*C. gujanensis*	15	63.592	9.628	AIR	SAL	26.0	25.0	70.0	57.0	-0.062	0.015	Yes	Yes	Yes
480	*C. gujanensis*	15	64.168	8.823	AIR	SAL	-	-	-	-	-	-	No	Yes	Yes
466	*C. gujanensis*	15	30.439	4.457	CONT	SAL	27.0	29.0	73.0	61.0	0.006	0.007	Yes	Yes	Yes
467	*C. gujanensis*	15	34.061	4.573	CONT	SAL	26.5	29.0	72.0	61.0	0.003	0.000	Yes	Yes	Yes
468	*C. gujanensis*	15	31.476	4.413	CONT	SAL	25.0	27.0	71.0	60.0	-0.023	-0.016	Yes	Yes	Yes
469	*C. gujanensis*	15	72.664	10.670	CONT	SAL	28.5	30.0	74.0	62.0	0.022	0.025	Yes	Yes	Yes
470	*C. gujanensis*	15	16.055	2.082	CONT	SAL	28.0	28.0	74.0	62.0	0.003	0.024	Yes	Yes	Yes
471	*C. gujanensis*	15	45.801	5.850	CONT	SAL	27.5	28.0	73.0	67.0	0.023	0.009	Yes	Yes	Yes
193	*E. chiriquensis*	3	40.315	4.912	AIR	BRAS	-	-	-	-	-	-	No	Yes	Yes
194	*E. chiriquensis*	3	30.447	3.476	AIR	BRAS	-	-	-	-	-	-	No	Yes	Yes
195	*E. chiriquensis*	3	62.231	6.937	AIR	BRAS	-	-	-	-	-	-	No	Yes	Yes
196	*E. chiriquensis*	3	57.463	6.802	AIR	BRAS	-	-	-	-	-	-	No	Yes	Yes
197	*E. chiriquensis*	3	44.202	5.265	AIR	BRAS	-	-	-	-	-	-	No	Yes	Yes
198	*E. chiriquensis*	3	49.704	5.262	AIR	BRAS	-	-	-	-	-	-	No	Yes	Yes
199	*E. chiriquensis*	3	32.466	4.184	AIR	BRAS	-	-	-	-	-	-	No	Yes	Yes
201	*E. chiriquensis*	3	36.654	4.351	AIR	BRAS	-	-	-	-	-	-	No	Yes	Yes
202	*E. chiriquensis*	3	63.495	6.499	AIR	BRAS	-	-	-	-	-	-	No	Yes	Yes
203	*E. chiriquensis*	3	31.472	4.446	AIR	BRAS	-	-	-	-	-	-	No	Yes	Yes
204	*E. chiriquensis*	3	62.003	7.168	AIR	BRAS	-	-	-	-	-	-	No	Yes	Yes
205	*E. chiriquensis*	3	44.482	5.307	AIR	BRAS	-	-	-	-	-	-	No	Yes	Yes
206	*E. chiriquensis*	3	47.970	5.422	AIR	BRAS	-	-	-	-	-	-	No	Yes	Yes
207	*E. chiriquensis*	3	36.318	3.852	AIR	BRAS	-	-	-	-	-	-	No	Yes	Yes
208	*E. chiriquensis*	3	45.923	5.186	AIR	BRAS	-	-	-	-	-	-	No	Yes	Yes
209	*E. chiriquensis*	3	45.813	5.271	AIR	BRAS	-	-	-	-	-	-	No	Yes	Yes
210	*E. chiriquensis*	3	66.434	8.185	AIR	BRAS	-	-	-	-	-	-	No	Yes	Yes

ID	Species	Plate	CORTf (pg/mg)	CORTf (pg/mm)	Site type	Region	Body weight	Tarsus	Wing	Tail	Body size	Body condition	D_1	D_2	D_3
211	*E. chiriquensis*	3	43.440	4.698	AIR	BRAS	-	-	-	-	-	-	No	Yes	Yes
212	*E. chiriquensis*	3	180.644	20.755	AIR	BRAS	-	-	-	-	-	-	No	Yes	Yes
213	*E. chiriquensis*	4	52.407	5.581	AIR	BRAS	-	-	-	-	-	-	No	Yes	Yes
214	*E. chiriquensis*	4	25.556	3.106	AIR	BRAS	-	-	-	-	-	-	No	Yes	Yes
215	*E. chiriquensis*	4	34.055	3.174	AIR	BRAS	-	-	-	-	-	-	No	Yes	Yes
81	*E. chiriquensis*	17	18.446	1.829	CONT	BRAS	-	-	-	-	-	-	No	Yes	Yes
82	*E. chiriquensis*	3	60.184	7.207	CONT	BRAS	-	-	-	-	-	-	No	Yes	Yes
216	*E. chiriquensis*	4	13.847	1.669	CONT	BRAS	-	-	-	-	-	-	No	Yes	Yes
217	*E. chiriquensis*	4	14.608	1.705	CONT	BRAS	-	-	-	-	-	-	No	Yes	Yes
218	*E. chiriquensis*	4	28.270	2.979	CONT	BRAS	-	-	-	-	-	-	No	Yes	Yes
219	*E. chiriquensis*	4	24.116	2.820	CONT	BRAS	-	-	-	-	-	-	No	Yes	Yes
220	*E. chiriquensis*	4	45.496	5.165	CONT	BRAS	-	-	-	-	-	-	No	Yes	Yes
221	*E. chiriquensis*	4	14.152	1.656	CONT	BRAS	-	-	-	-	-	-	No	Yes	Yes
222	*E. chiriquensis*	4	23.730	2.943	CONT	BRAS	-	-	-	-	-	-	No	Yes	Yes
223	*E. chiriquensis*	4	21.704	2.346	CONT	BRAS	-	-	-	-	-	-	No	Yes	Yes
224	*E. chiriquensis*	4	26.412	3.201	CONT	BRAS	-	-	-	-	-	-	No	Yes	Yes
225	*E. chiriquensis*	4	21.693	2.353	CONT	BRAS	-	-	-	-	-	-	No	Yes	Yes
226	*E. chiriquensis*	4	13.384	1.655	CONT	BRAS	-	-	-	-	-	-	No	Yes	Yes
227	*E. chiriquensis*	4	26.346	3.001	CONT	BRAS	-	-	-	-	-	-	No	Yes	Yes
228	*E. chiriquensis*	4	12.136	1.467	CONT	BRAS	-	-	-	-	-	-	No	Yes	Yes
229	*E. chiriquensis*	4	24.503	3.049	CONT	BRAS	-	-	-	-	-	-	No	Yes	Yes
230	*E. chiriquensis*	4	34.454	3.833	CONT	BRAS	-	-	-	-	-	-	No	Yes	Yes
231	*E. chiriquensis*	4	30.144	3.547	CONT	BRAS	-	-	-	-	-	-	No	Yes	Yes
232	*E. chiriquensis*	4	34.642	4.446	CONT	BRAS	-	-	-	-	-	-	No	Yes	Yes
233	*E. chiriquensis*	5	78.003	9.084	CONT	BRAS	-	-	-	-	-	-	No	Yes	Yes
234	*E. chiriquensis*	5	85.606	10.359	CONT	BRAS	-	-	-	-	-	-	No	Yes	Yes
235	*E. chiriquensis*	5	34.491	3.978	CONT	BRAS	-	-	-	-	-	-	No	Yes	Yes
236	*E. chiriquensis*	5	23.333	2.756	CONT	BRAS	-	-	-	-	-	-	No	Yes	Yes
237	*E. chiriquensis*	5	31.575	3.668	CONT	BRAS	-	-	-	-	-	-	No	Yes	Yes
238	*E. chiriquensis*	5	33.882	3.904	CONT	BRAS	-	-	-	-	-	-	No	Yes	Yes
239	*E. chiriquensis*	5	47.414	5.334	CONT	BRAS	-	-	-	-	-	-	No	Yes	Yes
240	*E. chiriquensis*	5	25.675	3.223	CONT	BRAS	-	-	-	-	-	-	No	Yes	Yes

ID	Species	Plate	$CORT_f$ (pg/mg)	$CORT_f$ (pg/mm)	Site type	Region	Body weight	Tarsus	Wing	Tail	Body size	Body condition	D_1	D_2	D_3
241	*E. chiriquensis*	5	29.024	3.396	CONT	BRAS	-	-	-	-	-	-	No	Yes	Yes
242	*E. chiriquensis*	5	35.019	3.881	CONT	BRAS	-	-	-	-	-	-	No	Yes	Yes
243	*E. chiriquensis*	5	44.397	4.992	CONT	BRAS	-	-	-	-	-	-	No	Yes	Yes
244	*E. chiriquensis*	5	44.418	5.570	CONT	BRAS	-	-	-	-	-	-	No	Yes	Yes
245	*E. chiriquensis*	5	27.878	3.395	CONT	BRAS	-	-	-	-	-	-	No	Yes	Yes
246	*E. chiriquensis*	5	76.371	8.910	CONT	BRAS	-	-	-	-	-	-	No	Yes	Yes
247	*E. chiriquensis*	5	27.108	3.120	CONT	BRAS	-	-	-	-	-	-	No	Yes	Yes
248	*E. chiriquensis*	5	64.130	7.249	CONT	BRAS	-	-	-	-	-	-	No	Yes	Yes
249	*E. chiriquensis*	5	19.833	2.413	CONT	BRAS	-	-	-	-	-	-	No	Yes	Yes
250	*E. chiriquensis*	5	26.477	3.024	CONT	BRAS	-	-	-	-	-	-	No	Yes	Yes
70	*E. cristata*	9	22.919	3.052	AIR	BRAS	-	-	-	-	-	-	No	Yes	Yes
71	*E. cristata*	9	32.969	4.149	AIR	BRAS	-	-	-	-	-	-	No	Yes	Yes
364	*E. cristata*	11	38.831	5.760	AIR	BRAS	17.0	21.9	70.0	66.0	0.011	-0.045	Yes	Yes	Yes
365	*E. cristata*	11	48.236	6.174	AIR	BRAS	19.0	21.3	66.2	61.5	-0.030	0.035	Yes	Yes	Yes
366	*E. cristata*	11	81.723	9.594	AIR	BRAS	-	-	-	-	-	-	No	Yes	Yes
367	*E. cristata*	11	43.826	6.992	AIR	BRAS	21.0	23.0	76.0	69.0	0.054	0.013	Yes	Yes	Yes
368	*E. cristata*	11	52.685	8.255	AIR	BRAS	20.0	22.5	71.3	63.2	0.008	0.027	Yes	Yes	Yes
369	*E. cristata*	11	25.376	3.620	AIR	BRAS	17.0	20.0	79.0	65.0	0.019	-0.051	Yes	Yes	Yes
370	*E. cristata*	11	73.161	9.602	AIR	BRAS	17.0	21.9	72.0	63.1	0.005	-0.040	Yes	Yes	Yes
371	*E. cristata*	11	37.589	4.932	AIR	BRAS	17.0	18.5	68.9	64.3	-0.035	-0.009	Yes	Yes	Yes
372	*E. cristata*	11	39.900	5.064	AIR	BRAS	20.0	24.0	76.0	69.0	0.063	-0.015	Yes	Yes	Yes
373	*E. cristata*	11	51.745	6.554	AIR	BRAS	18.5	21.6	68.0	63.4	-0.011	0.009	Yes	Yes	Yes
374	*E. cristata*	11	16.737	2.361	AIR	BRAS	21.5	22.6	75.8	68.2	0.047	0.029	Yes	Yes	Yes
375	*E. cristata*	11	13.899	2.025	AIR	BRAS	20.0	22.0	75.0	70.0	0.046	-0.002	Yes	Yes	Yes
376	*E. cristata*	11	25.216	3.100	AIR	BRAS	16.0	20.0	72.0	68.0	0.008	-0.069	Yes	Yes	Yes
377	*E. cristata*	11	114.850	14.670	AIR	BRAS	19.0	21.0	69.0	64.0	-0.010	0.020	Yes	Yes	Yes
378	*E. cristata*	11	60.348	8.298	CONT	BRAS	19.0	21.0	69.5	65.5	-0.002	0.013	Yes	Yes	Yes
379	*E. cristata*	11	25.438	3.716	CONT	BRAS	18.0	22.0	72.0	61.0	-0.004	-0.009	Yes	Yes	Yes
380	*E. cristata*	11	68.498	7.925	CONT	BRAS	19.0	21.0	73.0	60.0	-0.015	0.023	Yes	Yes	Yes
381	*E. cristata*	11	31.855	3.364	CONT	BRAS	-	-	-	-	-	-	No	Yes	Yes
382	*E. cristata*	11	38.579	5.144	CONT	BRAS	20.0	22.5	77.0	66.0	0.040	0.002	Yes	Yes	Yes
383	*E. cristata*	11	136.196	16.633	CONT	BRAS	18.0	20.8	66.5	57.5	-0.053	0.029	Yes	Yes	Yes

ID	Species	Plate	CORTf (pg/mg)	CORTf (pg/mm)	Site type	Region	Body weight	Tarsus	Wing	Tail	Body size	Body condition	D_1	D_2	D_3
384	*E. cristata*	11	72.501	8.356	CONT	BRAS	16.5	20.4	66.1	57.3	-0.059	-0.004	Yes	Yes	Yes
385	*E. cristata*	11	30.126	3.339	CONT	BRAS	18.0	21.0	70.5	62.0	-0.014	-0.001	Yes	Yes	Yes
386	*E. cristata*	11	33.792	4.376	CONT	BRAS	18.5	22.1	68.6	61.6	-0.013	0.010	Yes	Yes	Yes
387	*E. cristata*	11	69.157	8.136	CONT	BRAS	16.0	20.8	68.0	61.2	-0.029	-0.040	Yes	Yes	Yes
388	*E. cristata*	11	51.410	6.465	CONT	BRAS	20.5	23.2	72.9	66.1	0.033	0.019	Yes	Yes	Yes
389	*E. cristata*	11	26.012	3.427	CONT	BRAS	20.0	23.4	73.5	63.8	0.027	0.013	Yes	Yes	Yes
390	*E. cristata*	11	79.877	11.144	CONT	BRAS	16.0	21.2	66.4	60.8	-0.033	-0.037	Yes	Yes	Yes
391	*E. cristata*	11	63.335	7.979	CONT	BRAS	20.0	22.8	69.4	63.8	0.007	0.029	Yes	Yes	Yes
392	*E. cristata*	11	128.461	18.320	CONT	BRAS	17.0	21.6	66.3	57.4	-0.046	-0.001	Yes	Yes	Yes
393	*E. cristata*	11	48.810	6.630	CONT	BRAS	18.0	21.5	68.3	56.6	-0.044	0.022	Yes	Yes	Yes
394	*E. cristata*	11	53.136	7.179	CONT	BRAS	21.0	22.9	75.4	64.4	0.031	0.031	Yes	Yes	Yes
454	*E. macroura*	12	17.617	3.448	AIR	SAL	-	-	-	-	-	-	No	Yes	Yes
455	*E. macroura*	12	20.277	3.074	AIR	SAL	-	-	-	-	-	-	No	Yes	Yes
456	*E. macroura*	12	15.478	3.409	AIR	SAL	-	-	-	-	-	-	No	Yes	Yes
457	*E. macroura*	12	18.320	3.708	AIR	SAL	-	-	-	-	-	-	No	Yes	Yes
458	*E. macroura*	12	19.357	3.209	AIR	SAL	-	-	-	-	-	-	No	Yes	Yes
459	*E. macroura*	12	22.071	3.936	AIR	SAL	-	-	-	-	-	-	No	Yes	Yes
460	*E. macroura*	12	16.368	2.469	AIR	SAL	-	-	-	-	-	-	No	Yes	Yes
461	*E. macroura*	12	16.416	2.454	AIR	SAL	-	-	-	-	-	-	No	Yes	Yes
462	*E. macroura*	12	19.654	2.585	AIR	SAL	-	-	-	-	-	-	No	Yes	Yes
446	*E. macroura*	12	36.815	4.992	CONT	SAL	-	-	-	-	-	-	No	Yes	Yes
447	*E. macroura*	12	17.459	2.341	CONT	SAL	-	-	-	-	-	-	No	Yes	Yes
448	*E. macroura*	12	23.095	4.337	CONT	SAL	-	-	-	-	-	-	No	Yes	Yes
449	*E. macroura*	12	23.044	3.572	CONT	SAL	-	-	-	-	-	-	No	Yes	Yes
450	*E. macroura*	12	17.640	2.778	CONT	SAL	-	-	-	-	-	-	No	Yes	Yes
451	*E. macroura*	12	23.497	3.286	CONT	SAL	-	-	-	-	-	-	No	Yes	Yes
452	*E. macroura*	12	22.034	3.011	CONT	SAL	-	-	-	-	-	-	No	Yes	Yes
453	*E. macroura*	12	30.472	3.506	CONT	SAL	-	-	-	-	-	-	No	Yes	Yes
185	*M. swainsoni*	6	28.919	5.093	AIR	BRAS	24.0	23.7	86.4	81.8	0.001	0.004	Yes	Yes	Yes
186	*M. swainsoni*	6	22.120	3.784	AIR	BRAS	24.0	19.0	93.0	81.0	-0.090	0.020	Yes	Yes	Yes
187	*M. swainsoni*	6	26.760	5.155	AIR	BRAS	23.5	18.5	96.0	77.0	-0.111	0.015	Yes	Yes	Yes
188	*M. swainsoni*	6	42.024	6.724	AIR	BRAS	25.0	25.0	89.0	82.0	0.021	0.019	Yes	Yes	Yes

ID	Species	Plate	$CORT_f$ (pg/mg)	$CORT_f$ (pg/mm)	Site type	Region	Body weight	Tarsus	Wing	Tail	Body size	Body condition	D_1	D_2	D_3
189	*M. swainsoni*	6	38.855	7.291	AIR	BRAS	25.0	23.4	92.8	87.6	0.006	0.021	Yes	Yes	Yes
190	*M. swainsoni*	6	36.849	6.274	AIR	BRAS	27.0	23.0	89.0	85.0	-0.005	0.057	Yes	Yes	Yes
191	*M. swainsoni*	6	40.613	7.479	AIR	BRAS	24.0	25.5	92.5	88.8	0.042	-0.003	Yes	Yes	Yes
192	*M. swainsoni*	6	73.693	13.946	AIR	BRAS	23.0	21.0	95.0	85.0	-0.043	-0.007	Yes	Yes	Yes
168	*M. swainsoni*	6	49.640	8.839	CONT	BRAS	27.0	23.3	84.2	81.8	-0.005	0.057	Yes	Yes	Yes
169	*M. swainsoni*	6	136.485	23.043	CONT	BRAS	21.0	22.0	86.0	80.0	-0.032	-0.048	Yes	Yes	Yes
170	*M. swainsoni*	6	29.563	5.187	CONT	BRAS	28.0	25.0	88.0	85.0	0.028	0.067	Yes	Yes	Yes
171	*M. swainsoni*	6	31.751	6.071	CONT	BRAS	24.0	25.0	85.0	88.0	0.036	-0.002	Yes	Yes	Yes
172	*M. swainsoni*	6	44.224	7.135	CONT	BRAS	23.0	24.0	89.0	80.0	0.001	-0.014	Yes	Yes	Yes
173	*M. swainsoni*	6	32.770	5.870	CONT	BRAS	25.5	22.9	91.9	82.1	-0.014	0.033	Yes	Yes	Yes
174	*M. swainsoni*	6	32.657	5.457	CONT	BRAS	22.0	23.0	85.5	80.1	-0.014	-0.031	Yes	Yes	Yes
175	*M. swainsoni*	6	70.906	11.136	CONT	BRAS	20.5	22.9	82.8	76.6	-0.023	-0.060	Yes	Yes	Yes
176	*M. swainsoni*	6	34.661	5.921	CONT	BRAS	22.5	23.0	95.0	89.0	0.001	-0.024	Yes	Yes	Yes
177	*M. swainsoni*	6	31.568	5.460	CONT	BRAS	23.0	23.3	86.6	86.6	0.005	-0.015	Yes	Yes	Yes
178	*M. swainsoni*	6	18.645	2.945	CONT	BRAS	26.0	22.6	86.5	82.1	-0.017	0.042	Yes	Yes	Yes
179	*M. swainsoni*	6	48.452	9.476	CONT	BRAS	25.5	23.0	95.0	90.0	0.003	0.030	Yes	Yes	Yes
180	*M. swainsoni*	6	106.187	19.952	CONT	BRAS	23.0	20.0	95.0	80.0	-0.073	-0.001	Yes	Yes	Yes
181	*M. swainsoni*	6	42.580	7.260	CONT	BRAS	24.0	25.0	90.0	85.0	0.028	0.000	Yes	Yes	Yes
182	*M. swainsoni*	6	65.762	12.486	CONT	BRAS	21.0	22.0	85.0	80.0	-0.032	-0.048	Yes	Yes	Yes
183	*M. swainsoni*	6	24.237	4.336	CONT	BRAS	25.0	23.0	85.0	88.0	0.003	0.022	Yes	Yes	Yes
184	*M. swainsoni*	6	38.537	6.699	CONT	BRAS	26.0	23.0	89.0	83.0	-0.009	0.041	Yes	Yes	Yes
166	*M. swainsoni*	6	38.318	7.736	AIR	CAMP	25.0	26.0	91.0	92.0	0.057	0.012	Yes	Yes	No
167	*M. swainsoni*	6	32.956	5.861	AIR	CAMP	26.0	27.0	88.0	85.0	0.058	0.029	Yes	Yes	No
160	*M. swainsoni*	6	20.325	3.503	CONT	CAMP	22.0	23.0	95.0	87.0	-0.003	-0.033	Yes	Yes	No
161	*M. swainsoni*	6	41.783	7.292	CONT	CAMP	21.0	23.0	91.0	90.0	0.005	-0.054	Yes	Yes	No
162	*M. swainsoni*	6	64.487	11.862	CONT	CAMP	23.0	26.0	92.0	100.0	0.072	-0.026	Yes	Yes	No
163	*M. swainsoni*	6	57.339	10.190	CONT	CAMP	25.0	26.0	91.0	85.0	0.042	0.015	Yes	Yes	No
164	*M. swainsoni*	6	27.416	4.670	CONT	CAMP	20.0	25.0	94.0	80.0	0.015	-0.077	Yes	Yes	No
251	*P. sulphuratus*	8	56.007	12.166	AIR	BRAS	52.0	30.0	113.0	85.0	0.016	-0.003	Yes	Yes	No
252	*P. sulphuratus*	8	40.394	7.478	AIR	BRAS	55.0	29.0	110.0	94.0	0.003	0.030	Yes	Yes	No
253	*P. sulphuratus*	8	31.026	5.895	AIR	BRAS	54.0	30.0	110.0	89.0	0.012	0.016	Yes	Yes	No
254	*P. sulphuratus*	8	34.441	5.683	AIR	BRAS	-	-	-	-	-	-	No	Yes	No

ID	Species	Plate	CORTf (pg/mg)	CORTf (pg/mm)	Site type	Region	Body weight	Tarsus	Wing	Tail	Body size	Body condition	D_1	D_2	D_3
255	*P. sulphuratus*	8	40.843	7.403	AIR	BRAS	-	-	-	-	-	-	No	Yes	No
256	*P. sulphuratus*	8	46.481	8.266	AIR	BRAS	-	-	-	-	-	-	No	Yes	No
257	*P. sulphuratus*	8	119.299	22.854	AIR	CAMP	59.0	29.9	107.9	85.9	0.004	0.059	Yes	Yes	No
258	*P. sulphuratus*	8	43.270	7.428	AIR	CAMP	59.0	32.0	120.0	97.0	0.061	0.024	Yes	Yes	No
259	*P. sulphuratus*	8	78.980	15.270	AIR	CAMP	53.0	32.0	112.0	92.0	0.042	-0.010	Yes	Yes	No
260	*P. sulphuratus*	8	105.737	19.611	AIR	CAMP	54.0	28.0	115.0	95.0	0.002	0.022	Yes	Yes	No
261	*P. sulphuratus*	8	34.630	6.629	AIR	CAMP	55.0	31.0	114.0	88.0	0.032	0.012	Yes	Yes	No
262	*P. sulphuratus*	8	36.576	6.005	AIR	CAMP	55.0	32.0	121.0	100.0	0.065	-0.009	Yes	Yes	No
263	*P. sulphuratus*	8	24.295	4.787	AIR	CAMP	-	-	-	-	-	-	No	Yes	No
79	*P. sulphuratus*	8	22.466	5.824	AIR	SAL	-	-	-	-	-	-	No	Yes	Yes
80	*P. sulphuratus*	8	41.691	10.702	AIR	SAL	-	-	-	-	-	-	No	Yes	Yes
274	*P. sulphuratus*	8	30.746	5.417	AIR	SAL	56.0	29.0	118.0	90.0	0.018	0.028	Yes	Yes	Yes
275	*P. sulphuratus*	8	42.711	8.648	AIR	SAL	51.0	28.0	110.0	90.0	-0.012	0.006	Yes	Yes	Yes
276	*P. sulphuratus*	8	52.176	10.823	AIR	SAL	-	-	-	-	-	-	No	Yes	Yes
277	*P. sulphuratus*	8	25.948	5.604	AIR	SAL	-	-	-	-	-	-	No	Yes	Yes
278	*P. sulphuratus*	8	24.207	4.748	AIR	SAL	-	-	-	-	-	-	No	Yes	Yes
279	*P. sulphuratus*	8	106.691	22.164	AIR	SAL	-	-	-	-	-	-	No	Yes	Yes
280	*P. sulphuratus*	8	45.189	8.894	AIR	SAL	48.0	28.0	112.0	85.0	-0.010	-0.021	Yes	Yes	Yes
281	*P. sulphuratus*	8	25.745	5.214	AIR	SAL	-	-	-	-	-	-	No	Yes	Yes
282	*P. sulphuratus*	8	64.146	12.991	AIR	SAL	-	-	-	-	-	-	No	Yes	Yes
283	*P. sulphuratus*	8	78.035	15.966	AIR	SAL	-	-	-	-	-	-	No	Yes	Yes
284	*P. sulphuratus*	8	36.758	9.322	AIR	SAL	-	-	-	-	-	-	No	Yes	Yes
285	*P. sulphuratus*	8	22.114	4.337	AIR	SAL	-	-	-	-	-	-	No	Yes	Yes
286	*P. sulphuratus*	8	243.123	45.889	AIR	SAL	-	-	-	-	-	-	No	Yes	Yes
287	*P. sulphuratus*	8	145.115	29.214	AIR	SAL	-	-	-	-	-	-	No	Yes	Yes
288	*P. sulphuratus*	8	98.935	18.714	AIR	SAL	-	-	-	-	-	-	No	Yes	Yes
289	*P. sulphuratus*	8	134.489	25.691	AIR	SAL	-	-	-	-	-	-	No	Yes	Yes
290	*P. sulphuratus*	10	152.904	28.892	AIR	SAL	-	-	-	-	-	-	No	Yes	Yes
291	*P. sulphuratus*	10	332.909	67.404	AIR	SAL	-	-	-	-	-	-	No	Yes	Yes
292	*P. sulphuratus*	10	183.514	39.517	AIR	SAL	-	-	-	-	-	-	No	Yes	Yes
293	*P. sulphuratus*	10	195.953	45.479	AIR	SAL	41.5	27.0	112.0	90.0	-0.020	-0.078	Yes	Yes	Yes
294	*P. sulphuratus*	10	25.551	5.212	AIR	SAL	51.0	28.0	108.0	82.0	-0.021	0.012	Yes	Yes	Yes

ID	Species	Plate	$CORT_f$ (pg/mg)	$CORT_f$ (pg/mm)	Site type	Region	Body weight	Tarsus	Wing	Tail	Body size	Body condition	D_1	D_2	D_3
295	*P. sulphuratus*	10	20.506	4.234	AIR	SAL	52.5	30.0	110.0	85.0	0.010	0.005	Yes	Yes	Yes
296	*P. sulphuratus*	10	69.934	14.532	AIR	SAL	51.0	29.0	117.0	85.0	0.013	-0.009	Yes	Yes	Yes
297	*P. sulphuratus*	10	25.835	5.654	AIR	SAL	53.5	29.0	110.0	86.0	-0.002	0.021	Yes	Yes	Yes
298	*P. sulphuratus*	10	27.351	5.218	AIR	SAL	51.0	26.0	114.0	94.0	-0.027	0.015	Yes	Yes	Yes
299	*P. sulphuratus*	10	26.519	5.237	AIR	SAL	46.5	27.0	109.0	86.0	-0.029	-0.023	Yes	Yes	Yes
300	*P. sulphuratus*	10	49.780	10.054	AIR	SAL	42.5	26.0	101.0	85.0	-0.062	-0.042	Yes	Yes	Yes
301	*P. sulphuratus*	10	20.356	3.995	AIR	SAL	52.0	30.0	110.0	90.0	0.013	-0.001	Yes	Yes	Yes
302	*P. sulphuratus*	10	17.391	3.258	AIR	SAL	54.5	28.0	110.0	89.0	-0.012	0.035	Yes	Yes	Yes
303	*P. sulphuratus*	10	36.732	7.297	AIR	SAL	49.0	28.0	84.0	107.0	-0.067	0.023	Yes	Yes	Yes
304	*P. sulphuratus*	10	46.503	10.530	AIR	SAL	49.0	26.0	109.0	95.0	-0.037	0.005	Yes	Yes	Yes
305	*P. sulphuratus*	10	18.165	3.716	AIR	SAL	-	-	-	-	-	-	No	Yes	Yes
306	*P. sulphuratus*	10	19.558	3.974	AIR	SAL	-	-	-	-	-	-	No	Yes	Yes
307	*P. sulphuratus*	10	29.313	5.730	AIR	SAL	-	-	-	-	-	-	No	Yes	Yes
308	*P. sulphuratus*	10	21.025	4.343	AIR	SAL	-	-	-	-	-	-	No	Yes	Yes
309	*P. sulphuratus*	10	15.701	3.025	AIR	SAL	-	-	-	-	-	-	No	Yes	Yes
310	*P. sulphuratus*	10	29.236	5.829	AIR	SAL	-	-	-	-	-	-	No	Yes	Yes
311	*P. sulphuratus*	10	20.027	3.981	AIR	SAL	56.0	29.0	114.0	90.0	0.010	0.033	Yes	Yes	Yes
312	*P. sulphuratus*	10	24.559	5.270	AIR	SAL	47.0	28.0	105.0	82.0	-0.028	-0.019	Yes	Yes	Yes
264	*P. sulphuratus*	8	28.251	5.580	CONT	SAL	49.5	25.0	114.0	90.0	-0.043	0.013	Yes	Yes	Yes
265	*P. sulphuratus*	8	56.965	11.286	CONT	SAL	53.5	30.0	109.0	73.0	-0.001	0.020	Yes	Yes	Yes
266	*P. sulphuratus*	8	30.510	6.334	CONT	SAL	45.5	33.0	115.0	89.0	0.057	-0.086	Yes	Yes	Yes
267	*P. sulphuratus*	8	30.079	6.109	CONT	SAL	52.0	31.0	115.0	99.0	0.041	-0.018	Yes	Yes	Yes
268	*P. sulphuratus*	8	28.474	5.920	CONT	SAL	49.0	30.0	109.0	85.0	0.007	-0.023	Yes	Yes	Yes
269	*P. sulphuratus*	8	35.920	7.184	CONT	SAL	57.5	30.0	111.0	87.0	0.013	0.043	Yes	Yes	Yes
270	*P. sulphuratus*	8	109.046	22.254	CONT	SAL	46.0	26.0	106.0	85.0	-0.050	-0.015	Yes	Yes	Yes
271	*P. sulphuratus*	8	42.863	9.502	CONT	SAL	44.5	30.0	104.0	81.0	-0.007	-0.056	Yes	Yes	Yes
272	*P. sulphuratus*	8	46.367	8.921	CONT	SAL	50.5	31.0	104.0	85.0	0.008	-0.010	Yes	Yes	Yes
273	*P. sulphuratus*	8	40.784	8.032	CONT	SAL	-	-	-	-	-	-	No	Yes	Yes
591	*S. frontalis*	16	106.456	17.452	AIR	BRAS	14.0	23.0	59.0	79.0	0.013	-0.014	Yes	Yes	No
592	*S. frontalis*	16	37.051	5.401	AIR	BRAS	15.0	24.0	58.0	86.0	0.046	0.000	Yes	Yes	No
593	*S. frontalis*	16	51.060	9.391	AIR	BRAS	14.0	23.0	59.0	86.0	0.045	-0.029	Yes	Yes	No
594	*S. frontalis*	16	18.100	3.345	CONT	BRAS	-	-	-	-	-	-	No	Yes	No

ID	Species	Plate	$CORT_f$ (pg/mg)	$CORT_f$ (pg/mm)	Site type	Region	Body weight	Tarsus	Wing	Tail	Body size	Body condition	D_1	D_2	D_3
595	*S. frontalis*	16	20.108	3.363	CONT	BRAS	-	-	-	-	-	-	No	Yes	No
576	*S. frontalis*	16	109.198	17.675	AIR	CAMP	15.0	23.6	54.0	66.8	-0.065	0.054	Yes	Yes	Yes
577	*S. frontalis*	16	37.230	5.389	AIR	CAMP	12.0	24.0	52.0	75.0	-0.028	-0.061	Yes	Yes	Yes
578	*S. frontalis*	16	64.902	10.119	AIR	CAMP	14.0	25.0	56.0	76.0	-0.004	-0.005	Yes	Yes	Yes
579	*S. frontalis*	16	26.409	4.002	AIR	CAMP	15.5	22.5	59.0	81.0	0.021	0.027	Yes	Yes	Yes
580	*S. frontalis*	16	89.308	14.627	AIR	CAMP	14.0	26.0	55.0	73.0	-0.020	0.002	Yes	Yes	Yes
581	*S. frontalis*	16	21.761	3.377	AIR	CAMP	14.0	25.0	57.0	68.0	-0.043	0.014	Yes	Yes	Yes
582	*S. frontalis*	16	19.327	2.843	AIR	CAMP	17.0	26.0	59.0	80.0	0.029	0.063	Yes	Yes	Yes
583	*S. frontalis*	16	27.122	4.484	AIR	CAMP	14.0	24.0	58.0	83.0	0.032	-0.023	Yes	Yes	Yes
584	*S. frontalis*	16	72.352	10.937	AIR	CAMP	14.0	25.0	57.0	78.0	0.009	-0.012	Yes	Yes	Yes
585	*S. frontalis*	16	22.554	3.457	AIR	CAMP	-	-	-	-	-	-	No	Yes	Yes
586	*S. frontalis*	16	85.951	14.255	AIR	CAMP	15.0	25.0	57.0	79.0	0.014	0.016	Yes	Yes	Yes
587	*S. frontalis*	16	43.143	7.115	AIR	CAMP	15.0	25.0	60.0	80.0	0.029	0.009	Yes	Yes	Yes
588	*S. frontalis*	16	23.093	3.472	AIR	CAMP	-	-	-	-	-	-	No	Yes	Yes
589	*S. frontalis*	16	40.372	6.640	AIR	CAMP	14.0	24.6	56.8	89.4	0.058	-0.036	Yes	Yes	Yes
590	*S. frontalis*	16	24.173	4.002	AIR	CAMP	-	-	-	-	-	-	No	Yes	Yes
571	*S. frontalis*	16	36.498	4.679	CONT	CAMP	12.8	22.1	52.0	74.0	-0.040	-0.027	Yes	Yes	Yes
572	*S. frontalis*	16	31.792	4.964	CONT	CAMP	15.0	25.0	58.0	77.0	0.007	0.019	Yes	Yes	Yes
573	*S. frontalis*	16	30.074	4.684	CONT	CAMP	12.0	21.5	49.9	69.9	-0.072	-0.039	Yes	Yes	Yes
574	*S. frontalis*	16	26.566	3.602	CONT	CAMP	13.5	23.5	55.5	72.8	-0.028	-0.011	Yes	Yes	Yes
575	*S. frontalis*	16	29.411	4.632	CONT	CAMP	-	-	-	-	-	-	No	Yes	Yes
313	*T. sayaca*	10	25.378	3.426	AIR	CAMP	29.5	24.0	91.0	68.0	-0.006	-0.042	Yes	Yes	Yes
314	*T. sayaca*	10	27.163	4.299	AIR	CAMP	-	-	-	-	-	-	No	Yes	Yes
315	*T. sayaca*	10	27.878	4.726	AIR	CAMP	30.0	24.0	85.0	65.0	-0.013	-0.033	Yes	Yes	Yes
316	*T. sayaca*	10	22.061	4.195	AIR	CAMP	37.0	26.0	92.0	67.0	0.029	0.049	Yes	Yes	Yes
317	*T. sayaca*	10	24.239	3.580	AIR	CAMP	33.5	24.9	89.5	60.5	0.007	0.010	Yes	Yes	Yes
318	*T. sayaca*	10	27.779	4.775	AIR	CAMP	29.5	23.6	83.6	62.6	-0.022	-0.039	Yes	Yes	Yes
319	*T. sayaca*	10	24.355	3.298	AIR	CAMP	31.0	24.0	90.0	70.0	-0.007	-0.020	Yes	Yes	Yes
320	*T. sayaca*	10	16.736	3.469	AIR	CAMP	35.0	24.7	92.7	74.8	0.009	0.029	Yes	Yes	Yes
321	*T. sayaca*	10	20.138	3.373	AIR	CAMP	34.0	25.3	88.2	64.2	0.013	0.016	Yes	Yes	Yes
322	*T. sayaca*	10	25.801	3.860	AIR	CAMP	34.5	24.6	88.0	63.2	0.000	0.025	Yes	Yes	Yes
323	*T. sayaca*	10	19.611	3.797	AIR	CAMP	30.0	23.0	89.4	67.0	-0.026	-0.031	Yes	Yes	Yes

**ID**	**Species**	**Plate**	**CORTf (pg/mg)**	**CORTf (pg/mm)**	**Site type**	**Region**	**Body weight**	**Tarsus**	**Wing**	**Tail**	**Body size**	**Body condition**	**D_1**	**D_2**	**D_3**
72	*T. sayaca*	2	53.353	8.588	CONT	CAMP	-	-	-	-	-	-	No	Yes	Yes
73	*T. sayaca*	2	11.069	1.983	CONT	CAMP	-	-	-	-	-	-	No	Yes	Yes
324	*T. sayaca*	10	16.582	2.729	CONT	CAMP	34.0	26.0	90.0	65.0	0.026	0.013	Yes	Yes	Yes
325	*T. sayaca*	9	21.369	4.011	CONT	CAMP	32.0	24.0	92.0	67.0	-0.005	-0.007	Yes	Yes	Yes
326	*T. sayaca*	17	24.097	4.702	CONT	CAMP	-	-	-	-	-	-	No	Yes	Yes
327	*T. sayaca*	9	53.587	8.356	CONT	CAMP	29.0	24.1	80.7	59.8	-0.017	-0.047	Yes	Yes	Yes
328	*T. sayaca*	9	26.550	4.627	CONT	CAMP	31.0	27.0	92.0	70.0	0.045	-0.031	Yes	Yes	Yes
329	*T. sayaca*	9	85.307	14.069	CONT	CAMP	29.0	28.0	95.0	66.0	0.063	-0.064	Yes	Yes	Yes
330	*T. sayaca*	9	36.625	6.691	CONT	CAMP	37.0	26.0	90.0	66.0	0.026	0.050	Yes	Yes	Yes
331	*T. sayaca*	9	43.415	7.755	CONT	CAMP	30.5	26.0	93.0	68.0	0.030	-0.035	Yes	Yes	Yes
332	*T. sayaca*	9	16.142	2.774	CONT	CAMP	30.0	21.0	90.0	70.0	-0.063	-0.023	Yes	Yes	Yes
333	*T. sayaca*	9	27.443	5.053	CONT	CAMP	32.0	25.0	92.0	62.0	0.012	-0.010	Yes	Yes	Yes
334	*T. sayaca*	9	24.973	5.162	CONT	CAMP	34.0	25.0	95.0	65.0	0.015	0.015	Yes	Yes	Yes
335	*T. sayaca*	9	23.045	4.432	AIR	SAL	-	-	-	-	-	-	No	Yes	Yes
336	*T. sayaca*	9	27.515	4.194	AIR	SAL	-	-	-	-	-	-	No	Yes	Yes
337	*T. sayaca*	9	22.529	4.145	AIR	SAL	-	-	-	-	-	-	No	Yes	Yes
338	*T. sayaca*	9	20.954	3.745	AIR	SAL	-	-	-	-	-	-	No	Yes	Yes
339	*T. sayaca*	9	20.902	4.315	AIR	SAL	-	-	-	-	-	-	No	Yes	Yes
341	*T. sayaca*	9	22.483	3.876	AIR	SAL	-	-	-	-	-	-	No	Yes	Yes
342	*T. sayaca*	9	13.733	3.049	AIR	SAL	32.5	25.0	84.0	72.0	0.003	-0.002	Yes	Yes	Yes
343	*T. sayaca*	9	17.328	3.452	AIR	SAL	31.0	23.0	89.0	65.0	-0.027	-0.016	Yes	Yes	Yes
344	*T. sayaca*	9	35.693	6.005	AIR	SAL	-	-	-	-	-	-	No	Yes	Yes
345	*T. sayaca*	9	13.176	2.667	AIR	SAL	-	-	-	-	-	-	No	Yes	Yes
346	*T. sayaca*	9	28.797	5.352	AIR	SAL	-	-	-	-	-	-	No	Yes	Yes
347	*T. sayaca*	9	15.170	3.046	AIR	SAL	-	-	-	-	-	-	No	Yes	Yes
348	*T. sayaca*	9	30.532	6.082	AIR	SAL	-	-	-	-	-	-	No	Yes	Yes
349	*T. sayaca*	9	14.242	2.912	AIR	SAL	-	-	-	-	-	-	No	Yes	Yes
350	*T. sayaca*	9	27.412	5.159	AIR	SAL	-	-	-	-	-	-	No	Yes	Yes
351	*T. sayaca*	9	14.274	2.543	AIR	SAL	-	-	-	-	-	-	No	Yes	Yes
352	*T. sayaca*	9	15.969	2.692	AIR	SAL	-	-	-	-	-	-	No	Yes	Yes
353	*T. sayaca*	9	14.363	2.599	AIR	SAL	-	-	-	-	-	-	No	Yes	Yes
354	*T. sayaca*	9	38.251	6.235	AIR	SAL	31.5	22.0	95.0	62.0	-0.039	-0.007	Yes	Yes	Yes

ID	Species	Plate	$CORT_f$ (pg/mg)	$CORT_f$ (pg/mm)	Site type	Region	Body weight	Tarsus	Wing	Tail	Body size	Body condition	D_1	D_2	D_3
355	*T. sayaca*	9	13.523	2.358	AIR	SAL	33.5	25.0	90.0	66.0	0.010	0.010	Yes	Yes	Yes
74	*T. sayaca*	2	46.249	9.788	CONT	SAL	-	-	-	-	-	-	No	Yes	Yes
356	*T. sayaca*	17	36.762	6.839	CONT	SAL	33.5	24.0	92.0	70.0	-0.005	0.013	Yes	Yes	Yes
357	*T. sayaca*	9	15.351	2.640	CONT	SAL	32.5	23.0	85.0	70.0	-0.031	0.005	Yes	Yes	Yes
358	*T. sayaca*	9	29.665	5.956	CONT	SAL	37.5	23.0	91.0	70.0	-0.024	0.066	Yes	Yes	Yes
359	*T. sayaca*	9	17.421	2.890	CONT	SAL	40.5	24.0	86.0	69.0	-0.012	0.097	Yes	Yes	Yes
360	*T. sayaca*	9	22.724	4.090	CONT	SAL	33.0	25.0	88.0	64.0	0.007	0.004	Yes	Yes	Yes
361	*T. sayaca*	9	10.822	2.164	CONT	SAL	33.5	25.0	87.0	70.0	0.007	0.010	Yes	Yes	Yes
362	*T. sayaca*	9	21.144	3.827	CONT	SAL	32.5	24.0	89.0	65.0	-0.009	0.000	Yes	Yes	Yes
363	*T. sayaca*	9	30.782	5.130	CONT	SAL	32.5	25.0	85.0	70.0	0.004	-0.002	Yes	Yes	Yes
506	*T. musculus*	13	438.535	42.392	AIR	BRAS	12.5	19.0	50.0	45.0	-0.007	0.031	Yes	Yes	Yes
507	*T. musculus*	13	149.847	14.799	AIR	BRAS	13.0	24.0	53.0	49.0	0.042	0.022	Yes	Yes	Yes
508	*T. musculus*	13	45.493	4.354	AIR	BRAS	13.5	22.0	52.0	47.0	0.021	0.050	Yes	Yes	Yes
509	*T. musculus*	13	161.055	16.357	AIR	BRAS	12.0	20.0	54.0	49.0	0.043	-0.014	Yes	Yes	Yes
511	*T. musculus*	13	1205.900	140.198	AIR	BRAS	12.0	20.0	53.0	46.0	0.015	0.001	Yes	Yes	Yes
512	*T. musculus*	13	164.449	16.234	AIR	BRAS	13.0	20.0	52.0	45.0	0.003	0.043	Yes	Yes	Yes
513	*T. musculus*	13	41.937	3.851	AIR	BRAS	11.5	24.0	53.0	47.0	0.026	-0.023	Yes	Yes	Yes
514	*T. musculus*	13	258.999	25.574	AIR	BRAS	-	-	-	-	-	-	No	Yes	Yes
515	*T. musculus*	13	101.286	9.414	AIR	BRAS	12.5	21.0	55.0	50.0	0.055	-0.002	Yes	Yes	Yes
516	*T. musculus*	13	97.747	10.658	AIR	BRAS	-	-	-	-	-	-	No	Yes	Yes
510	*T. musculus*	13	515.004	49.105	CONT	BRAS	12.0	22.0	56.0	50.0	0.060	-0.023	Yes	Yes	Yes
517	*T. musculus*	13	97.187	10.158	CONT	BRAS	13.0	20.8	53.2	46.5	0.021	0.033	Yes	Yes	Yes
518	*T. musculus*	13	331.782	26.213	CONT	BRAS	12.0	23.0	53.0	42.0	-0.016	0.018	Yes	Yes	Yes
519	*T. musculus*	13	661.358	52.909	CONT	BRAS	11.5	20.0	56.0	52.0	0.073	-0.048	Yes	Yes	Yes
520	*T. musculus*	13	334.285	34.326	CONT	BRAS	13.0	22.0	55.0	45.0	0.017	0.035	Yes	Yes	Yes
521	*T. musculus*	13	1758.314	182.865	CONT	BRAS	12.0	21.0	55.0	47.0	0.032	-0.008	Yes	Yes	Yes
522	*T. musculus*	13	623.352	59.842	CONT	BRAS	13.0	22.0	53.0	44.0	0.000	0.044	Yes	Yes	Yes
523	*T. musculus*	13	348.234	31.698	CONT	BRAS	13.0	21.0	54.0	46.0	0.020	0.033	Yes	Yes	Yes
524	*T. musculus*	13	803.086	75.615	CONT	BRAS	12.0	20.0	51.0	45.0	-0.001	0.010	Yes	Yes	Yes
525	*T. musculus*	13	212.469	19.989	CONT	BRAS	-	-	-	-	-	-	No	Yes	Yes
88	*T. musculus*	3	419.890	34.895	AIR	CAMP	-	-	-	-	-	-	No	Yes	Yes
526	*T. musculus*	13	16.187	1.461	AIR	CAMP	13.0	20.4	51.6	43.6	-0.010	0.050	Yes	Yes	Yes

ID	Species	Plate	CORT$_f$ (pg/mg)	CORT$_f$ (pg/mm)	Site type	Region	Body weight	Tarsus	Wing	Tail	Body size	Body condition	D$_1$	D$_2$	D$_3$
527	*T. musculus*	13	281.626	23.554	AIR	CAMP	11.0	22.0	49.0	41.0	-0.044	-0.005	Yes	Yes	Yes
528	*T. musculus*	13	38.110	3.449	AIR	CAMP	-	-	-	-	-	-	No	Yes	Yes
529	*T. musculus*	13	32.221	2.987	AIR	CAMP	12.0	22.0	55.0	43.0	0.000	0.009	Yes	Yes	Yes
531	*T. musculus*	13	31.372	2.890	AIR	CAMP	-	-	-	-	-	-	No	Yes	Yes
532	*T. musculus*	13	62.624	6.355	AIR	CAMP	13.0	23.0	55.0	50.0	0.057	0.014	Yes	Yes	Yes
533	*T. musculus*	13	112.948	10.996	AIR	CAMP	11.5	20.2	50.7	42.3	-0.026	0.005	Yes	Yes	Yes
534	*T. musculus*	13	53.332	4.933	AIR	CAMP	-	-	-	-	-	-	No	Yes	Yes
535	*T. musculus*	13	64.113	6.554	AIR	CAMP	13.0	21.0	57.0	47.0	0.040	0.023	Yes	Yes	Yes
536	*T. musculus*	13	157.031	14.773	AIR	CAMP	12.0	21.0	52.0	47.0	0.020	-0.001	Yes	Yes	Yes
537	*T. musculus*	13	328.838	33.334	AIR	CAMP	11.0	21.0	49.3	44.3	-0.014	-0.021	Yes	Yes	Yes
538	*T. musculus*	13	24.255	2.249	AIR	CAMP	-	-	-	-	-	-	No	Yes	Yes
87	*T. musculus*	3	26.369	2.119	CONT	CAMP	-	-	-	-	-	-	No	Yes	Yes
539	*T. musculus*	13	57.828	4.909	CONT	CAMP	10.5	21.0	54.0	44.0	0.004	-0.051	Yes	Yes	Yes
540	*T. musculus*	13	93.642	8.018	CONT	CAMP	11.0	21.0	48.0	44.0	-0.023	-0.016	Yes	Yes	Yes
541	*T. musculus*	13	111.215	9.954	CONT	CAMP	-	-	-	-	-	-	No	Yes	Yes
542	*T. musculus*	13	216.539	19.098	CONT	CAMP	12.5	22.1	53.7	44.8	0.010	0.022	Yes	Yes	Yes
543	*T. musculus*	13	117.997	10.923	CONT	CAMP	12.0	21.0	51.0	42.0	-0.026	0.023	Yes	Yes	Yes
544	*T. musculus*	13	88.284	8.083	CONT	CAMP	10.0	21.0	49.0	41.0	-0.044	-0.046	Yes	Yes	Yes
545	*T. musculus*	13	102.219	8.850	CONT	CAMP	-	-	-	-	-	-	No	Yes	Yes
546	*T. musculus*	13	16.023	1.345	CONT	CAMP	13.0	21.4	46.4	41.6	-0.051	0.071	Yes	Yes	Yes
547	*T. musculus*	13	124.864	10.565	CONT	CAMP	-	-	-	-	-	-	No	Yes	Yes
548	*T. musculus*	14	425.468	41.792	CONT	CAMP	-	-	-	-	-	-	No	Yes	Yes
549	*T. musculus*	14	90.476	8.410	CONT	CAMP	12.0	22.0	51.0	44.0	-0.008	0.014	Yes	Yes	Yes
530	*T. musculus*	13	58.196	5.613	AIR	SAL	-	-	-	-	-	-	No	Yes	Yes
560	*T. musculus*	14	42.523	3.951	AIR	SAL	-	-	-	-	-	-	No	Yes	Yes
561	*T. musculus*	14	48.414	4.085	AIR	SAL	11.5	22.0	57.0	47.0	0.041	-0.031	Yes	Yes	Yes
562	*T. musculus*	14	34.416	3.263	AIR	SAL	11.0	21.0	50.0	43.0	-0.022	-0.017	Yes	Yes	Yes
563	*T. musculus*	14	32.176	2.900	AIR	SAL	11.0	20.0	51.0	45.0	-0.001	-0.028	Yes	Yes	Yes
564	*T. musculus*	14	46.081	3.873	AIR	SAL	10.5	23.0	50.0	39.0	-0.057	-0.018	Yes	Yes	Yes
565	*T. musculus*	14	21.211	2.106	AIR	SAL	12.0	21.0	49.0	41.0	-0.044	0.033	Yes	Yes	Yes
566	*T. musculus*	14	27.757	2.678	AIR	SAL	10.0	22.0	53.0	40.0	-0.035	-0.051	Yes	Yes	Yes
567	*T. musculus*	14	42.655	4.015	AIR	SAL	-	-	-	-	-	-	No	Yes	Yes

ID	Species	Plate	CORTf (pg/mg)	CORTf (pg/mm)	Site type	Region	Body weight	Tarsus	Wing	Tail	Body size	Body condition	D_1	D_2	D_3
568	*T. musculus*	14	56.968	5.329	AIR	SAL	-	-	-	-	-	-	No	Yes	Yes
569	*T. musculus*	14	30.524	2.811	AIR	SAL	-	-	-	-	-	-	No	Yes	Yes
570	*T. musculus*	14	58.801	4.625	AIR	SAL	10.0	20.0	53.0	43.0	-0.010	-0.065	Yes	Yes	Yes
550	*T. musculus*	14	107.418	10.303	CONT	SAL	9.5	21.0	50.0	40.0	-0.049	-0.066	Yes	Yes	Yes
551	*T. musculus*	14	44.009	4.341	CONT	SAL	12.0	21.0	51.0	43.0	-0.018	0.019	Yes	Yes	Yes
552	*T. musculus*	14	85.833	8.338	CONT	SAL	11.5	21.0	50.0	42.0	-0.031	0.007	Yes	Yes	Yes
553	*T. musculus*	14	203.189	18.300	CONT	SAL	11.5	21.0	52.0	45.0	0.004	-0.011	Yes	Yes	Yes
554	*T. musculus*	14	82.742	7.612	CONT	SAL	12.5	22.0	52.0	41.0	-0.030	0.043	Yes	Yes	Yes
555	*T. musculus*	14	83.163	7.136	CONT	SAL	10.5	20.0	50.0	43.0	-0.023	-0.036	Yes	Yes	Yes
556	*T. musculus*	14	30.583	2.838	CONT	SAL	11.0	22.0	50.0	43.0	-0.021	-0.017	Yes	Yes	Yes
557	*T. musculus*	14	34.349	3.209	CONT	SAL	-	-	-	-	-	-	No	Yes	Yes
558	*T. musculus*	14	23.090	2.162	CONT	SAL	-	-	-	-	-	-	No	Yes	Yes
559	*T. musculus*	14	42.237	3.760	CONT	SAL	10.5	21.0	53.0	45.0	0.008	-0.053	Yes	Yes	Yes
64	*T. leucomelas*	5	43.465	9.173	AIR	BRAS	-	-	-	-	-	-	No	Yes	No
65	*T. leucomelas*	5	51.981	11.768	AIR	BRAS	-	-	-	-	-	-	No	Yes	No
66	*T. leucomelas*	5	88.584	21.287	AIR	BRAS	58.0	32.0	122.0	83.0	-0.079	0.007	Yes	Yes	No
67	*T. leucomelas*	5	45.245	9.555	AIR	BRAS	57.0	42.0	120.0	103.0	0.068	-0.079	Yes	Yes	No
68	*T. leucomelas*	5	24.598	5.434	AIR	BRAS	58.0	36.0	116.0	95.0	-0.009	-0.031	Yes	Yes	No
69	*T. leucomelas*	5	24.491	5.233	AIR	BRAS	62.0	34.0	118.0	102.0	-0.013	0.001	Yes	Yes	No
94	*T. leucomelas*	5	28.829	4.964	AIR	BRAS	56.0	31.1	109.8	91.8	-0.073	-0.011	Yes	Yes	No
95	*T. leucomelas*	5	21.286	3.291	AIR	BRAS	69.0	32.4	123.0	95.0	-0.044	0.064	Yes	Yes	No
96	*T. leucomelas*	5	44.734	7.978	AIR	BRAS	51.0	32.0	114.0	93.0	-0.058	-0.060	Yes	Yes	No
97	*T. leucomelas*	5	20.892	3.511	AIR	BRAS	-	-	-	-	-	-	No	Yes	No
98	*T. leucomelas*	5	26.335	4.709	AIR	BRAS	50.0	34.0	120.0	94.0	-0.030	-0.084	Yes	Yes	No
99	*T. leucomelas*	5	20.517	3.612	AIR	BRAS	-	-	-	-	-	-	No	Yes	No
100	*T. leucomelas*	5	33.743	5.887	AIR	BRAS	-	-	-	-	-	-	No	Yes	No
101	*T. leucomelas*	5	18.143	3.320	AIR	BRAS	-	-	-	-	-	-	No	Yes	No
102	*T. leucomelas*	5	21.787	3.821	CONT	BRAS	50.0	33.0	113.5	85.0	-0.067	-0.064	Yes	Yes	No
138	*T. leucomelas*	7	26.234	4.014	AIR	CAMP	68.0	38.0	125.0	104.0	0.036	0.015	Yes	Yes	Yes
139	*T. leucomelas*	7	38.220	6.762	AIR	CAMP	68.0	36.0	115.0	94.0	-0.011	0.040	Yes	Yes	Yes
140	*T. leucomelas*	7	17.777	3.058	AIR	CAMP	63.0	37.0	120.0	91.0	-0.006	0.004	Yes	Yes	Yes
141	*T. leucomelas*	7	20.693	3.467	AIR	CAMP	59.0	37.4	113.0	98.7	0.012	-0.035	Yes	Yes	Yes

ID	Species	Plate	CORTf (pg/mg)	CORTf (pg/mm)	Site type	Region	Body weight	Tarsus	Wing	Tail	Body size	Body condition	D_1	D_2	D_3
142	*T. leucomelas*	7	36.782	6.373	AIR	CAMP	67.0	34.0	110.0	95.0	-0.033	0.045	Yes	Yes	Yes
143	*T. leucomelas*	7	36.715	5.934	AIR	CAMP	69.0	37.5	118.0	101.0	0.021	0.029	Yes	Yes	Yes
144	*T. leucomelas*	7	16.229	2.595	AIR	CAMP	73.0	38.7	113.7	104.5	0.038	0.044	Yes	Yes	Yes
145	*T. leucomelas*	7	13.460	2.390	AIR	CAMP	64.0	37.2	116.5	99.1	0.013	0.000	Yes	Yes	Yes
146	*T. leucomelas*	7	9.179	1.684	CONT	CAMP	-	-	-	-	-	-	No	Yes	Yes
147	*T. leucomelas*	7	10.909	1.896	CONT	CAMP	67.0	40.0	120.0	102.0	0.048	0.002	Yes	Yes	Yes
148	*T. leucomelas*	7	18.168	3.038	CONT	CAMP	68.0	39.0	119.0	102.0	0.038	0.013	Yes	Yes	Yes
149	*T. leucomelas*	7	60.715	10.974	CONT	CAMP	65.0	36.9	111.2	100.6	0.011	0.008	Yes	Yes	Yes
150	*T. leucomelas*	7	19.597	6.973	CONT	CAMP	66.0	40.0	120.0	110.0	0.083	0.032	Yes	Yes	Yes
151	*T. leucomelas*	7	69.782	12.073	CONT	CAMP	-	-	-	-	-	-	No	Yes	Yes
152	*T. leucomelas*	7	41.980	7.231	CONT	CAMP	-	-	-	-	-	-	No	Yes	Yes
153	*T. leucomelas*	7	13.038	2.251	CONT	CAMP	75.0	43.0	125.0	105.0	-0.043	0.031	Yes	Yes	Yes
154	*T. leucomelas*	7	18.819	3.133	CONT	CAMP	64.0	32.0	120.0	98.0	-0.051	0.067	Yes	Yes	Yes
155	*T. leucomelas*	7	24.877	4.124	CONT	CAMP	69.0	32.0	114.0	96.0	0.024	-0.041	Yes	Yes	Yes
156	*T. leucomelas*	7	16.877	2.825	CONT	CAMP	59.0	39.0	113.0	97.0	0.030	0.005	Yes	Yes	Yes
157	*T. leucomelas*	7	16.948	2.819	CONT	CAMP	66.0	37.7	124.2	103.0	0.016	0.047	Yes	Yes	Yes
158	*T. leucomelas*	7	13.605	2.322	CONT	CAMP	71.5	37.1	115.0	101.0	0.065	0.036	Yes	Yes	Yes
159	*T. leucomelas*	7	16.777	2.713	CONT	CAMP	74.0	42.0	117.0	102.0	-0.043	-0.005	Yes	Yes	Yes
103	*T. leucomelas*	5	12.718	2.107	AIR	SAL	69.0	38.0	115.0	95.0	0.011	0.034	Yes	Yes	Yes
104	*T. leucomelas*	5	20.214	3.591	AIR	SAL	-	-	-	-	-	-	No	Yes	Yes
105	*T. leucomelas*	5	13.644	2.442	AIR	SAL	60.5	39.0	113.0	101.0	0.033	-0.035	Yes	Yes	Yes
106	*T. leucomelas*	5	13.503	2.459	AIR	SAL	-	-	-	-	-	-	No	Yes	Yes
107	*T. leucomelas*	5	18.363	3.246	AIR	SAL	-	-	-	-	-	-	No	Yes	Yes
108	*T. leucomelas*	5	27.759	5.131	AIR	SAL	-	-	-	-	-	-	No	Yes	Yes
109	*T. leucomelas*	5	19.575	3.340	AIR	SAL	-	-	-	-	-	-	No	Yes	Yes
110	*T. leucomelas*	6	19.891	3.576	AIR	SAL	-	-	-	-	-	-	No	Yes	Yes
111	*T. leucomelas*	6	21.648	4.017	AIR	SAL	-	-	-	-	-	-	No	Yes	Yes
112	*T. leucomelas*	6	33.190	6.328	AIR	SAL	-	-	-	-	-	-	No	Yes	Yes
113	*T. leucomelas*	6	21.091	3.716	AIR	SAL	-	-	-	-	-	-	No	Yes	Yes
114	*T. leucomelas*	6	30.240	5.371	AIR	SAL	-	-	-	-	-	-	No	Yes	Yes
115	*T. leucomelas*	6	23.836	4.242	AIR	SAL	63.0	38.0	115.0	96.0	0.013	-0.006	Yes	Yes	Yes
116	*T. leucomelas*	6	13.293	2.542	AIR	SAL	59.5	37.0	116.0	100.0	0.013	-0.031	Yes	Yes	Yes

ID	Species	Plate	CORTf (pg/mg)	CORTf (pg/mm)	Site type	Region	Body weight	Tarsus	Wing	Tail	Body size	Body condition	D_1	D_2	D_3
117	*T. leucomelas*	6	19.886	3.462	AIR	SAL	64.0	38.0	120.0	105.0	0.035	-0.012	Yes	Yes	Yes
118	*T. leucomelas*	6	19.959	3.552	AIR	SAL	62.5	36.0	121.0	101.0	0.007	-0.007	Yes	Yes	Yes
119	*T. leucomelas*	7	22.011	4.081	AIR	SAL	72.5	36.0	110.0	100.0	0.000	0.062	Yes	Yes	Yes
120	*T. leucomelas*	7	25.556	4.608	AIR	SAL	63.0	36.0	115.0	94.0	-0.011	0.007	Yes	Yes	Yes
121	*T. leucomelas*	7	11.496	2.207	AIR	SAL	-	-	-	-	-	-	No	Yes	Yes
122	*T. leucomelas*	7	17.587	3.198	CONT	SAL	62.5	38.0	115.0	99.0	0.020	-0.014	Yes	Yes	Yes
123	*T. leucomelas*	7	32.169	5.939	CONT	SAL	59.5	36.0	110.0	91.0	-0.021	-0.013	Yes	Yes	Yes
124	*T. leucomelas*	7	11.841	2.251	CONT	SAL	64.0	33.0	112.0	85.0	-0.067	0.043	Yes	Yes	Yes
125	*T. leucomelas*	7	19.760	3.662	CONT	SAL	59.0	36.0	112.0	98.0	-0.004	-0.026	Yes	Yes	Yes
126	*T. leucomelas*	7	15.141	2.724	CONT	SAL	59.5	34.0	114.0	99.0	-0.021	-0.013	Yes	Yes	Yes
127	*T. leucomelas*	7	21.307	3.854	CONT	SAL	65.5	40.0	113.0	99.0	0.038	-0.003	Yes	Yes	Yes
128	*T. leucomelas*	7	27.915	5.123	CONT	SAL	64.0	38.0	115.0	90.0	-0.001	0.008	Yes	Yes	Yes
129	*T. leucomelas*	7	32.590	6.518	CONT	SAL	55.0	36.0	118.0	95.0	-0.008	-0.054	Yes	Yes	Yes
130	*T. leucomelas*	10	12.531	2.278	CONT	SAL	67.0	35.0	110.0	94.0	-0.024	0.040	Yes	Yes	Yes
131	*T. leucomelas*	10	31.461	5.178	CONT	SAL	62.5	35.0	113.0	101.0	-0.007	0.001	Yes	Yes	Yes
132	*T. leucomelas*	10	12.164	2.087	CONT	SAL	60.0	34.0	116.0	88.0	-0.047	0.004	Yes	Yes	Yes
133	*T. leucomelas*	10	8.868	1.536	CONT	SAL	62.5	36.0	116.0	100.0	0.003	-0.004	Yes	Yes	Yes
134	*T. leucomelas*	10	22.480	3.465	CONT	SAL	63.0	39.0	115.0	104.0	0.041	-0.021	Yes	Yes	Yes
135	*T. leucomelas*	10	6.904	1.220	CONT	SAL	64.0	39.0	115.0	99.0	0.030	-0.008	Yes	Yes	Yes
136	*T. leucomelas*	7	47.237	8.310	CONT	SAL	72.5	39.0	119.0	101.0	0.036	0.042	Yes	Yes	Yes
137	*T. leucomelas*	7	28.219	4.974	CONT	SAL	52.5	35.0	109.0	100.0	-0.011	-0.073	Yes	Yes	Yes
597	*T. rufiventris*	16	17.911	5.914	AIR	BRAS	-	-	-	-	-	-	No	Yes	No
598	*T. rufiventris*	16	16.569	5.121	AIR	BRAS	-	-	-	-	-	-	No	Yes	No
599	*T. rufiventris*	16	17.821	5.311	AIR	BRAS	-	-	-	-	-	-	No	Yes	No
602	*T. rufiventris*	16	20.523	6.199	CONT	BRAS	-	-	-	-	-	-	No	Yes	No
603	*T. rufiventris*	16	14.399	4.555	CONT	BRAS	-	-	-	-	-	-	No	Yes	No
604	*T. rufiventris*	16	12.105	3.620	CONT	CAMP	-	-	-	-	-	-	No	Yes	No
605	*T. rufiventris*	16	16.095	5.160	CONT	CAMP	-	-	-	-	-	-	No	Yes	No
600	*T. rufiventris*	16	28.504	8.745	AIR	SAL	-	-	-	-	-	-	No	Yes	No
601	*T. rufiventris*	16	16.700	4.896	AIR	SAL	-	-	-	-	-	-	No	Yes	No
606	*T. rufiventris*	16	18.287	5.642	CONT	SAL	-	-	-	-	-	-	No	Yes	No
607	*T. rufiventris*	16	15.751	4.382	CONT	SAL	-	-	-	-	-	-	No	Yes	No

ID	Species	Plate	$CORT_f$ (pg/mg)	$CORT_f$ (pg/mm)	Site type	Region	Body weight	Tarsus	Wing	Tail	Body size	Body condition	D_1	D_2	D_3
608	*T. rufiventris*	16	13.250	3.950	CONT	SAL	-	-	-	-	-	-	No	Yes	No
609	*T. rufiventris*	16	12.948	3.951	CONT	SAL	-	-	-	-	-	-	No	Yes	No
1	*V. jacarina*	1	81.318	7.547	AIR	BRAS	-	-	-	-	-	-	No	Yes	Yes
2	*V. jacarina*	1	31.401	3.493	AIR	BRAS	-	-	-	-	-	-	No	Yes	Yes
3	*V. jacarina*	1	36.807	3.096	AIR	BRAS	-	-	-	-	-	-	No	Yes	Yes
4	*V. jacarina*	1	42.648	4.330	AIR	BRAS	-	-	-	-	-	-	No	Yes	Yes
5	*V. jacarina*	1	16.271	1.988	AIR	BRAS	-	-	-	-	-	-	No	Yes	Yes
9	*V. jacarina*	5	24.984	3.980	AIR	BRAS	-	-	-	-	-	-	No	Yes	Yes
10	*V. jacarina*	4	16.661	1.803	AIR	BRAS	-	-	-	-	-	-	No	Yes	Yes
11	*V. jacarina*	4	16.814	1.737	AIR	BRAS	-	-	-	-	-	-	No	Yes	Yes
12	*V. jacarina*	1	19.405	1.916	AIR	BRAS	-	-	-	-	-	-	No	Yes	Yes
13	*V. jacarina*	4	19.372	2.133	AIR	BRAS	-	-	-	-	-	-	No	Yes	Yes
14	*V. jacarina*	4	17.415	1.818	AIR	BRAS	-	-	-	-	-	-	No	Yes	Yes
15	*V. jacarina*	1	45.186	4.217	AIR	BRAS	-	-	-	-	-	-	No	Yes	Yes
18	*V. jacarina*	4	27.165	2.564	AIR	BRAS	-	-	-	-	-	-	No	Yes	Yes
19	*V. jacarina*	1	36.011	3.684	AIR	BRAS	-	-	-	-	-	-	No	Yes	Yes
20	*V. jacarina*	4	40.558	4.084	AIR	BRAS	-	-	-	-	-	-	No	Yes	Yes
21	*V. jacarina*	4	47.951	4.299	AIR	BRAS	-	-	-	-	-	-	No	Yes	Yes
22	*V. jacarina*	1	27.047	2.968	AIR	BRAS	-	-	-	-	-	-	No	Yes	Yes
23	*V. jacarina*	7	76.540	7.026	AIR	BRAS	-	-	-	-	-	-	No	Yes	Yes
24	*V. jacarina*	1	17.573	1.779	AIR	BRAS	-	-	-	-	-	-	No	Yes	Yes
25	*V. jacarina*	4	18.986	1.770	AIR	BRAS	-	-	-	-	-	-	No	Yes	Yes
26	*V. jacarina*	4	13.288	1.293	AIR	BRAS	-	-	-	-	-	-	No	Yes	Yes
27	*V. jacarina*	4	27.295	2.630	AIR	BRAS	-	-	-	-	-	-	No	Yes	Yes
28	*V. jacarina*	1	42.414	4.459	AIR	BRAS	-	-	-	-	-	-	No	Yes	Yes
29	*V. jacarina*	1	26.907	2.653	AIR	BRAS	-	-	-	-	-	-	No	Yes	Yes
30	*V. jacarina*	1	69.862	7.340	AIR	BRAS	-	-	-	-	-	-	No	Yes	Yes
31	*V. jacarina*	1	20.383	2.192	AIR	BRAS	-	-	-	-	-	-	No	Yes	Yes
32	*V. jacarina*	1	10.788	1.070	AIR	BRAS	-	-	-	-	-	-	No	Yes	Yes
33	*V. jacarina*	4	28.249	3.189	AIR	BRAS	-	-	-	-	-	-	No	Yes	Yes
34	*V. jacarina*	4	16.067	1.617	AIR	BRAS	-	-	-	-	-	-	No	Yes	Yes
35	*V. jacarina*	4	18.898	2.042	AIR	BRAS	-	-	-	-	-	-	No	Yes	Yes

ID	Species	Plate	$CORT_f$ (pg/mg)	$CORT_f$ (pg/mm)	Site type	Region	Body weight	Tarsus	Wing	Tail	Body size	Body condition	D_1	D_2	D_3
7	*V. jacarina*	1	32.320	3.138	CONT	BRAS	-	-	-	-	-	-	No	Yes	Yes
8	*V. jacarina*	1	32.298	3.310	CONT	BRAS	-	-	-	-	-	-	No	Yes	Yes
16	*V. jacarina*	4	30.675	3.049	CONT	BRAS	-	-	-	-	-	-	No	Yes	Yes
17	*V. jacarina*	1	36.157	3.942	CONT	BRAS	-	-	-	-	-	-	No	Yes	Yes
36	*V. jacarina*	1	10.924	1.049	CONT	BRAS	-	-	-	-	-	-	No	Yes	Yes
37	*V. jacarina*	1	47.490	4.981	CONT	BRAS	-	-	-	-	-	-	No	Yes	Yes
38	*V. jacarina*	1	13.790	1.501	CONT	BRAS	-	-	-	-	-	-	No	Yes	Yes
39	*V. jacarina*	1	20.991	2.092	CONT	BRAS	-	-	-	-	-	-	No	Yes	Yes
40	*V. jacarina*	1	16.344	1.519	CONT	BRAS	-	-	-	-	-	-	No	Yes	Yes
41	*V. jacarina*	2	13.805	1.304	CONT	BRAS	-	-	-	-	-	-	No	Yes	Yes
42	*V. jacarina*	2	34.619	3.338	CONT	BRAS	-	-	-	-	-	-	No	Yes	Yes
43	*V. jacarina*	2	12.769	1.277	CONT	BRAS	-	-	-	-	-	-	No	Yes	Yes
46	*V. jacarina*	2	19.772	2.118	AIR	CAMP	-	-	-	-	-	-	No	Yes	No
47	*V. jacarina*	2	32.188	3.616	AIR	CAMP	-	-	-	-	-	-	No	Yes	No
48	*V. jacarina*	2	43.314	4.123	AIR	CAMP	-	-	-	-	-	-	No	Yes	No
49	*V. jacarina*	2	33.899	3.195	AIR	CAMP	-	-	-	-	-	-	No	Yes	No
50	*V. jacarina*	2	31.602	3.066	AIR	CAMP	-	-	-	-	-	-	No	Yes	No
51	*V. jacarina*	2	31.378	2.799	AIR	CAMP	-	-	-	-	-	-	No	Yes	No
52	*V. jacarina*	2	23.390	2.339	AIR	CAMP	-	-	-	-	-	-	No	Yes	No
53	*V. jacarina*	2	13.761	1.381	AIR	CAMP	-	-	-	-	-	-	No	Yes	No
54	*V. jacarina*	2	28.571	2.490	AIR	CAMP	-	-	-	-	-	-	No	Yes	No
55	*V. jacarina*	2	24.296	2.606	AIR	CAMP	-	-	-	-	-	-	No	Yes	No
56	*V. jacarina*	2	35.830	3.349	AIR	CAMP	-	-	-	-	-	-	No	Yes	No
57	*V. jacarina*	2	21.909	2.137	AIR	CAMP	-	-	-	-	-	-	No	Yes	No
58	*V. jacarina*	2	22.117	2.113	AIR	CAMP	-	-	-	-	-	-	No	Yes	No
59	*V. jacarina*	2	27.876	2.769	AIR	CAMP	-	-	-	-	-	-	No	Yes	No
60	*V. jacarina*	2	21.167	2.170	AIR	CAMP	-	-	-	-	-	-	No	Yes	No
61	*V. jacarina*	2	25.078	2.423	AIR	CAMP	-	-	-	-	-	-	No	Yes	No
62	*V. jacarina*	2	23.951	2.342	AIR	CAMP	-	-	-	-	-	-	No	Yes	No
63	*V. jacarina*	2	32.628	2.888	AIR	CAMP	-	-	-	-	-	-	No	Yes	No
44	*V. jacarina*	2	29.419	3.129	CONT	CAMP	-	-	-	-	-	-	No	Yes	No
45	*V. jacarina*	2	17.254	1.715	CONT	CAMP	-	-	-	-	-	-	No	Yes	No

ID	Species	Plate	CORTf (pg/mg)	CORTf (pg/mm)	Site type	Region	Body weight	Tarsus	Wing	Tail	Body size	Body condition	D_1	D_2	D_3
410	*Z. capensis*	12	31.608	4.089	AIR	BRAS	18.5	25.0	72.0	69.0	0.051	-0.041	Yes	Yes	No
75	*Z. capensis*	12	52.702	7.005	CONT	BRAS	-	-	-	-	-	-	No	Yes	No
76	*Z. capensis*	12	48.146	6.728	CONT	BRAS	-	-	-	-	-	-	No	Yes	No
411	*Z. capensis*	12	21.149	2.849	AIR	CAMP	22.0	25.0	70.0	64.0	0.020	0.032	Yes	Yes	Yes
412	*Z. capensis*	12	33.546	4.500	AIR	CAMP	21.0	26.0	71.0	68.0	0.049	0.014	Yes	Yes	Yes
413	*Z. capensis*	12	41.273	5.456	AIR	CAMP	18.0	26.0	66.0	61.0	-0.003	-0.056	Yes	Yes	Yes
414	*Z. capensis*	12	41.636	5.390	AIR	CAMP	19.0	25.0	65.0	63.0	-0.003	-0.033	Yes	Yes	Yes
415	*Z. capensis*	12	55.248	6.929	AIR	CAMP	-	-	-	-	-	-	No	Yes	Yes
416	*Z. capensis*	12	84.420	9.172	AIR	CAMP	19.0	22.8	59.9	57.0	-0.071	-0.036	Yes	Yes	Yes
417	*Z. capensis*	12	210.988	30.850	AIR	CAMP	19.0	24.4	64.4	58.6	-0.032	-0.034	Yes	Yes	Yes
395	*Z. capensis*	12	33.661	5.261	CONT	CAMP	22.0	26.3	66.9	61.7	0.006	0.032	Yes	Yes	Yes
396	*Z. capensis*	12	29.495	4.282	CONT	CAMP	21.5	26.0	70.0	62.0	0.017	0.022	Yes	Yes	Yes
397	*Z. capensis*	12	102.835	12.418	CONT	CAMP	23.5	24.0	62.0	55.6	-0.061	0.057	Yes	Yes	Yes
398	*Z. capensis*	12	37.875	5.188	CONT	CAMP	20.0	25.0	68.4	62.5	0.007	-0.010	Yes	Yes	Yes
399	*Z. capensis*	12	36.054	5.146	CONT	CAMP	20.0	26.0	68.1	63.4	0.017	-0.009	Yes	Yes	Yes
400	*Z. capensis*	12	90.834	11.137	CONT	CAMP	19.0	23.7	63.7	57.2	-0.047	-0.035	Yes	Yes	Yes
401	*Z. capensis*	12	39.839	5.652	CONT	CAMP	21.0	27.0	70.0	65.0	0.038	0.013	Yes	Yes	Yes
402	*Z. capensis*	12	169.357	23.966	CONT	CAMP	20.0	26.0	70.0	70.0	0.055	-0.007	Yes	Yes	Yes
403	*Z. capensis*	12	25.783	3.424	CONT	CAMP	21.0	25.0	65.0	60.0	-0.018	0.010	Yes	Yes	Yes
404	*Z. capensis*	12	45.951	6.402	CONT	CAMP	21.0	26.0	68.0	64.0	0.020	0.012	Yes	Yes	Yes
405	*Z. capensis*	12	34.994	4.495	CONT	CAMP	20.0	27.0	69.0	61.0	0.015	-0.009	Yes	Yes	Yes
406	*Z. capensis*	12	60.184	6.848	CONT	CAMP	20.0	25.0	64.0	62.0	-0.012	-0.011	Yes	Yes	Yes
407	*Z. capensis*	12	33.361	4.177	CONT	CAMP	23.0	23.0	68.0	64.0	-0.001	0.050	Yes	Yes	Yes
408	*Z. capensis*	12	23.530	2.844	CONT	CAMP	22.5	24.8	62.2	57.4	-0.044	0.039	Yes	Yes	Yes
409	*Z. capensis*	12	50.664	6.795	CONT	CAMP	20.5	24.1	66.8	62.8	-0.004	0.000	Yes	Yes	Yes

C. R scripts and outputs for species-specific analysis, including Global Model and each species-specific model.

Global Model 1

```
data<- read.table("Dados-CORT2023.txt",h=T)
CORT<- data[data$Dataset_CORT=="Sim",]
library(lme4)
library(car)
library(AID)
shapiro.test(CORT$CORTf_leng)
##  Shapiro-Wilk normality test
## data:  CORT$CORTf_leng
## W = 0.36966, p-value < 2.2e-16
```

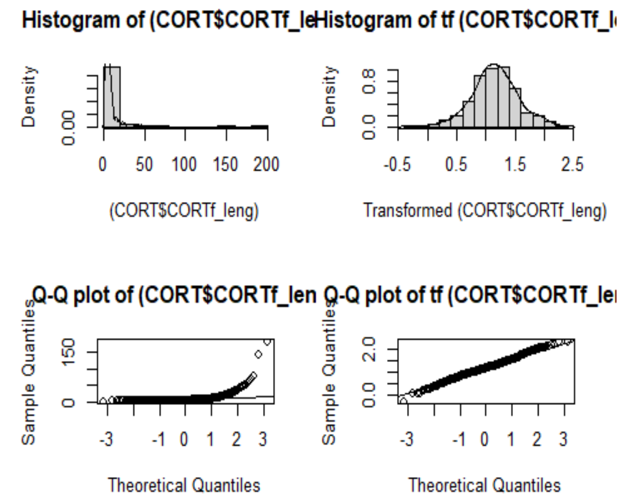


```
boxcoxnc((CORT$CORTf_leng), method='sw')##
##    Box-Cox power transformation
##    lambda.hat : -0.37
##    Shapiro-Wilk normality test for transformed data (alpha
= 0.05)
###    statistic   : 0.9958483
##    p.value     : 0.1171018
##    Result      : Transformed data are normal.

CORT$CORTf_leng_bc <- (((CORT$CORTf_leng)^-0.37)-1)/-0.37
CORT$CORTf_leng_bcS<- scale(CORT$CORTf_leng_bc,center=TRUE)
shapiro.test(CORT$CORTf_leng_bcS)
##  Shapiro-Wilk normality test
## data:  CORT$CORTf_leng_bcS
## W = 0.99585, p-value = 0.1171

my_model<-lmer(CORTf_leng_bcS~Site_type*Species+Region+(1|pl
ate),data=CORT)
summary(my_model)

## Linear mixed model fit by REML ['lmerMod']
## Formula: CORTf_leng_bcS ~ Site_type * Species + Region + (1 | plate)
##    Data: CORT
##
## REML criterion at convergence: 1353.5
##
## Scaled residuals:
##     Min      1Q  Median      3Q     Max
## -4.5355 -0.5924 -0.0176  0.6518  2.8049
##
## Random effects:
##  Groups   Name        Variance Std.Dev.
##  plate    (Intercept) 0.1334   0.3653
##  Residual             0.5335   0.7304
## Number of obs: 595, groups:  plate, 17
##
## Fixed effects:
##                                            Estimate Std. Error t value
## (Intercept)                                -0.70256    0.32563  -2.158
## Site_type                                  -0.63212    0.31534  -2.005
## SpeciesCoryphospingus_cucullatus            0.92774    0.47479   1.954
## SpeciesCyclarhis_gujanensis                 1.44378    0.30615   4.716
## SpeciesElaenia_chiriquensis                 0.33057    0.35759   0.924
## SpeciesElaenia_cristata                     1.55229    0.45974   3.376
## SpeciesEupetomena_macroura                  0.80262    0.54426   1.475
## SpeciesMyiarchus_swainsoni                  1.14620    0.43740   2.621
## SpeciesPitangus_sulphuratus                 2.09278    0.44859   4.665
```

```
## SpeciesSynallaxis_frontalis                     0.64859    0.52042   1.246
## SpeciesThraupis_sayaca                          1.41377    0.38306   3.691
## SpeciesTroglodytes_musculus                     1.61101    0.40009   4.027
## SpeciesTurdus_leucomelas                        0.55700    0.37015   1.505
## SpeciesTurdus_rufiventris                       0.88666    0.50233   1.765
## SpeciesVolatinia_jacarina                      -0.38184    0.38105  -1.002
## SpeciesZonotrichia_capensis                     1.47511    0.52207   2.825
## RegionCAMP                                     -0.44290    0.11429  -3.875
## RegionSAL                                      -0.56093    0.13618  -4.119
## Site_type:SpeciesCoryphospingus_cucullatus  0.76322    0.48642   1.569
## Site_type:SpeciesCyclarhis_gujanensis       1.56796    0.45565   3.441
## Site_type:SpeciesElaenia_chiriquensis       1.12941    0.43120   2.619
## Site_type:SpeciesElaenia_cristata           0.26509    0.40717   0.651
## Site_type:SpeciesEupetomena_macroura        0.49056    0.47478   1.033
## Site_type:SpeciesMyiarchus_swainsoni        0.50703    0.42081   1.205
## Site_type:SpeciesPitangus_sulphuratus       0.66701    0.41286   1.616
## Site_type:SpeciesSynallaxis_frontalis       1.28571    0.45319   2.837
## Site_type:SpeciesThraupis_sayaca            0.42074    0.38099   1.104
## Site_type:SpeciesTroglodytes_musculus       0.05226    0.36272   0.144
## Site_type:SpeciesTurdus_leucomelas          0.75474    0.38197   1.976
## Site_type:SpeciesTurdus_rufiventris         0.79783    0.52394   1.523
## Site_type:SpeciesVolatinia_jacarina         1.15219    0.38900   2.962
## Site_type:SpeciesZonotrichia_capensis       0.64033    0.44444   1.441
```

```
Anova(my_model,type=3)
```

```
## Analysis of Deviance Table (Type III Wald chisquare tests)
## Response: CORTf_leng_bcS
##                     Chisq Df Pr(>Chisq)
## (Intercept)        4.6551  1  0.0309615 *
## Site_type          4.0183  1  0.0450091 *
## Species           96.5434 14  2.172e-14 ***
## Region            20.4726  2  3.585e-05 ***
## Site_type:Species 39.0217 14  0.0003619 ***
## ---
## Signif. codes:  0 '***' 0.001 '**' 0.01 '*' 0.05 '.' 0.1 ' ' 1
```

Species-specific models

```
dados<- read.table("Dados-CORT2023.txt",h=T)
dataset<-dados[dados$Dataset_CORT=="Sim",]

library (AID)
library(lme4)
library(car)
```

Species with samples analyzed in **one** plate
Present in **one** region

Eupetomena macroura

```
eupe <- subset(dataset, dataset$Species=="Eupetomena_macroura")
```

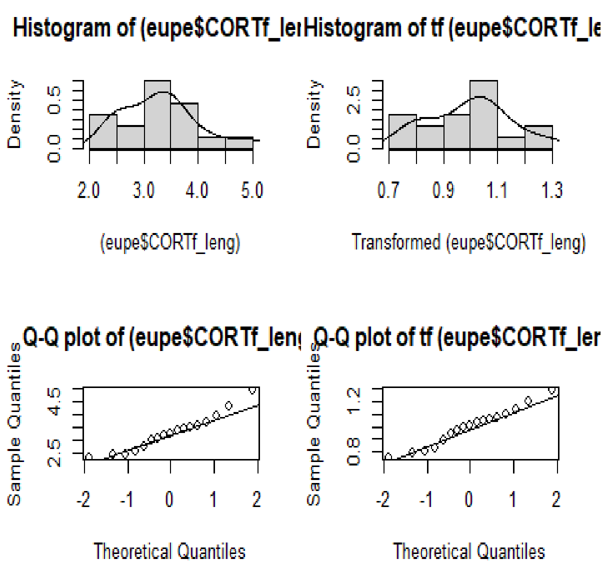


```
shapiro.test(eupe$CORTf_leng)
##  Shapiro-Wilk normality test
## data:  eupe$CORTf_leng
## W = 0.94838, p-value = 0.4314

boxcoxnc((eupe$CORTf_leng), method='sw')
##   Box-Cox power transformation
##   lambda.hat : -0.28
##   Shapiro-Wilk normality test for transformed data (alpha
= 0.05)
##   statistic  : 0.9719587
##   p.value    : 0.8517796
##   Result     : Transformed data are normal.

eupe$CORTf_leng_bc <- (((eupe$CORTf_leng)^-0.28)-1)/-0.28
eupe$CORTf_leng_bcS<- scale(eupe$CORTf_leng_bc,center=TRUE)

shapiro.test(eupe$CORTf_leng_bcS)
##  Shapiro-Wilk normality test
## data:  eupe$CORTf_leng_bcS
## W = 0.97196, p-value = 0.8518
```

```
my_model <-lm (CORTf_leng_bcS~Site_type, eupe)
summary(my_model)
## Call:
## lm(formula = CORTf_leng_bcS ~ Site_type, data = eupe)
##
## Residuals:
##     Min      1Q  Median      3Q     Max
## -1.8284 -0.8941  0.1865  0.5311  1.7842
##
## Coefficients:
##             Estimate Std. Error t value Pr(>|t|)
## (Intercept)   0.2201     0.3567   0.617    0.546
## Site_type    -0.4158     0.4902  -0.848    0.410
##
## Residual standard error: 1.009 on 15 degrees of freedom
## Multiple R-squared:  0.04576,    Adjusted R-squared:  -0.01785
## F-statistic: 0.7194 on 1 and 15 DF,  p-value: 0.4097
```

<u>Species with samples analyzed in **one** plate</u>
<u>Present in **more than one** region</u>

Synallaxis frontalis

```
syn <- subset(dataset, dataset$Species=="Synallaxis_frontalis")
```

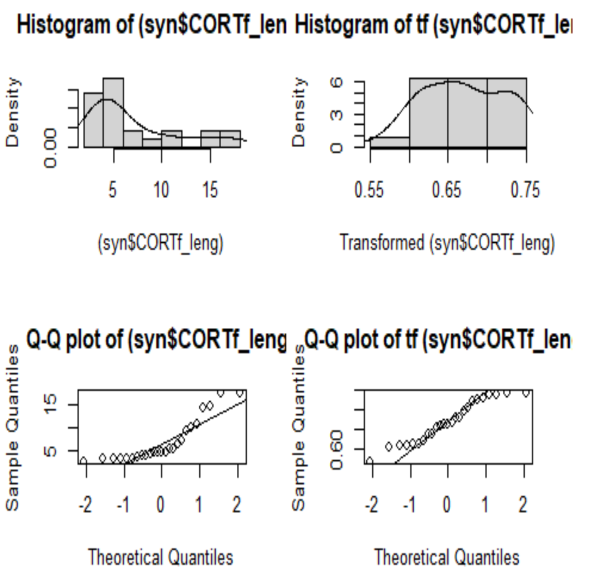


```
shapiro.test(syn$CORTf_leng)
##  Shapiro-Wilk normality test
## data:  syn$CORTf_leng
## W = 0.77982, p-value = 0.0001067
boxcoxnc((syn$CORTf_leng), method='sw')
##   Box-Cox power transformation
##   lambda.hat : -1.31
##   Shapiro-Wilk normality test for transformed data (alpha = 0
.05)
##   statistic  : 0.9399689
##   p.value    : 0.1477857
##   Result     : Transformed data are normal.

syn$CORTf_leng_bc <- (((syn$CORTf_leng)^-1.31)-1)/-1.31
syn$CORTf_leng_bcS<- scale(syn$CORTf_leng_bc,center=TRUE)

shapiro.test(syn$CORTf_leng_bcS)
##  Shapiro-Wilk normality test
## data:  syn$CORTf_leng_bcS
## W = 0.93997, p-value = 0.1478
```

```
my_model <-lm (CORTf_leng_bcS~Site_type,syn)
AIC(my_model)
## [1] 72.79266 ##Lower AIC
my_model1 <-lm (CORTf_leng_bcS~Site_type+Region,syn)
AIC(my_model1)
## [1] 74.64766

summary(my_model)
## Call:
## lm(formula = CORTf_leng_bcS ~ Site_type, data = syn)
##
## Residuals:
##     Min      1Q  Median      3Q     Max
## -2.1262 -0.6673  0.3557  0.8004  1.2360
##
## Coefficients:
##             Estimate Std. Error t value Pr(>|t|)
## (Intercept)  -0.5393     0.3626  -1.487    0.151
## Site_type     0.7490     0.4274   1.753    0.093 .
## ---
## Signif. codes:  0 '***' 0.001 '**' 0.01 '*' 0.05 '.' 0.1 ' ' 1
##
## Residual standard error: 0.9595 on 23 degrees of freedom
## Multiple R-squared:  0.1178, Adjusted R-squared:  0.07945
## F-statistic: 3.072 on 1 and 23 DF,  p-value: 0.093
```

Myiarchus swainsoni

```
mswain <- subset(dataset, dataset$Species=="Myiarchus_swainsoni")
```

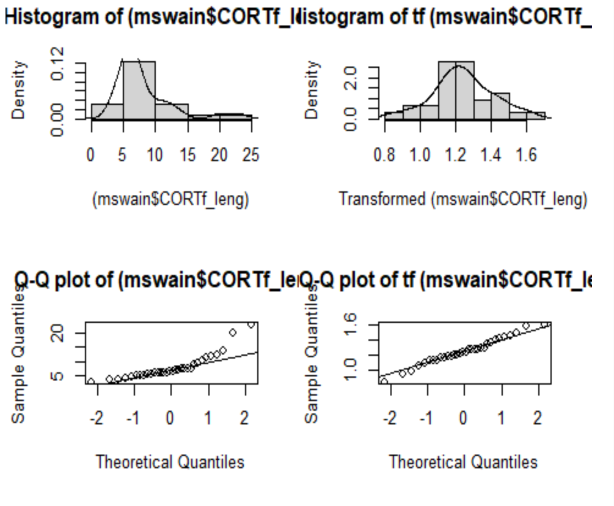


```
shapiro.test(mswain$CORTf_leng)
##  Shapiro-Wilk normality test
## data:  mswain$CORTf_leng
## W = 0.79222, p-value = 2.92e-05

boxcoxnc((mswain$CORTf_leng), method='sw')
##   Box-Cox power transformation
##   lambda.hat : -0.49
##   Shapiro-Wilk normality test for transformed data (alpha =
0.05)
##   statistic  : 0.9860125
##   p.value    : 0.9432366
##   Result     : Transformed data are normal.

mswain$CORTf_leng_bc <- (((mswain$CORTf_leng)^-0.49)-1)/-0.49
mswain$CORTf_leng_bcS<-scale(mswain$CORTf_leng_bc,center=TRUE)

shapiro.test(mswain$CORTf_leng_bcS)
##  Shapiro-Wilk normality test
## data:  mswain$CORTf_leng_bcS
## W = 0.98601, p-value = 0.9432

my_model<-lm (CORTf_leng_bcS~Site_type,mswain)
AIC(my_model)
## [1] 95.54809 ##Lower AIC
my_model1 <-lm (CORTf_leng_bcS~Site_type+Region,mswain)
AIC(my_model1)
## [1] 97.47943
summary(my_model)
## Call:
## lm(formula = CORTf_leng_bcS ~ Site_type, data = mswain)
##
## Residuals:
##      Min       1Q   Median       3Q      Max
## -2.39623 -0.56298  0.01342  0.55438  2.05170
##
## Coefficients:
##              Estimate Std. Error t value Pr(>|t|)
## (Intercept)  0.05831    0.21589   0.270    0.789
## Site_type   -0.18658    0.38619  -0.483    0.633
##
## Residual standard error: 1.013 on 30 degrees of freedom
## Multiple R-squared:  0.007721,   Adjusted R-squared:  -0.02536
## F-statistic: 0.2334 on 1 and 30 DF,  p-value: 0.6325
```

Zonotrichia capensis

```
Zcap<- subset(dados, dados$Species=="Zonotrichia_capensis")
```

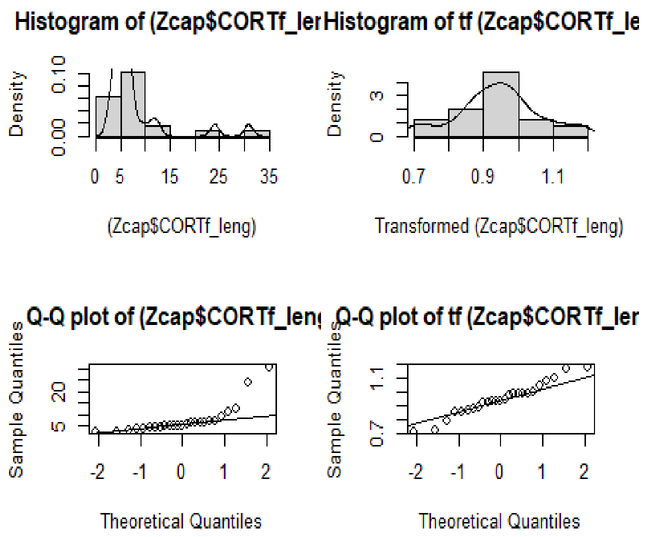


```
shapiro.test(Zcap$CORTf_leng)
##  Shapiro-Wilk normality test
## data:  Zcap$CORTf_leng
## W = 0.62177, p-value = 7.595e-07

boxcoxnc((Zcap$CORTf_leng), method='sw')
##    Box-Cox power transformation
##    lambda.hat : -0.79
##    Shapiro-Wilk normality test for transformed data (a
lpha = 0.05)
##    statistic  : 0.9694964
##    p.value    : 0.6323234
##    Result     : Transformed data are normal.

Zcap$CORTf_leng_bc <-(((Zcap$CORTf_leng)^-0.79)-1)/-0.79
Zcap$CORTf_leng_bcS<-scale(Zcap$CORTf_leng_bc,center=TRU
E)
```

```
shapiro.test(Zcap$CORTf_leng_bcS)
##  Shapiro-Wilk normality test
## data:  Zcap$CORTf_leng_bcS
## W = 0.9695, p-value = 0.6323

my_model <-lm (CORTf_leng_bcS~Site_type,Zcap)
AIC(my_model)
## [1] 75.91834 ##Lower AIC
my_model1 <-lm (CORTf_leng_bcS~Site_type+Region,Zcap)
AIC(my_model1)
## [1] 77.91812
summary(my_model)
## Call:
## lm(formula = CORTf_leng_bcS ~ Site_type, data = Zcap)
##
## Residuals:
##      Min       1Q   Median       3Q      Max
## -1.99045 -0.55842 -0.05398  0.41360  2.04897
##
## Coefficients:
##             Estimate Std. Error t value Pr(>|t|)
## (Intercept)  0.01205    0.24771   0.049    0.962
## Site_type   -0.03765    0.43790  -0.086    0.932
##
## Residual standard error: 1.021 on 23 degrees of freedom
## Multiple R-squared:  0.0003213,	Adjusted R-squared:  -0.04314
## F-statistic: 0.007392 on 1 and 23 DF,  p-value: 0.9322
```

Turdus rufiventris

```
Trufi<- subset(dados, dados$Species=="Turdus_rufiventris")
```

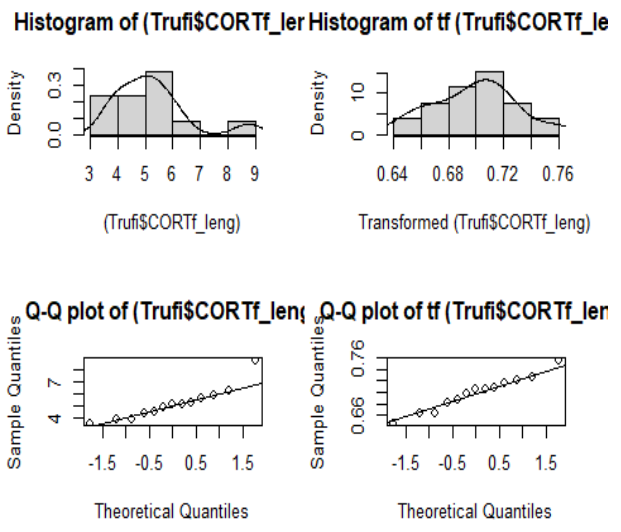


```
shapiro.test(Trufi$CORTf_leng)
##  Shapiro-Wilk normality test
## data:  Trufi$CORTf_leng
## W = 0.86876, p-value = 0.05034

boxcoxnc((Trufi$CORTf_leng), method='sw')
##   Box-Cox power transformation
##   lambda.hat : -1.23
##   Shapiro-Wilk normality test for transformed data (alpha
= 0.05)
##   statistic  : 0.9795657
##   p.value    : 0.9773488
##   Result     : Transformed data are normal.

Trufi$CORTf_leng_bc <- (((Trufi$CORTf_leng)^-1.23)-1)/-1.23
Trufi$CORTf_leng_bcS<- scale(Trufi$CORTf_leng_bc,center=TRUE)

shapiro.test(Trufi$CORTf_leng_bcS)
##  Shapiro-Wilk normality test
## data:  Trufi$CORTf_leng_bcS
## W = 0.97957, p-value = 0.9773
```

```
my_model <-lm (CORTf_leng_bcS~Site_type,Trufi)
AIC(my_model)
## [1] 37.50715 ##Lower AIC
my_model1 <-lm (CORTf_leng_bcS~Site_type+Region,Trufi)
AIC(my_model1)
## [1] 40.82965

summary(my_model)
## Call:
## lm(formula = CORTf_leng_bcS ~ Site_type, data = Trufi)
##
## Residuals:
##     Min      1Q  Median      3Q     Max
## -1.3277 -0.6615 -0.1679  0.6298  1.3529
##
## Coefficients:
##             Estimate Std. Error t value Pr(>|t|)
## (Intercept)  -0.4048     0.3124  -1.296   0.2216
## Site_type     1.0526     0.5038   2.089   0.0607 .
## ---
## Signif. codes:  0 '***' 0.001 '**' 0.01 '*' 0.05 '.' 0.1 ' ' 1
##
## Residual standard error: 0.8837 on 11 degrees of freedom
## Multiple R-squared:  0.2841, Adjusted R-squared:  0.219
## F-statistic: 4.365 on 1 and 11 DF,  p-value: 0.06071
```

Species with samples analyzed in **more than one** plate
Present in **one** region

Elaenia cristata

```
cris <- subset(dados, dados$Species=="Elaenia_cristata")
```

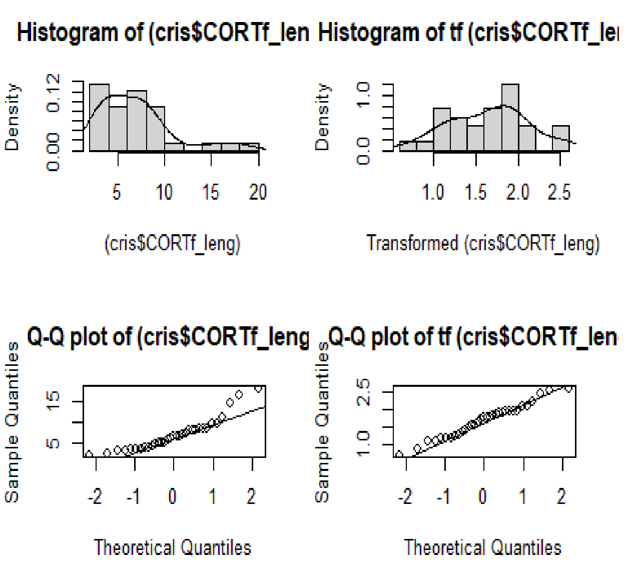


```
shapiro.test(cris$CORTf_leng)
##  Shapiro-Wilk normality test
## data:  cris$CORTf_leng
## W = 0.8723, p-value = 0.001098

boxcoxnc((cris$CORTf_leng), method='sw')
##   Box-Cox power transformation
##   lambda.hat : -0.08
##   Shapiro-Wilk normality test for transformed data (alpha
= 0.05)
##   statistic  : 0.9804077
##   p.value    : 0.7985639
##   Result     : Transformed data are normal.

cris$CORTf_leng_bc <- (((cris$CORTf_leng)^-0.08)-1)/-0.08
cris$CORTf_leng_bcS<- scale(cris$CORTf_leng_bc,center=TRUE)

shapiro.test(cris$CORTf_leng_bcS)
##  Shapiro-Wilk normality test
## data:  cris$CORTf_leng_bcS
## W = 0.98041, p-value = 0.7986
```

```
my_model <-lmer (CORTf_leng_bcS~Site_type+(1|plate),cris)
summary(my_model)
## Linear mixed model fit by REML ['lmerMod']
## Formula: CORTf_leng_bcS ~ Site_type + (1 | plate)
##    Data: cris
##
## REML criterion at convergence: 92.7
##
## Scaled residuals:
##     Min      1Q  Median      3Q     Max
## -1.8744 -0.8004  0.1386  0.4326  1.8943
##
## Random effects:
##  Groups   Name        Variance Std.Dev.
##  plate    (Intercept) 0.05535  0.2353
##  Residual             0.96648  0.9831
## Number of obs: 33, groups:  plate, 2
##
## Fixed effects:
##             Estimate Std. Error t value
## (Intercept)   0.1614     0.3280   0.492
## Site_type    -0.4562     0.3445  -1.324
##
## Correlation of Fixed Effects:
##           (Intr)
## Site_type -0.554

Anova(my_model,type=3)
## Analysis of Deviance Table (Type III Wald chisquare tests)
##
```

```
## Response: CORTf_leng_bcS
##              Chisq Df Pr(>Chisq)
## (Intercept) 0.2423  1     0.6226
## Site_type   1.7531  1     0.1855
```

Elaenia chiriquensis

```
chiri <- subset(dados, dados$Species=="Elaenia_chiriquensis")
```

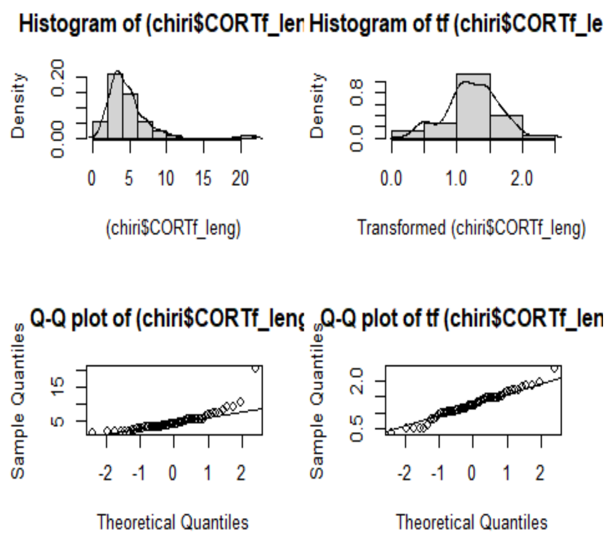


```
shapiro.test(chiri$CORTf_leng)
##  Shapiro-Wilk normality test
## data:  chiri$CORTf_leng
## W = 0.73466, p-value = 5.388e-09

boxcoxnc((chiri$CORTf_leng), method='sw')
##   Box-Cox power transformation
##   lambda.hat : -0.16
##   Shapiro-Wilk normality test for transformed data (alpha
= 0.05)
##   statistic  : 0.9793776
##   p.value    : 0.4141209
##   Result     : Transformed data are normal.

chiri$CORTf_leng_bc <- (((chiri$CORTf_leng)^-0.16)-1)/-0.16
chiri$CORTf_leng_bcS<- scale(chiri$CORTf_leng_bc,center=TRUE)

shapiro.test(chiri$CORTf_leng_bcS)
##  Shapiro-Wilk normality test
## data:  chiri$CORTf_leng_bcS
## W = 0.97938, p-value = 0.4141
my_model <-lmer (CORTf_leng_bcS~Site_type+(1|plate),chiri)
summary(my_model)
## Linear mixed model fit by REML ['lmerMod']
## Formula: CORTf_leng_bcS ~ Site_type + (1 | plate)
##    Data: chiri
##
## REML criterion at convergence: 145.5
##
## Scaled residuals:
##     Min      1Q  Median      3Q     Max
## -1.7225 -0.6287 -0.1788  0.5087  2.8318
##
## Random effects:
##  Groups   Name        Variance Std.Dev.
##  plate    (Intercept) 0.4499   0.6707
##  Residual             0.6131   0.7830
## Number of obs: 59, groups:  plate, 4
##
## Fixed effects:
##             Estimate Std. Error t value
## (Intercept)  -0.3755     0.3927  -0.956
## Site_type     0.5839     0.3806   1.534
##
## Correlation of Fixed Effects:
##           (Intr)
## Site_type -0.309
Anova(my_model,type=3)
## Analysis of Deviance Table (Type III Wald chisquare tests)
##
## Response: CORTf_leng_bcS
##              Chisq Df Pr(>Chisq)
```

```
## (Intercept) 0.9141  1       0.339
## Site_type   2.3533  1       0.125
```

Coereba flaveola

```
coer <- subset(dataset, dataset$Species=="Coereba_flaveola")

shapiro.test(coer$CORTf_leng)
```

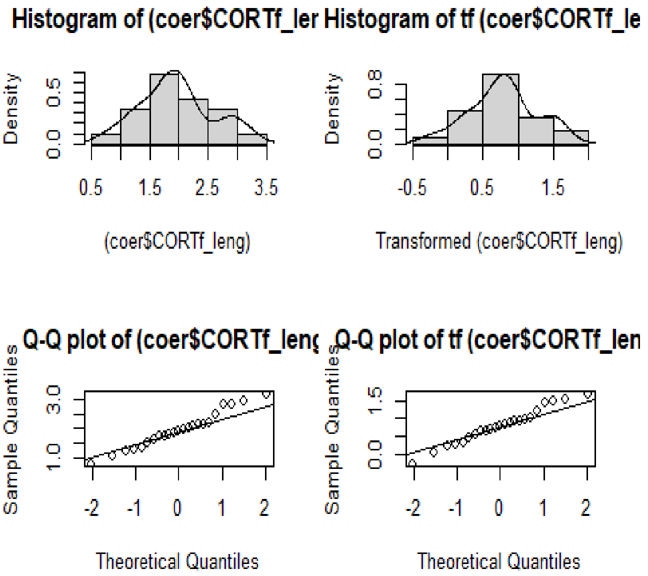


```
##  Shapiro-Wilk normality test
## data:  coer$CORTf_leng
## W = 0.97477, p-value = 0.8011

boxcoxnc((coer$CORTf_leng), method='sw')
##   Box-Cox power transformation
##   lambda.hat : 0.59
##   Shapiro-Wilk normality test for transformed data (alph
a = 0.05)
##   statistic  : 0.9819785
##   p.value    : 0.9369112
##
##   Result     : Transformed data are normal.

coer$CORTf_leng_bc <- (((coer$CORTf_leng)^0.59)-1)/0.59
coer$CORTf_leng_bcS<- scale(coer$CORTf_leng_bc,center=TRUE)

shapiro.test(coer$CORTf_leng_bcS)
##  Shapiro-Wilk normality test
## data:  coer$CORTf_leng_bcS
## W = 0.98198, p-value = 0.9369

my_model<-lmer (coer$CORTf_leng_bcS~Site_type+(1|plate),coe
r)
summary(my_model)
## Linear mixed model fit by REML ['lmerMod']
## Formula: coer$CORTf_leng_bcS ~ Site_type + (1 | plate)
##    Data: coer
##
## REML criterion at convergence: 61.7
##
## Scaled residuals:
##      Min       1Q   Median       3Q      Max
## -1.73739 -0.67613 -0.05779  0.71352  1.70847
##
## Random effects:
##  Groups   Name        Variance Std.Dev.
##  plate    (Intercept) 0.9100   0.9539
##  Residual             0.7762   0.8810
## Number of obs: 23, groups:  plate, 3
##
## Fixed effects:
##             Estimate Std. Error t value
## (Intercept)   0.6169     0.6639   0.929
## Site_type    -0.9441     0.3916  -2.411
##
## Correlation of Fixed Effects:
##           (Intr)
## Site_type -0.248

Anova(my_model,type=3)
## Analysis of Deviance Table (Type III Wald chisquare tests)
##
```

```
## Response: coer$CORTf_leng_bcS
##              Chisq Df Pr(>Chisq)
## (Intercept) 0.8637  1    0.35271
## Site_type   5.8115  1    0.01592 *
## ---
## Signif. codes:  0 '***' 0.001 '**' 0.01 '*' 0.05 '.' 0.1 ' ' 1
```

Species with samples analyzed in **more than one** plate
Present in **more than one** region

Thraupis sayaca

```
Tsay<- subset(dataset, dataset$Species=="Thraupis_sayaca")
```

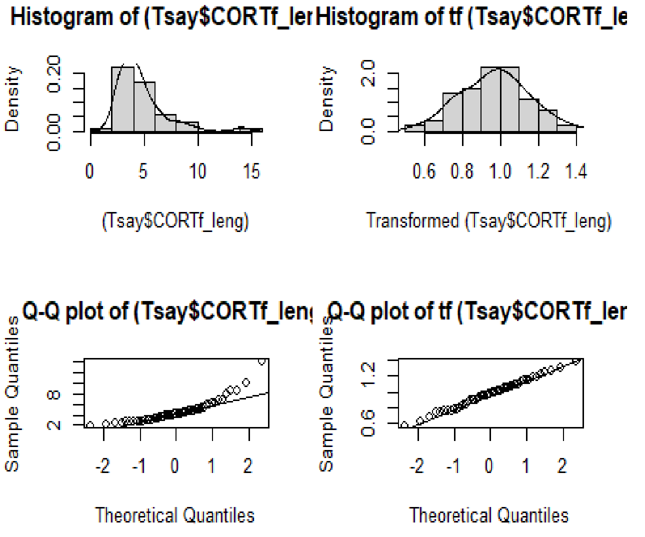


```
shapiro.test(Tsay$CORTf_leng)
##  Shapiro-Wilk normality test
## data:  Tsay$CORTf_leng
## W = 0.81987, p-value = 1.499e-06

boxcoxnc((Tsay$CORTf_leng), method='sw')
##   Box-Cox power transformation
##   lambda.hat : -0.55
##   Shapiro-Wilk normality test for transformed data (alpha
= 0.05)
##   statistic  : 0.9943266
##   p.value    : 0.9968219
##   Result     : Transformed data are normal.

Tsay$CORTf_leng_bc <- (((Tsay$CORTf_leng)^-0.55)-1)/-0.55
Tsay$CORTf_leng_bcS<- scale(Tsay$CORTf_leng_bc,center=TRUE)

shapiro.test(Tsay$CORTf_leng_bcS)
##  Shapiro-Wilk normality test
## data:  Tsay$CORTf_leng_bcS
## W = 0.99433, p-value = 0.9968
```

```
my_model <-lmer (CORTf_leng_bcS~Site_type+(1|plate),Tsay)
AIC(my_model)
## [1] 157.2701 ##Lower AIC
my_model1 <-lmer (CORTf_leng_bcS~Site_type+Region+(1|plate),Tsay)
AIC(my_model1)
## [1] 159.2277

summary(my_model)
## Linear mixed model fit by REML ['lmerMod']
## Formula: CORTf_leng_bcS ~ Site_type + (1 | plate)
##    Data: Tsay
##
## REML criterion at convergence: 149.3
##
## Scaled residuals:
##      Min       1Q   Median       3Q      Max
## -2.59605 -0.59188  0.09849  0.64950  2.14967
##
## Random effects:
##  Groups   Name        Variance Std.Dev.
##  plate    (Intercept) 0.0000   0.0000
##  Residual             0.9618   0.9807
## Number of obs: 53, groups:  plate, 4
##
```

```
## Fixed effects:
##             Estimate Std. Error t value
## (Intercept)   0.2800     0.2091   1.339
## Site_type    -0.4787     0.2734  -1.751
##
## Correlation of Fixed Effects:
##           (Intr)
## Site_type -0.765

Anova(my_model,Anova=3)
## Analysis of Deviance Table (Type II Wald chisquare tests)
## Response: CORTf_leng_bcS
##            Chisq Df Pr(>Chisq)
## Intercept 1.7931  1    0.18055
## Site_type 3.0655  1    0.07997 .
## ---
## Signif. codes:  0 '***' 0.001 '**' 0.01 '*' 0.05 '.' 0.1 ' ' 1
```

Volatinia_jacarina

```
Vjac<- subset(dataset, dataset$Species=="Volatinia_jacarina")
```

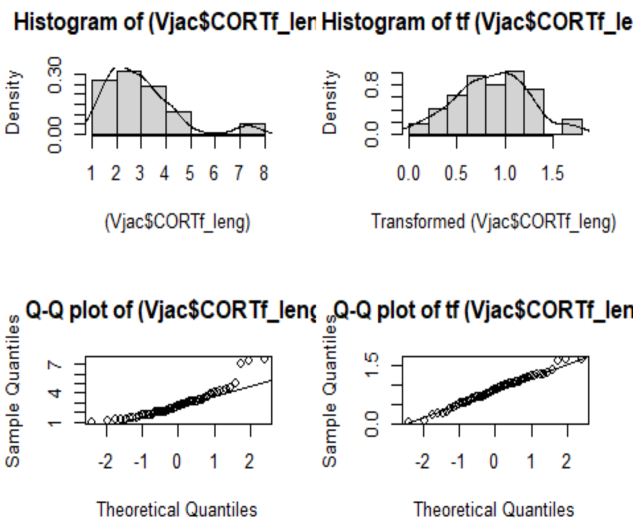


```
shapiro.test(Vjac$CORTf_leng)
##  Shapiro-Wilk normality test
## data:  Vjac$CORTf_leng
## W = 0.8671, p-value = 7.552e-06
boxcoxnc((Vjac$CORTf_leng), method='sw')
##   Box-Cox power transformation
##   lambda.hat : -0.18
##   Shapiro-Wilk normality test for transformed data (alpha
= 0.05)
##   statistic  : 0.9872723
##   p.value    : 0.7699748
##   Result     : Transformed data are normal.

Vjac$CORTf_leng_bc <- (((Vjac$CORTf_leng)^-0.18)-1)/-0.18
Vjac$CORTf_leng_bcS<- scale(Vjac$CORTf_leng_bc,center=TRUE)

shapiro.test(Vjac$CORTf_leng_bcS)
##  Shapiro-Wilk normality test
## data:  Vjac$CORTf_leng_bcS
## W = 0.98727, p-value = 0.77
```

```
my_model <-lmer (CORTf_leng_bcS~Site_type+(1|plate),Vjac)
AIC(my_model)
## [1] 183.4513 ##Lower AIC
my_model1 <-lmer (CORTf_leng_bcS~Site_type+Region+(1|plate),Vjac)
AIC(my_model1)
## [1] 185.3893

summary(my_model)
## Linear mixed model fit by REML ['lmerMod']
## Formula: CORTf_leng_bcS ~ Site_type + (1 | plate)
##    Data: Vjac
##
## REML criterion at convergence: 175.5
##
## Scaled residuals:
##      Min       1Q   Median       3Q      Max
## -2.45504 -0.74410  0.01904  0.78753  2.03824
```

```
##
## Random effects:
##  Groups   Name        Variance Std.Dev.
##  plate    (Intercept) 0.0546   0.2337
##  Residual             0.9522   0.9758
## Number of obs: 62, groups:  plate, 5
##
## Fixed effects:
##             Estimate Std. Error t value
## (Intercept)  -0.3463     0.2986  -1.160
## Site_type     0.4845     0.3014   1.607
##
## Correlation of Fixed Effects:
##           (Intr)
## Site_type -0.799

Anova(my_model,Anova=3)
## Analysis of Deviance Table (Type II Wald chisquare tests)
## Response: CORTf_leng_bcS
##            Chisq Df Pr(>Chisq)
## Intercept 1.3451  1     0.2461
## Site_type 2.5835  1     0.1080
```

Cyclarhis gujanensis

```
Cguj <- subset(dados, dados$Species=="Cyclarhis_gujanensis")
```

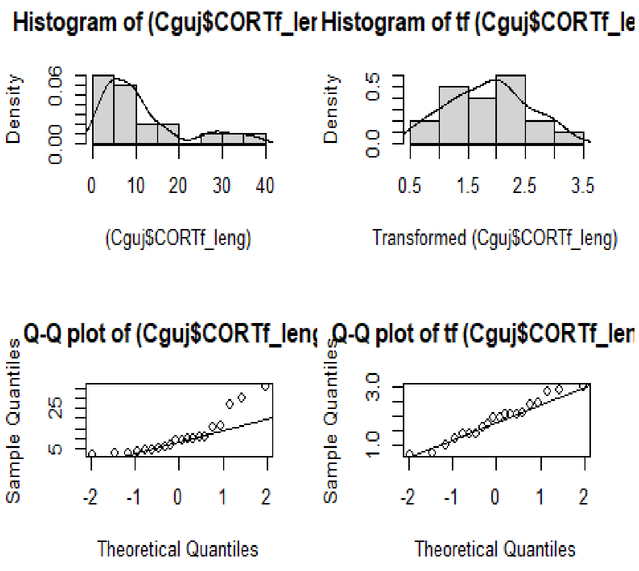


```
shapiro.test(Cguj$CORTf_leng)
##  Shapiro-Wilk normality test
## data:  Cguj$CORTf_leng
## W = 0.80187, p-value = 0.0009215

boxcoxnc((Cguj$CORTf_leng), method='sw')
##   Box-Cox power transformation
##   lambda.hat : -0.1
##   Shapiro-Wilk normality test for transformed data (alph
a = 0.05)
##   statistic  : 0.9673288
##   p.value    : 0.6977748
##   Result     : Transformed data are normal.

Cguj$CORTf_leng_bc <- (((Cguj$CORTf_leng)^-0.1)-1)/-0.1
Cguj$CORTf_leng_bcS<- scale(Cguj$CORTf_leng_bc,center=TRUE)

shapiro.test(Cguj$CORTf_leng_bcS)
##  Shapiro-Wilk normality test
## data:  Cguj$CORTf_leng_bcS
## W = 0.96733, p-value = 0.6978
```

```
my_model <-lmer (CORTf_leng_bcS~Site_type+(1|plate),Cguj)
AIC(my_model)
## [1] 59.50823 ##Lower AIC
my_model1 <-lmer (CORTf_leng_bcS~Site_type+Region+(1|plate),Cguj)
AIC(my_model1)
## [1] 59.75913
summary(my_model)
## Linear mixed model fit by REML ['lmerMod']
## Formula: CORTf_leng_bcS ~ Site_type + (1 | plate)
##    Data: Cguj
##
## REML criterion at convergence: 51.5
```

```
##
## Scaled residuals:
##     Min      1Q  Median      3Q     Max
## -2.3745 -0.3293 -0.1587  0.5174  1.5657
##
## Random effects:
##  Groups   Name        Variance Std.Dev.
##  plate    (Intercept) 0.2694   0.5190
##  Residual             0.7573   0.8703
## Number of obs: 20, groups:  plate, 2
##
## Fixed effects:
##             Estimate Std. Error t value
## (Intercept)  -0.2490     0.4982   -0.50
## Site_type     0.9379     0.3892    2.41
##
## Correlation of Fixed Effects:
##           (Intr)
## Site_type -0.391

Anova(my_model,Anova=3)
## Analysis of Deviance Table (Type II Wald chisquare tests)
##
## Response: CORTf_leng_bcS
##            Chisq Df Pr(>Chisq)
## Site_type 5.8075  1    0.01596 *
## Signif. codes:  0 '***' 0.001 '**' 0.01 '*' 0.05 '.' 0.1 ' ' 1
```

Pitangus sulphuratus

```
Psul <- subset(dados, dados$Species=="Pitangus_sulphuratus")
```

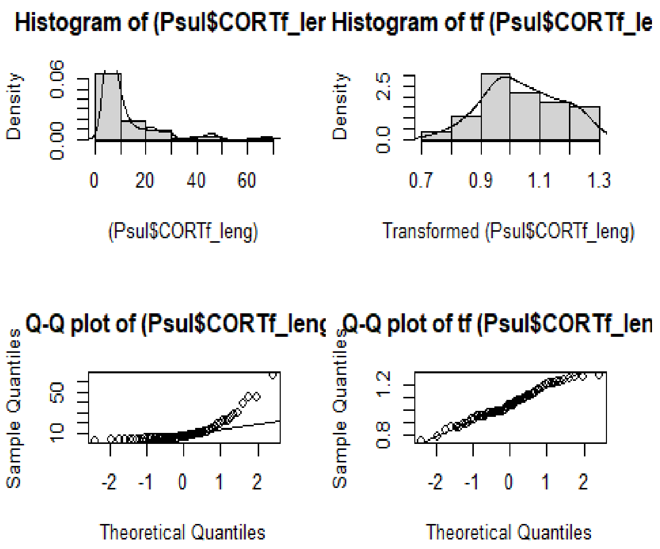


```
shapiro.test(Psul$CORTf_leng)
##  Shapiro-Wilk normality test
## data:  Psul$CORTf_leng
## W = 0.66373, p-value = 7.833e-11
boxcoxnc((Psul$CORTf_leng), method='sw')
##   Box-Cox power transformation
##   lambda.hat : -0.75
##   Shapiro-Wilk normality test for transformed data (al
pha = 0.05)
##   statistic  : 0.978525
##   p.value    : 0.3274824
##   Result     : Transformed data are normal.

Psul$CORTf_leng_bc <- (((Psul$CORTf_leng)^-0.75)-1)/-0.75
Psul$CORTf_leng_bcS<- scale(Psul$CORTf_leng_bc,center=TRU
E)

shapiro.test(Psul$CORTf_leng_bcS)
##  Shapiro-Wilk normality test
## data:  Psul$CORTf_leng_bcS
## W = 0.97853, p-value = 0.3275
my_model <-lmer (CORTf_leng_bcS~Site_type+(1|plate),Psul)
AIC(my_model)
## [1] 188.5137 ##Lower AIC
my_model1 <-lmer (CORTf_leng_bcS~Site_type+Region+(1|plate),Psul)
AIC(my_model1)
## [1] 191.7235
```

```
summary(my_model)
## Linear mixed model fit by REML ['lmerMod']
## Formula: CORTf_leng_bcS ~ Site_type + (1 | plate)
##    Data: Psul
##
## REML criterion at convergence: 180.5
##
## Scaled residuals:
##     Min      1Q  Median      3Q     Max
## -2.0374 -0.6998 -0.2059  0.7802  2.2549
##
## Random effects:
##  Groups   Name        Variance Std.Dev.
##  plate    (Intercept) 0.1613   0.4016
##  Residual             0.9462   0.9727
## Number of obs: 64, groups:  plate, 2
##
## Fixed effects:
##             Estimate Std. Error t value
## (Intercept)  -0.1751     0.4358  -0.402
## Site_type     0.1219     0.3504   0.348
##
## Correlation of Fixed Effects:
##           (Intr)
## Site_type -0.701


Anova(my_model,Anova=3)
## Analysis of Deviance Table (Type II Wald chisquare tests)
## Response: CORTf_leng_bcS
##            Chisq Df Pr(>Chisq)
## Intercept 0.1615  1     0.6878
## Site_type 0.1211  1     0.7279
```

Troglodytes musculus

```
Tmus <- subset(dados, dados$Species=="Troglodytes_musculus")
```

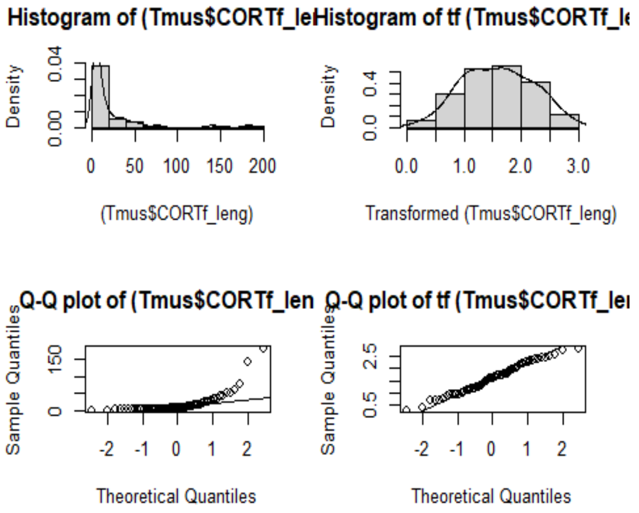


```
shapiro.test(Tmus$CORTf_leng)
##  Shapiro-Wilk normality test
## data:  Tmus$CORTf_leng
## W = 0.54016, p-value = 3.624e-13
boxcoxnc((Tmus$CORTf_leng), method='sw')
##   Box-Cox power transformation
##   lambda.hat : -0.26
##   Shapiro-Wilk normality test for transformed data (alph
a = 0.05)
##   statistic  : 0.9838867
##   p.value    : 0.5377738
##   Result     : Transformed data are normal.

Tmus$CORTf_leng_bc <- (((Tmus$CORTf_leng)^-0.26)-1)/-0.26
Tmus$CORTf_leng_bcS<- scale(Tmus$CORTf_leng_bc,center=TRUE)

shapiro.test(Tmus$CORTf_leng_bcS)
##  Shapiro-Wilk normality test
## data:  Tmus$CORTf_leng_bcS
## W = 0.98389, p-value = 0.5378
```

```
my_model <-lmer (CORTf_leng_bcS~Site_type+(1|plate),Tmus)
AIC(my_model)
## [1] 188.5142
my_model1 <-lmer (CORTf_leng_bcS~Site_type+Region+(1|plate),Tmus)
AIC(my_model1)
## [1] 170.3643 ##Lower AIC

summary(my_model1)
## Linear mixed model fit by REML ['lmerMod']
## Formula: CORTf_leng_bcS ~ Site_type + Region + (1 | plate)
##    Data: Tmus
##
## REML criterion at convergence: 158.4
##
## Scaled residuals:
##      Min       1Q   Median       3Q      Max
## -2.86653 -0.49344  0.08633  0.52804  2.03156
##
## Random effects:
##  Groups   Name        Variance Std.Dev.
##  plate    (Intercept) 0.0000   0.0000
##  Residual             0.5966   0.7724
## Number of obs: 67, groups:  plate, 3
##
## Fixed effects:
##              Estimate Std. Error t value
## (Intercept)   1.1056     0.1969   5.615
## Site_type    -0.4200     0.1890  -2.222
## RegionCAMP   -1.0474     0.2317  -4.520
## RegionSAL    -1.5085     0.2388  -6.317
##
## Correlation of Fixed Effects:
##            (Intr) St_typ RgCAMP
## Site_type  -0.480
## RegionCAMP -0.646 -0.016
## RegionSAL  -0.617 -0.036  0.540

Anova(my_model1,Anova=3)
## Analysis of Deviance Table (Type II Wald chisquare tests)
## Response: CORTf_leng_bcS
##              Chisq Df Pr(>Chisq)
## Site_type   4.9371  1    0.02629 *
## Region     41.6485  2  9.039e-10 ***
## ---
## Signif. codes:  0 '***' 0.001 '**' 0.01 '*' 0.05 '.' 0.1 ' ' 1
```

Turdus leucomelas

```
Tleuco<- subset(dados, dados$Species=="Turdus_leucomelas")
```

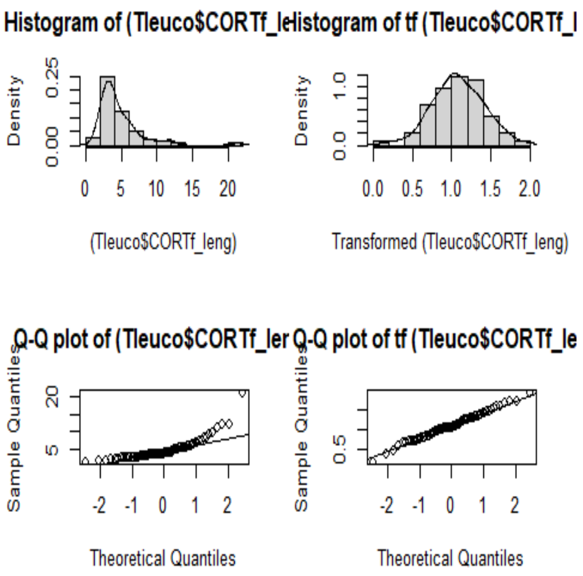


```
shapiro.test(Tleuco$CORTf_leng)
##  Shapiro-Wilk normality test
## data:  Tleuco$CORTf_leng
## W = 0.74737, p-value = 8.365e-10

boxcoxnc((Tleuco$CORTf_leng), method='sw')
##   Box-Cox power transformation
##   lambda.hat : -0.33
##   Shapiro-Wilk normality test for transformed data (alpha =
0.05)
##   statistic  : 0.9950517
##   p.value    : 0.9944502
##   Result     : Transformed data are normal.

Tleuco$CORTf_leng_bc <- (((Tleuco$CORTf_leng)^-0.33)-1)/-0.33
Tleuco$CORTf_leng_bcS<- scale(Tleuco$CORTf_leng_bc,center=TRUE)

shapiro.test(Tleuco$CORTf_leng_bcS)
##  Shapiro-Wilk normality test
## data:  Tleuco$CORTf_leng_bcS
## W = 0.99505, p-value = 0.9945

my_model <-lmer (CORTf_leng_bcS~Site_type+(1|plate),Tleuco)
Anova(my_model,Anova=3)
AIC(my_model)
## [1] 210.6326
my_model1 <-lmer (CORTf_leng_bcS~Site_type+Region+(1|plate),Tleuco)
AIC(my_model1)
## [1] 206.2949 ##Lower AIC

summary(my_model1)
## Linear mixed model fit by REML ['lmerMod']
## Formula: CORTf_leng_bcS ~ Site_type + Region + (1 | plate)
##    Data: Tleuco
##
## REML criterion at convergence: 194.3
##
## Scaled residuals:
##      Min       1Q   Median       3Q      Max
## -2.07323 -0.61666 -0.09437  0.74082  2.26760
##
## Random effects:
##  Groups   Name        Variance Std.Dev.
##  plate    (Intercept) 0.2062   0.4541
##  Residual             0.8166   0.9037
## Number of obs: 72, groups:  plate, 4
##
## Fixed effects:
##             Estimate Std. Error t value
## (Intercept)   0.7011     0.4397   1.595
## Site_type     0.1782     0.2798   0.637
## RegionCAMP   -1.2317     0.4643  -2.653
## RegionSAL    -1.1338     0.3736  -3.035
##
## Correlation of Fixed Effects:
##            (Intr) St_typ RgCAMP
## Site_type  -0.415
```

```
## RegionCAMP -0.610  0.022
## RegionSAL  -0.707  0.069  0.765

Anova(my_model1,Anova=3)
## Analysis of Deviance Table (Type II Wald chisquare tests)
## Response: CORTf_leng_bcS
##            Chisq Df Pr(>Chisq)
## Site_type 0.4055  1   0.524273
## Region    9.4731  2   0.008769 **
## ---
## Signif. codes:  0 '***' 0.001 '**' 0.01 '*' 0.05 '.' 0.1 ' ' 1
```

Coryphospingus cucullatus

```
Ccuc<- subset(dataset, dataset$Species=="Coryphospingus_cucullatus")
```

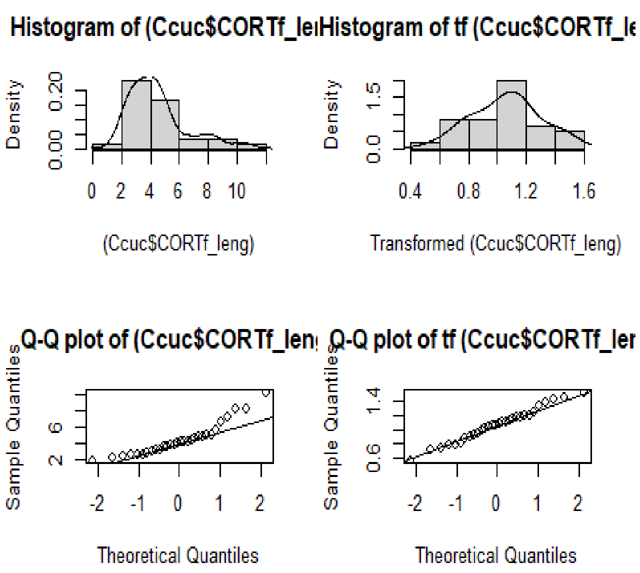


```
shapiro.test(Ccuc$CORTf_leng)
##  Shapiro-Wilk normality test
## data:  Ccuc$CORTf_leng
## W = 0.88183, p-value = 0.003107

boxcoxnc((Ccuc$CORTf_leng), method='sw')
##   Box-Cox power transformation
##   lambda.hat : -0.39
##   Shapiro-Wilk normality test for transformed data (alph
a = 0.05)
##   statistic  : 0.9868064
##   p.value    : 0.9638252
##   Result     : Transformed data are normal.

Ccuc$CORTf_leng_bc <- (((Ccuc$CORTf_leng)^-0.39)-1)/-0.39
Ccuc$CORTf_leng_bcS<- scale(Ccuc$CORTf_leng_bc,center=TRUE)

shapiro.test(Ccuc$CORTf_leng_bcS)
##  Shapiro-Wilk normality test
## data:  Ccuc$CORTf_leng_bcS
## W = 0.98681, p-value = 0.9638
my_model <-lmer (CORTf_leng_bcS~Site_type+(1|plate),Ccuc)
AIC(my_model)
## [1] 77.17611
my_model1 <-lmer (CORTf_leng_bcS~Site_type+Region+(1|plate),Ccuc)
AIC(my_model1)
## [1] 76.34411 ##Lower AIC

summary(my_model1)
## Linear mixed model fit by REML ['lmerMod']
## Formula: CORTf_leng_bcS ~ Site_type + Region + (1 | plate)
##    Data: Ccuc
##
## REML criterion at convergence: 66.3
##
## Scaled residuals:
##      Min       1Q   Median       3Q      Max
## -1.76614 -0.57052 -0.04634  0.48670  2.26141
##
## Random effects:
##  Groups   Name        Variance Std.Dev.
##  plate    (Intercept) 2.439    1.5617
##  Residual             0.488    0.6986
## Number of obs: 30, groups:  plate, 2
##
```

```
## Fixed effects:
##              Estimate Std. Error t value
## (Intercept)   1.1355     1.2278   0.925
## Site_type     0.9843     0.4371  -2.252
## RegionCAMP   -0.8233     0.5548  -1.484
##
## Correlation of Fixed Effects:
##            (Intr) St_typ
## Site_type  -0.127
## RegionCAMP -0.374 -0.181

Anova(my_model1,Anova=3)
## Analysis of Deviance Table (Type II Wald chisquare tests)
## Response: CORTf_leng_bcS
##            Chisq Df Pr(>Chisq)
## Site_type 5.0713  1    0.02432 *
## Region    2.2024  1    0.13780
## ---
## Signif. codes:  0 '***' 0.001 '**' 0.
```

D. Data used in Population specific model. Including raw data, R scripts, and outputs.

Suppl. Table D. Data used in Population specific model. Includes $CORT_f$ mean values (pg mm^{-1}), standard deviation and number of samples for each population in airport-affected and quiet control sites. Effect size was calculated using Nakagawa and Cuthill (2007)*. Degree of urbanity is described in Alquezar *et al.* (2020). Song Frequency Index was calculated as Song Frequency corrected by Body weight.

**Species**	**Region**	**$CORT_f$ airport-affected**			**$CORT_f$ quiet control**			**$CORT_f$ effect-size**	**Degree of urbanity**	**Song Frequency (Peak- Hz)**	**Body Weight (g)**	**Song Frequency Index (residuals)**
		**Mean**	**sd**	**N**	**Mean**	**sd**	**N**					
C. flaveola	SAL	1.663	0.62	9	2.127	0.58	14	-0.1247	0.191	7717.48	9.14	2166.01
C. cucullatus	CAMP	4.777	2.06	20	3.653	1.99	8	0.3225	-0.256	2429.01	14.71	-2123.11
C. gujanensis	SAL	14.311	11.38	9	5.340	2.88	6	0.7584	0.131	2580.97	26.2	-1085.41
E. chiriquensis	BRAS	5.901	3.56	22	3.922	2.17	37	0.2636	-0.698	2861.22	15.13	-1640.37
E. cristata	BRAS	5.993	3.37	16	7.672	4.31	17	-0.3146	-0.698	3213.27	18.58	-944.14
E. macroura	SAL	3.143	0.54	9	3.477	0.85	8	-0.1213	0.000	5401.78	7.48	-668.81
M. swainsoni	BRAS	6.968	3.08	8	8.663	5.41	17	-0.1809	0.245	1690.76	24.1	-2086.18
P. sulphuratus	SAL	13.232	14.27	41	9.112	4.96	10	0.2784	0.156	3303.19	49.82	332.67
S. frontalis	CAMP	7.492	4.84	15	4.512	0.52	5	0.5710	0.334	3018.21	15.67	-1421.61
T. sayaca	CAMP	3.890	0.53	11	5.884	3.25	13	-0.4030	0.254	4008.01	31.86	580.71
T. sayaca	SAL	3.942	1.26	20	4.813	2.42	9	-0.2566	0.254	4008.01	33.23	628.79
T. musculus	BRAS	28.382	40.87	10	54.271	49.29	10	-0.4077	0.044	5887.18	12.75	1064.77
T. musculus	CAMP	11.417	11.80	13	11.172	10.67	12	0.0154	0.044	5887.18	11.83	913.58
T. musculus	SAL	3.770	1.07	12	6.800	4.86	10	-0.5628	0.044	5887.18	11.02	763.84
T. leucomelas	CAMP	4.323	1.76	8	4.575	3.39	14	-0.0417	0.046	4200.00	67.28	1478.31
T. leucomelas	SAL	3.685	1.124	19	3.894	1.97	16	-0.0479	0.046	4200.00	62.66	1423.08
V. jacarina	BRAS	3.157	1.72	30	2.541	1.25	12	0.1646	0.138	6855.70	9.45	1384.31
Z. capensis	CAMP	9.306	9.70	7	7.202	5.32	15	0.1650	-0.337	3231.66	20.62	-766.45

$*Hedge's\ d = \frac{m_2 - m_1}{s_{pooled}};\ s_{pooled} = \sqrt{\frac{(n_2-1)s_2^2 + (n_1-1)s_1^2}{n_1+n_2-1}}$

Alquezar RD, Macedo RH, Sierro J, Gil D (2020) Lack of consistent responses to aircraft noise in dawn song timing of bird populations near tropical airports. *Behav Ecol Sociobiol* 74: 88.

Nakagawa S, Cuthill IC (2007) Effect size, confidence interval and statistical significance: A practical guide for biologists. *Biol Rev* 82: 591–605.

Global Model 2

```
library(lme4)
library(MuMIn)
library(car)
library(AID)

## Song frequency corrected by Body weight
```

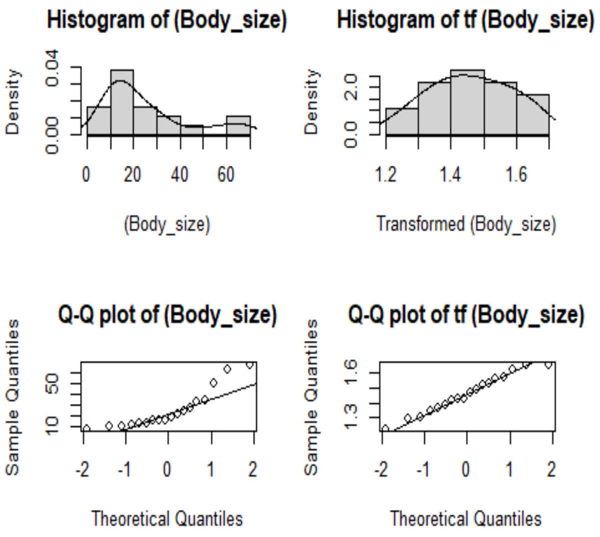


```
shapiro.test(Body_size)
##  Shapiro-Wilk normality test
## data:  Body_size
## W = 0.80874, p-value = 0.002024

boxcoxnc((Body_size), method='sw')
##   Box-Cox power transformation
##   lambda.hat : -0.54
##   Shapiro-Wilk normality test for transformed data (alpha =
0.05)
##   statistic  : 0.9724422
##   p.value    : 0.8421552
##   Result     : Transformed data are normal.

Body_weight_bc <- (((Body_size-0.54)^-0.54)-1)/-0.54
Body_weight_bcS<- scale(Body_weight_bc,center=TRUE)
shapiro.test(Body_weight_bcS)
##  Shapiro-Wilk normality test
## data:  Body_weight_bcS
## W = 0.97284, p-value = 0.8491
```

```
shapiro.test(Song_frequency) #Normal data
##  Shapiro-Wilk normality test
## data:  Song_frequency
## W = 0.9426, p-value = 0.3208

A<-lm(Song_frequency~Body_weight_bcS)
summary(A)
## Call:
## lm(formula = Song_frequency ~ Body_weight_bcS)
## Residuals:
##     Min      1Q  Median      3Q     Max
## -2123.1 -1050.1   456.7  1027.0  2166.0
##
## Coefficients:
##                 Estimate Std. Error t value Pr(>|t|)
## (Intercept)       4243.4      327.5  12.957 6.72e-10 ***
## Body_weight_bcS   -985.7      337.0  -2.925  0.00991 **
## ---
## Signif. codes:  0 '***' 0.001 '**' 0.01 '*' 0.05 '.' 0.1 ' ' 1
##
## Residual standard error: 1389 on 16 degrees of freedom
## Multiple R-squared:  0.3484, Adjusted R-squared:  0.3077
## F-statistic: 8.556 on 1 and 16 DF,  p-value: 0.009911

residuals(A)
##          1          2          3          4          5          6          7
##  2166.0131 -2123.1105 -1085.4188 -1640.3729  -944.1436  -668.8165 -2086.1869
##          8          9         10         11         12         13         14
##   332.6727 -1421.6174   580.7129   628.7949  1064.7797   913.5868   763.8498
##         15         16         17         18
##  1478.3112  1423.0808  1384.3166  -766.4519
```

```
shapiro.test(residuals(A))
##  Shapiro-Wilk normality test
## data:  residuals(A)
## W = 0.93213, p-value = 0.2117

Song_Freq_Index<-residuals(A)

shapiro.test(X.CORT..Hedgesd) #normal data
##  Shapiro-Wilk normality test
## data:  X.CORT..Hedgesd
## W = 0.97365, p-value = 0.8629

CORT<-X.CORT..Hedgesd
```

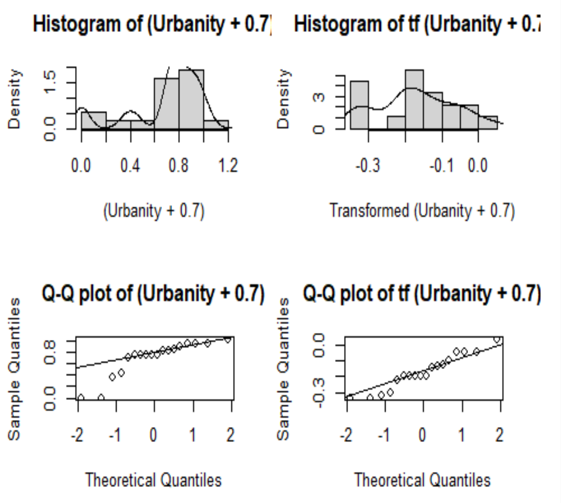


```
shapiro.test(Urbanity)
##  Shapiro-Wilk normality test
## data:  Urbanity
## W = 0.80679, p-value = 0.001897
min(Urbanity)
## [1] -0.698
boxcoxnc((Urbanity+0.7), method='sw', lambda = seq(-4,4,0.1))
##   Box-Cox power transformation
##   lambda.hat : 3
##   Shapiro-Wilk normality test for transformed data (alpha = 0.0
5)
##   statistic  : 0.9470191
##   p.value    : 0.3801889
##   Result     : Transformed data are normal.

Urbanity_bc <- (((Urbanity+0.7)^3)-1)/3
Urbanity_bcS<- scale(Urbanity_bc,center=TRUE)
shapiro.test(Urbanity_bcS)
##  Shapiro-Wilk normality test
## data:  Urbanity_bcS
## W = 0.94702, p-value = 0.3802

my_model<-dredge(lm(CORT~Song_Freq_Index+Urbanity_bcS+Region,data=global,na.action="na.fail"))
print(my_model)
## Global model call: lm(formula = CORT ~ Song_Freq_Index + Urbanity_bcS + Region,
##     data = global, na.action = "na.fail")
## ---
## Model selection table
##       (Int) Rgn Sng_Frq_Ind    Urb_bcS df logLik AICc delta weight
## 3  0.004322      -0.0001116            3 -4.299 16.3  0.00  0.457
## 1  0.004322                            2 -6.136 17.1  0.76  0.312
## 7  0.004322      -0.0001182  0.0423400 4 -4.143 19.4  3.05  0.099
## 5  0.004322                  0.0092540 3 -6.130 20.0  3.66  0.073
## 4 -0.150600   +  -0.0001252            5 -3.395 21.8  5.48  0.030
## 2 -0.094980   +                        4 -5.641 22.4  6.05  0.022
## 6 -0.095070   +             -0.0002415 5 -5.641 26.3  9.97  0.003
## 8 -0.142900   +  -0.0001280  0.0247100 6 -3.339 26.3 10.00  0.003
## Models ranked by AICc(x)

a <-get.models(my_model,subset=delta<2)
summary(model.avg(a))
## Call:
## model.avg(object = a)
##
## Component model call:
## lm(formula = CORT ~ <2 unique rhs>, data = global, na.action = na.fail)
##
## Component models:
##       df logLik  AICc delta weight
```

```
## 1       3  -4.30 16.31  0.00   0.59
## (Null)  2  -6.14 17.07  0.76   0.41
##
## Term codes:
## Song_Freq_Index
##               1
##
## Model-averaged coefficients:
## (full average)
##                   Estimate Std. Error Adjusted SE z value Pr(>|z|)
## (Intercept)      4.322e-03  7.918e-02   8.546e-02   0.051    0.960
## Song_Freq_Index -6.630e-05  7.104e-05   7.344e-05   0.903    0.367
##
## (conditional average)
##                   Estimate Std. Error Adjusted SE z value Pr(>|z|)
## (Intercept)      4.322e-03  7.918e-02   8.546e-02   0.051   0.9597
## Song_Freq_Index -1.116e-04  5.863e-05   6.342e-05   1.760   0.0784 .
## ---
## Signif. codes:  0 '***' 0.001 '**' 0.01 '*' 0.05 '.' 0.1 ' ' 1

plot(Song_Freq_Index,CORT)
abline(0.004322,-0.0001116)
```

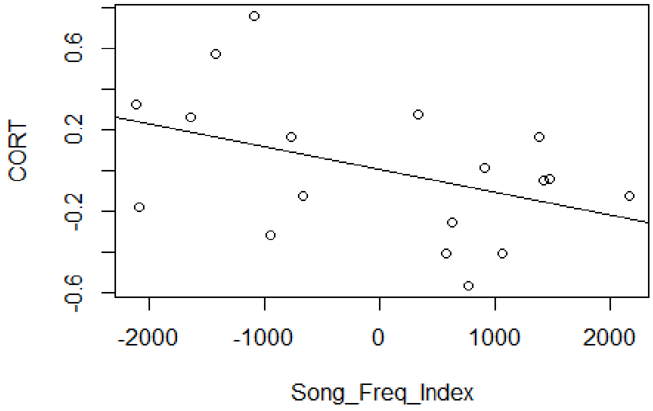

Supplement: Web_Material_coad079 [file web_material_coad079.pdf]
